# Supplementary figures and images for: The N-terminus of varicella-zoster virus glycoprotein B has a functional role in fusion
Source: PLoS Pathog. 2021 Jan 7;17(1):e1008961. doi: 10.1371/journal.ppat.1008961 (PMC7817050; doi:10.1371/journal.ppat.1008961)

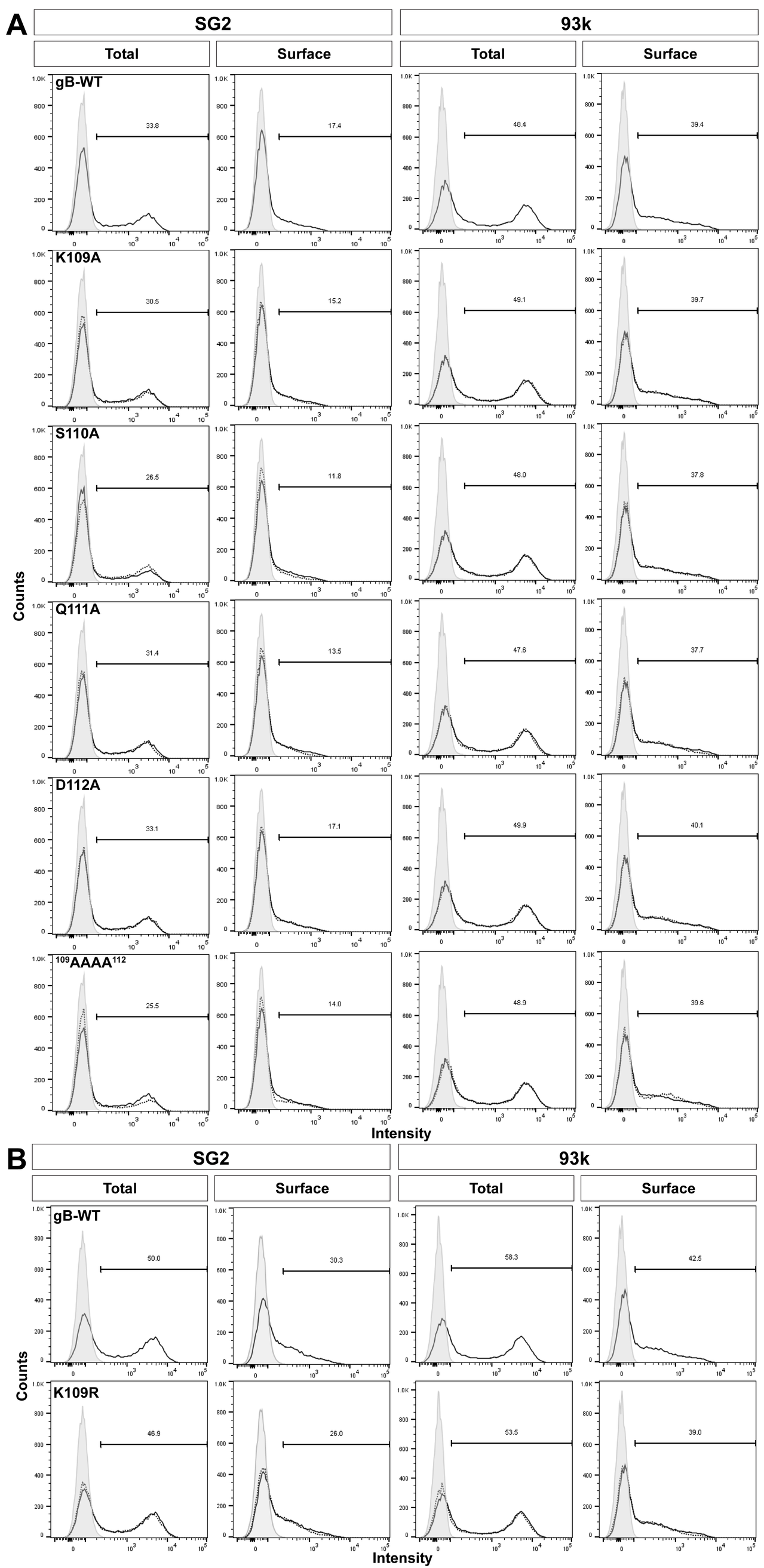

Supplement: S1 Fig — A and B–Histograms for total and surface stained gB for wild type (WT-gB) and the N-terminal mutants K109A, K109R, S110A, Q111A, D112A and 109AAAA112 are presented with fluorescence intensity along the abscissa and frequency along the ordinate. In all of the histograms the negative control (gH) is shaded grey, the positive control (WT-gB) a solid line and each of the mutants a dotted line. Numbers above the gates (|—|) are the percentage of positive events for either surface stained or total gB as determined by either SG2 of 93k staining. (TIF) [file ppat.1008961.s001.tif]

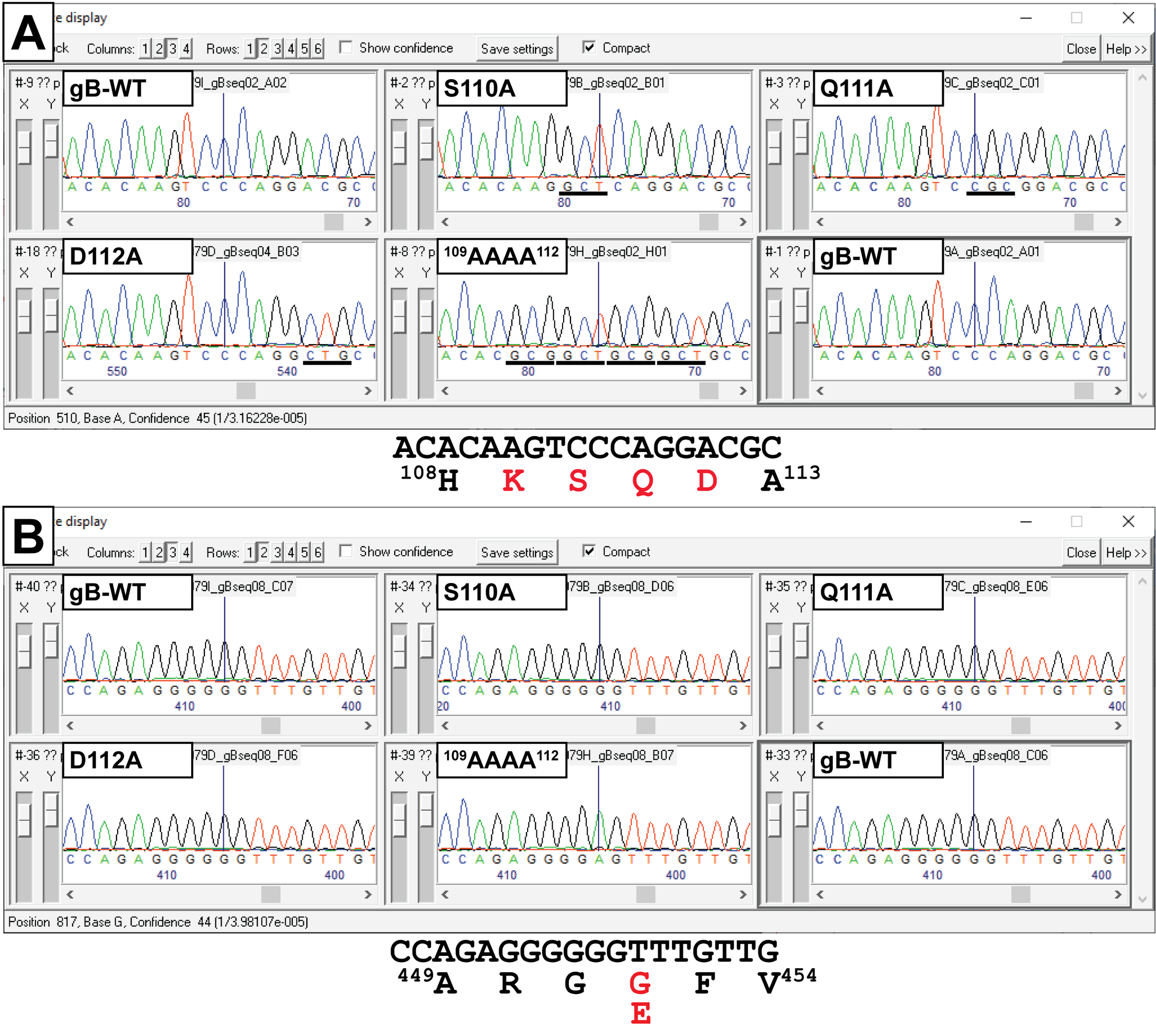

Supplement: S2 Fig — PCR products generated from ORF31[gB] of virus stocks were sequenced to determine that only the expected mutations were present for each of the VZV gB N-terminal mutants generated from the transfection of MeWo cells with BACs. Electropherograms in the N-terminal (A) and compensatory mutation (B) regions are shown with the codon for each of the alanine substitutions underlined. A–The coding DNA sequence and translated amino acids for gB-WT are provided under each panel with the substituted amino acids highlighted in red. B–A G→A transition occurred in the 109AAAA112 mutant resulting in a G452E substitution in gB DII. (TIF) [file ppat.1008961.s002.tif]

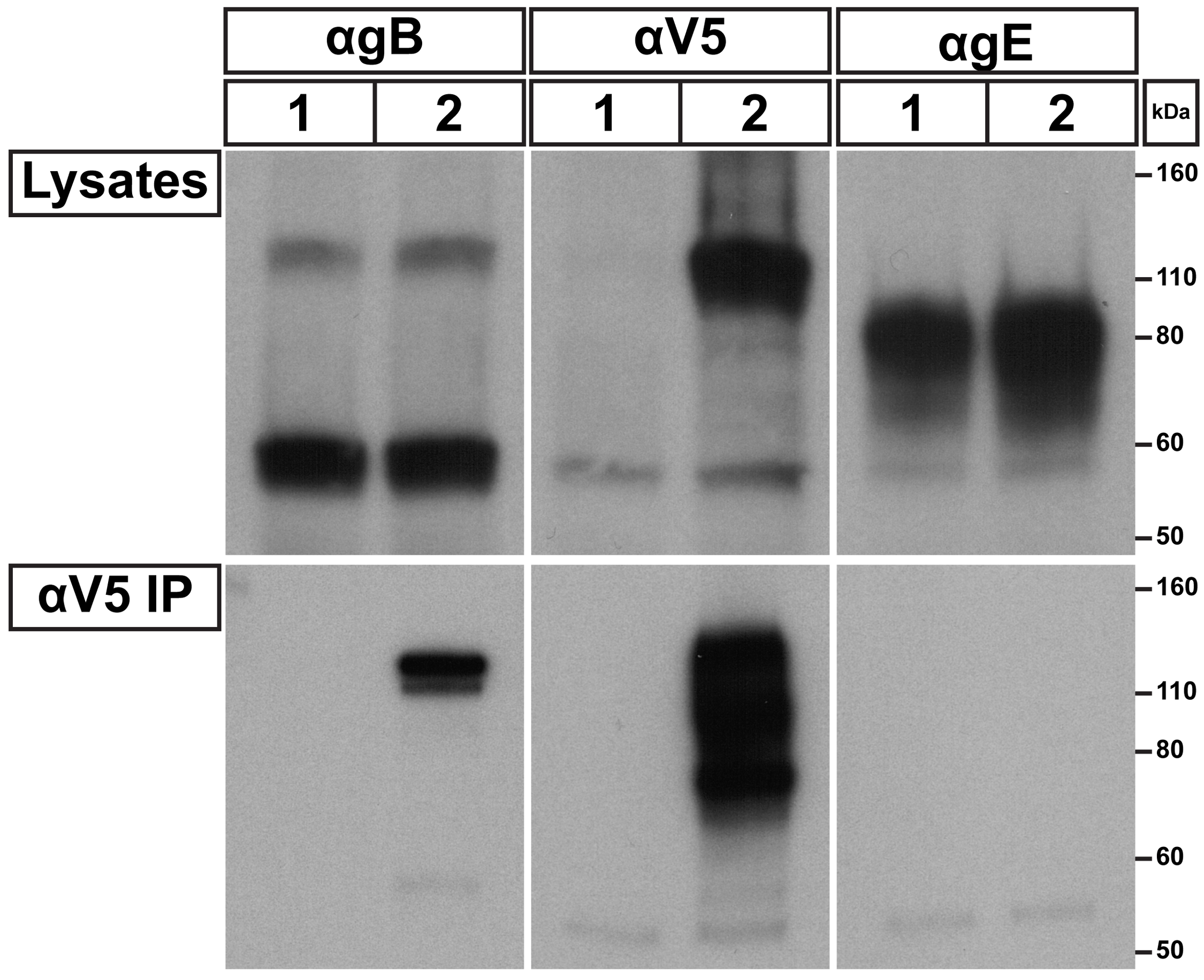

Supplement: S3 Fig — Western blots of lysates or anti-V5 immunoprecipitates (αV5) from CHO cells transfected with plasmids expressing either gB/gH-WT/gL/gE/gI (1) or gB/gH-V5/gL/gE/gI (2). Western blots were performed using the same samples with the anti-gB human mAb 93k (αgB), mouse mAb anti-V5 (αV5), and mouse mAb anti-gE (αgE). Numbers to the right of the blots are molecular weight standards (kDa). (TIF) [file ppat.1008961.s003.tif]
