# Supplementary material for: The N-terminus of varicella-zoster virus glycoprotein B has a functional role in fusion
Source: PLoS Pathog. 2021 Jan 7;17(1):e1008961. doi: 10.1371/journal.ppat.1008961 (PMC7817050; doi:10.1371/journal.ppat.1008961)
Supplement: S1 Table — (DOCX) [file ppat.1008961.s004.docx]

**S1 Table.** Cryo-EM data collection parameters for the native full-length VZV gB (EMDB 22629), and the gB-93k (EMDB 22519) and gB-SG2 (EMDB 22520) complexes.

| **Parameter** | **Structure** | | |
| --- | --- | --- | --- |
|  | **Native VZV gB** | **gB-93k Fab** | **gB-SG2 Fab** |
| **Data collection and processing** |  |  |  |
| Microscope | Titan Krios (FEI) | Tecnai F20 (FEI) | Tecnai F20 (FEI) |
| Magnification (1000X) | 130 | 29 | 29 |
| Voltage (kV) | 300 | 200 | 200 |
| Energy filter slit width (eV) | 20 | N/A | N/A |
| Detector | Gatan K2 Summit | Gatan K2 Summit | Gatan K2 Summit |
| Defocus range (μm) | 1.5-2.0 | 1.8-3 | 1.8-3 |
| Pixel size (Å) | 1.06 | 1.283 | 1.283 |
| Exposure time (s) | 12 | 15 | 15 |
| Frames | 60 | 75 | 75 |
| Electron dose (e^-^/s) | 7.5 | 7.8 | 7.8 |
| Electron exposure rate (e^-^/Å^2^/s) | 1.335 | 4.2 | 4.2 |
| Total electron exposure (e^-^/Å^2^) | 16.02 | 63 | 63 |
| Symmetry imposed | 3 | 3 | 3 |
| Micrographs collected (no.) | 10,241 | 649 | 795 |
| Final particle images (no.) | 349,207 | 22,976 | 25,670 |
| Map resolution (Å) | 3.9 | 7.3 | 9.0 |
| FSC threshold | 0.143 | 0.143 | 0.143 |
|  |  |  |  |
| **Refinement** |  |  |  |
| Initial model used (PDB code) | 6VLK^A^ | N/A | N/A |
| Model resolution (Å) | 3.9 | N/A | N/A |
| FSC threshold | 0.143 | N/A | N/A |
| Model composition |  | N/A | N/A |
| Chains | 6 | N/A | N/A |
| Non-hydrogen atoms | 14,409 | N/A | N/A |
| Protein residues | 1,752 | N/A | N/A |
|  |  |  |  |
| **Validation** |  |  |  |
| B factors (Å^2^) |  | N/A | N/A |
| Protein (min/max/mean) | 42/164/72 | N/A | N/A |
| Protein (min/max/mean) | 59/151/93 | N/A | N/A |
| R.M.S. deviations |  | N/A | N/A |
| Bond lengths (Å) | 0.006 | N/A | N/A |
| Bond angles (°) | 0.851 | N/A | N/A |
| Validation |  | N/A | N/A |
| MolProbity score | 1.7 | N/A | N/A |
| Clashscore | 15 | N/A | N/A |
| Poor rotamers (%) | 0.4 | N/A | N/A |
| Ramachandran plot |  | N/A | N/A |
| Favored (%) | 98 | N/A | N/A |
| Allowed (%) | 2 | N/A | N/A |
| Disallowed (%) | 0 | N/A | N/A |

^A^ X-ray crystallography data for VZV gB.
