# Supplementary material for: The N-terminus of varicella-zoster virus glycoprotein B has a functional role in fusion
Source: PLoS Pathog. 2021 Jan 7;17(1):e1008961. doi: 10.1371/journal.ppat.1008961 (PMC7817050; doi:10.1371/journal.ppat.1008961)
Supplement: S2 Table — (DOCX) [file ppat.1008961.s005.docx]

**S2 Table.** X-ray data collection and structure refinement for VZV gB (PDB 6VLK).

| **Parameter** |  |
| --- | --- |
| **X-ray Data** |  |
| Beamline | APS 17-ID |
| Space group | R32 |
| Cell dimensions: a, b, c (Å), α, β, γ (°) | 118.318, 118.318, 749.026  90, 90, 120 |
| Resolution (Å) | 101.52-2.45 (2.59-2.45)^A^ |
| R_merge_ | 0.073 (0.516)^A^ |
| I/σ | 19.8(3.7)^A^ |
| Completeness (%) | 100.0(100.0)^A^ |
| Redundancy | 9.8(10.0)^A^ |
|  |  |
| **Refinement** |  |
| Model Resolution(Å) | 50.2-2.4 |
| Number of reflections | 74911 |
| *R_work_/R_free_*^B^ | 0.1845/0.23 |
| Model Composition |  |
| Protein | 9381 |
| Glycan | 214 |
| Water | 171 |
|  |  |
| **Validation** |  |
| B factors (Å^2^) |  |
| Protein (min/max/mean) | 7/155/44 |
| Glycan (min/max/mean) | 24/145/43 |
| R.M.S. deviations |  |
| Bond lengths (Å) | 0.01 |
| Bond angles (°) | 1.14 |
| Validation |  |
| MolProbity score | 1.69 |
| Clashscore | 3 |
| Poor rotamers (%) | 4.6 |
| Ramachandran plot |  |
| Favored (%) | 98 |
| Allowed (%) | 2 |
| Disallowed (%) | 0 |
|  |  |

^A^Outer-shell values are given in parentheses.

^B^The *Rfree* test set was composed of 5% randomly chosen reflections.
