# Supplementary material for: The N-terminus of varicella-zoster virus glycoprotein B has a functional role in fusion
Source: PLoS Pathog. 2021 Jan 7;17(1):e1008961. doi: 10.1371/journal.ppat.1008961 (PMC7817050; doi:10.1371/journal.ppat.1008961)
Supplement: S3 Table — (DOCX) [file ppat.1008961.s006.docx]

**S3 Table.** Amino acid residues and color code for each domain in VZV gB.

| **Residues** | **Domain** | **Color** |
| --- | --- | --- |
| 115-136 | IV | Orange |
| 137-147 | Linker: II-IV | Hot Pink |
| 148-159 | II | Green |
| 160-368 | I | Cyan |
| 369-464 | II | Green |
| 465-502 | N/A | N/A |
| 503-510 | Linker: II-III | Hot Pink |
| 511-569 | III | Yellow |
| 570-681 | IV | Orange |
| 682-736 | V | Red |

N/A – Not applicable.
