# Supplementary material for: The N-terminus of varicella-zoster virus glycoprotein B has a functional role in fusion
Source: PLoS Pathog. 2021 Jan 7;17(1):e1008961. doi: 10.1371/journal.ppat.1008961 (PMC7817050; doi:10.1371/journal.ppat.1008961)
Supplement: S4 Table — (DOCX) [file ppat.1008961.s007.docx]

**S4 Table.** N-linked glycosylation sites identified in VZV and herpesvirus gB orthologues.

| **Domain** | **VZV** | | **HSV** | **PRV** | **HCMV** | **EBV** |
| --- | --- | --- | --- | --- | --- | --- |
|  | **Predicted** | **Observed** |  |  |  |  |
| DII/IV linker | N147 | N147^B/C^ | N141 | N154 | X | N76 |
| DI | N257 | N257^A/B/C^ | X – N255 | N264 | X | X |
| DII | N435 | N435^B/C^ | N430 | N444 | N409 | X |
| DII no structure | N479 | - | - | - | - | - |
| DII/III linker | N503 | N503^C^ | - | - | - | - |
| DIII | N557 | - | - | - | - | - |
| DIV | N620 | N620^A/B^ | - | N636 | N586 | N563 |
| DV | N686 | N686^A/B/C^ | - | - | - | - |
|  |  |  |  |  |  |  |

^A^ Identified in the gB ectodomain X-ray crystal structure

^B^ Density observed in Cryo-EM map

^C^ Identified by Orbitrap Mass Spectrometry
