# Supplementary material for: The N-terminus of varicella-zoster virus glycoprotein B has a functional role in fusion
Source: PLoS Pathog. 2021 Jan 7;17(1):e1008961. doi: 10.1371/journal.ppat.1008961 (PMC7817050; doi:10.1371/journal.ppat.1008961)
Supplement: S5 Table — (DOCX) [file ppat.1008961.s008.docx]

**S5 Table.** Conserved cysteine bonds in herpesvirus gB orthologues.

| **Domain Location** | **Cysteine Residue Locations in gB** | | | | |
| --- | --- | --- | --- | --- | --- |
|  | **VZV** | **HSV** | **PRV** | **HCMV** | **EBV** |
| DIV | 122-584 | 116-573 | 129-603 | 94-551 | 51-528 |
| DII/IV linker to DIII | 139-540 | 133-529 | 146-559 | 111-507 | 68-484 |
| DI | 213-277 | 207-271 | 220-284 | 185-250 | 141-206 |
| DII | 369-417 | 364-412 | 377-426 | 344-391 | 295-342 |
| DIV | 608-645 | 596-633 | 625-661 | 574-611 | 551-588 |
|  |  |  |  |  |  |
