# Supplementary material for: The N-terminus of varicella-zoster virus glycoprotein B has a functional role in fusion
Source: PLoS Pathog. 2021 Jan 7;17(1):e1008961. doi: 10.1371/journal.ppat.1008961 (PMC7817050; doi:10.1371/journal.ppat.1008961)
Supplement: S6 Table — (DOCX) [file ppat.1008961.s009.docx]

**S6 Table.** Amino acid residues from X-ray crystallography data for the herpesvirus gB orthologues used to calculate amino acid identities and RMSD.

| **Domain** | **Amino acid residues within each domain** | | | |
| --- | --- | --- | --- | --- |
|  | **HSV (2GUM^A^)** | **PRV (6ESC^A^)** | **HCMV (5CXF^A^)** | **EBV (3FVC^A^)** |
| Complete Structure | 111-724 | 122-750 | 87-695 | 44-679 |
| I | 154-363 | 167-376 | 133-343 | 89-294 |
| II | 142-153/364-458 | 155-166/377-473 | 121-132/344-438 | 77-88/295-389 |
| III | 492-558 | 522-589 | 476-536 | 448-513 |
| IV | 111-130/559-669 | 122-143/590-695 | 87-108/537-647 | 44-65/514-624 |
| V | 670-724 | 696-750 | 648-695 | 625-679 |
|  |  |  |  |  |

^A^ Protein Data Bank accession number
