# Supplementary material for: The N-terminus of varicella-zoster virus glycoprotein B has a functional role in fusion
Source: PLoS Pathog. 2021 Jan 7;17(1):e1008961. doi: 10.1371/journal.ppat.1008961 (PMC7817050; doi:10.1371/journal.ppat.1008961)
Supplement: S7 Table — (DOCX) [file ppat.1008961.s010.docx]

**S7 Table.** Amino acid identities of VZV gB derived from structure-based alignments with herpesvirus gB orthologues.

| **Domain** | **Amino acid identity (similarity) [%] compared to VZV** | | | |
| --- | --- | --- | --- | --- |
|  | **HSV** | **PRV** | **HCMV** | **EBV** |
| Complete Structure | 50 (71) | 61 (77) | 30 (51) | 29 (50) |
| I | 48 (64) | 61 (75) | 28 (44) | 27 (43) |
| II | 46 (74) | 50 (70) | 32 (53) | 22 (46) |
| III | 54 (73) | 57 (75) | 33 (61) | 26 (55) |
| IV | 50 (70) | 58 (73) | 29 (50) | 40 (56) |
| V | 63 (89) | 78 (92) | 32 (57) | 27 (55) |
|  |  |  |  |  |
