# Supplementary material for: The N-terminus of varicella-zoster virus glycoprotein B has a functional role in fusion
Source: PLoS Pathog. 2021 Jan 7;17(1):e1008961. doi: 10.1371/journal.ppat.1008961 (PMC7817050; doi:10.1371/journal.ppat.1008961)
Supplement: S8 Table — (DOCX) [file ppat.1008961.s011.docx]

**S8 Table.** RMSD of VZV gB derived from structure-based alignments with herpesvirus gB orthologues.

| **Domain** | **RMSD (Å)^A^ compared to VZV gB** | | | | | | | | | | |
| --- | --- | --- | --- | --- | --- | --- | --- | --- | --- | --- | --- |
|  | **HSV** | | | **PRV** | | | **HCMV** | | | **EBV** | |
| Complete Structure | 0.987 (473) | 1.643 (574) | 0.855 (337) | | 2.880 (578) | 1.268 (136) | | 7.154 (558) | 1.154 (226) | | 10.025 (546) |
| I | 0.635 (195) | 1.391 (203) | 0.593 (198) | | 1.008 (209) | 1.054 (108) | | 3.657 (205) | 1.084 (156) | | 2.536 (204) |
| II | 0.635 (95) | 15.115 (107) | 0.779 (91) | | 15.141 (107) | 0.863 (80) | | 13.179 (101) | 0.827 (70) | | 11.753 (99) |
| III | 0.548 (67) | 0.548 (67) | 0.709 (60) | | 1.687 (68) | 0.847 (61) | | 0.847 (61) | 0.936 (60) | | 1.641 (66) |
| IV | 1.022 (96) | 6.133 (125) | 0.892 (94) | | 6.904 (123) | 0.978 (80) | | 4.724 (116) | 1.017 (80) | | 8.040 (110) |
| V | 0.649 (55) | 0.649 (55) | 0.531 (55) | | 0.531 (55) | 1.149 (29) | | 4.260 (48) | 1.157 (38) | | 3.032 (51) |
|  |  | | |  | | |  | | |  | |

^A^ Root mean squared deviation (RMSD) calculated using Matchmaker (Chimera). The two values correspond to the trimmed amino acids versus all of the amino acids in the alignment used by Matchmaker.
