# Supplementary material for: The N-terminus of varicella-zoster virus glycoprotein B has a functional role in fusion
Source: PLoS Pathog. 2021 Jan 7;17(1):e1008961. doi: 10.1371/journal.ppat.1008961 (PMC7817050; doi:10.1371/journal.ppat.1008961)
Supplement: S9 Table — (DOCX) [file ppat.1008961.s012.docx]

**S9 Table.** Key reagents and resources.

| **REAGENT or RESOURCE** | **SOURCE** | **IDENTIFIER** |
| --- | --- | --- |
| **Antibodies** | | |
| Human mAb 93k | This paper |  |
| Mouse mAb SG2-2E6 | GeneTex | GTX38718 |
| Rabbit polyclonal antibody 746-868 | [1] |  |
| Mouse mAb IE62 | EMD Millipore | MAB8616 |
| Mouse mAb gE | EMD Millipore | MAB8612 |
| VZV mouse mixed mAb | Meridian Life Sciences | C05108MA |
| Mouse anti-V5 tag | Bio-Rad | MCA1360 |
|  |  |  |
| Biotinylated goat anti-mouse IgG (H+L) | Vector Labs. Inc | BA-9200 |
| Donkey anti-mouse Alexa Fluor 555 | Life Technologies | A31570 |
| Donkey anti-human Alexa Fluor 488 | Life Technologies | A11013 |
| ECL Sheep anti-mouse IgG, Horseradish peroxidase linked whole antibody | GE Healthcare UK Ltd | NA931V |
| ECL Donkey anti-rabbit IgG, Horseradish peroxidase linked whole antibody | GE Healthcare UK Ltd | NA934V |
| ECL Sheep anti-human IgG, Horseradish peroxidase linked whole antibody | GE Healthcare UK Ltd | NA933V |
|  |  |  |
| **Bacterial and Virus Strains** | | |
| pOka BAC derived (pPOKA-DX) | [2] |  |
| GS1783 | [3] |  |
| pOka-TK-GFP | [4] |  |
| pOka-TK-GFP gB-STEVV5 | This paper |  |
| pOka-TK-GFP gB-TEVV5 | This paper |  |
| pOka-TK-GFP gB-TEVV5 gB[S110A] | This paper |  |
| pOka-TK-GFP gB-TEVV5 gB[Q111A] | This paper |  |
| pOka-TK-GFP gB-TEVV5 gB[D112A] | This paper |  |
| pOka-TK-GFP gB-TEVV5 gB[^109^AAAA^112^] | This paper |  |
|  |  |  |
| **Chemicals, Peptides, and Recombinant Proteins** | | |
| Anti-V5 Agarose Affinity Gel | Sigma | A7345-1ML |
| Bovine Serum Albumin (IgG Free, Protease Free) | Jackson ImmunoResearch | 001-000-162 |
| Minimal essential medium | Corning cellgro | 10-010-CV |
| F-12K nutrient mixture Kaighn’s modification | Invitrogen | 21127-022 |
| Optimem +Glutamax | Gibco | 51985-034 |
| Fetal bovine serum | Gibco | 26140-079 |
| Penicillin /Streptomycin | Gibco | 15140-122 |
| Amphotericin B | Corning cellgro | 30-003-CF |
| Nonessential amino acids | Corning cellgro | 25-025-CI |
| Puromycin | Invitrogen | A11138-03 |
| BstZ171 | New England BioLabs Inc. | R3594 |
| NaeI | New England BioLabs Inc. | R0190L |
| HindIII | New England BioLabs Inc. | R0104L |
| AgeI | New England BioLabs Inc. | R0552L |
| SpeI | New England BioLabs Inc. | R0133L |
| NotI | New England BioLabs Inc. | R0189L |
| KpnI | New England BioLabs Inc. | R0142L |
| XmaI | New England BioLabs Inc. | R0180L |
| NdeI | New England BioLabs Inc. | R0111L |
| AccuPrime *Pfx* DNA Polymerase | Invitrogen | 12344024 |
| KOD Extreme Hot Start DNA Polymerase | EMD Millipore | 71975-3 |
| Lipofectamine 2000 | Invitrogen | 11668-019 |
| Ampicillin | Sigma | A9518-100G |
| Kanamycin | Sigma | K4378 |
| LB (Miller’s) Agar | Growcells | MBPE-3060 |
| LB (Miller’s) Broth | Growcells | MBPE-1050 |
| PBS (Phosphate buffered saline) | Corning cellgro | 21-040-CV |
| DPBS (Dulbecco’s phosphate buffered saline) | Corning cellgro | 21-030-CV |
| Tris Base | Fisher Scientific | BP152-5 |
| Sodium chloride | Fisher Scientific | S271-10 |
| Potassium chloride | Fisher Scientific | BP366-500 |
| Magnesium chloride | Fisher Scientific | M33-500 |
| Sodium deoxycholate | ICN Biomedical Inc. | 804312 |
| IGEPAL CA-630 | Sigma | I3021-100ML |
| Triton X-100 | Sigma | T-9284 |
| Tobacco etch virus protease | In house |  |
| Tris buffered saline (TBS; pH7.4) | Scy Tek | TBS500 |
| Amphipol 8-35 | Anatrace | A835 |
| Lauroylsarcosine | Sigma | L9150 |
| Bio-Beads SM-2 | Bio-Rad | 152-3920 |
| Phosphotungstic acid | Ted Pella | 19402 |
| EDTA | Sigma | E9884 |
| Sucrose | Sigma | S7903-1KG |
| L(+)-glutamic acid monosodium salt monohydrate | Sigma | G1626-100G |
| Paraformaldehyde (4%) in PBS | Boston Bioproducts | K06J101 |
| BD Cytofix/Cytoperm Plus | BD Biosciences | 555028 |
| Hoechst 33342 | ThermoFisher Scientific | H-3570 |
| Native PAGE Sample Buffer | Novex | BN20032 |
| Native PAGE running buffer 20X | Novex | BN2001 |
| Native PAGE 20X cathode buffer additive | Novex | BN2002 |
| Native PAGE 3-12% Bis Tris Gel | Novex | BN2011BX10 |
| Laemmli Sample Buffer 2X | Bio-Rad | 161-0737 |
| 2-mercaptoethanol | Sigma | M7522-100ML |
| Mini Protean TGX Gels 4-20% | Bio-Rad | 456-1094 |
| NativeMark Protein Std. | Invitrogen | 57030 |
| Novex Sharp Pre-Stained Protein Standards | Invitrogen | 57318 |
| Dimethyl pimelimidate dihydrochloride | Sigma | D8388 |
| Protein A Plus UltraLink Resin | Thermo Scientific | 53142 |
| Fluoromount-G | SouthernBiotech | 0100-01 |
| Boric acid | Sigma | B-0252 |
| Ethanolamine | Sigma | 398136-500ML |
| Ethane | Airgas | ET R80 |
| L-(+)-Arabinose | Sigma | A3256-100G |
| Agarose LE | AccuFlow | EK2808 |
| Membrane permeable coelenterazine-H | Nanolight Technology | 3012-10 |
| Fast Red TR Salt hemi (zinc chloride) | Sigma | 368881-25G |
| Naphthol AS-MX phosphate | Sigma | N4875-500MG |
| Alkaline phosphatase-conjugated Streptavidin | Jackson ImmunoResearch | 016-050-084 |
|  |  |  |
| **Experimental Models: Cell Lines** | | |
| MeWo | ATCC | HTB-65 |
| CHO DSP1 | [5] |  |
| Mel-DSP2 | [5] |  |
|  |  |  |
| **Oligonucleotides (All sequences are 5’ to 3’)** | | |
| **S/TEVV5** |  |  |
| gB-AgeI  CTTTTTTGCGTACCGGTACGTGC | [6] | Elim Bio |
| gB931  [PHOS]CACCCCCGTTACATTCTCGGTGCG | [6] | Elim Bio |
| gB-V5  [PHOS]GGTAAGCCTATCCCTAACCCTCTCCTCGGTCTCGATTCTACGTAAATAGCCAGGGGGTTT | [6] | Elim Bio |
| M13R  CACCCCCGTTACATTCTCGGTGCG | Invitrogen |  |
| gB-Cterm-S-tag  [PHOS]GCTGTCCATGTGCTGGCGTTCGAATTTAGCAGCAGCGGTTTCTTTCACCCCCGTTACATTCTCGG | [6] | Elim Bio |
| gB-link_TEV_link  [PHOS]*GGCGGCGGGGGCGGG*GAGAATCTTTATTTTCAGG*GCGGGGGCG*GGGGTAAGCCTATCCCTAACCC | [6] | Elim Bio |
| ∆S-tag-sense  [PHOS]GGCGGCGGGGGCGGGGAGATTC | [6] | Elim Bio |
| ∆S-tag-antisense [PHOS]CACCCCCGTTACATTCTCGGTG | [6] | Elim Bio |
| [31]F56625-56645  AGGTATAGGCAGTTCCCACGG | [7] | Elim Bio |
| [31]R59697-59717  TTTCATTGAGACTTGAAGCGC | [7] | Elim Bio |
|  |  |  |
| **gB KSQD 109/110/111/112** |  |  |
| pCAGGs-gB-NotI-sense  AAAGAATTCGCGGCCGCTGACCG | This paper | Elim Bio |
| gB-93k-sense  [PHOS]CAGGACGCCGAAACAAAACCCACGTTTTACG | This paper | Elim Bio |
| K109R-sense  [PHOS]CGGTCCCAGGACGCCGAAACAAAACC |  |  |
| Q111A-sense  [PHOS]GCGGACGCCGAAACAAAACCCACGTTTTACG | This paper | Elim Bio |
| K112A-sense  [PHOS]CAGGCTGCCGAAACAAAACCCACGTTTTACG | This paper | Elim Bio |
| Q111A-K112A sense  [PHOS]GCGGCTGCCGAAACAAAACCCACGTTTTACG | This paper | Elim Bio |
| pCAGGs-gB-KpnI-antisense  TCTCTGAAACGGGAATTGGTACC | This paper | Elim Bio |
| gB-93k-antisense  GGACTTGTGTATAGCTTCTCTGATTTCATCACC | This paper | Elim Bio |
| K109A-antisense  [PHOS]GGACGCGTGTATAGCTTCTCTGATTTCATCACC | This paper | Elim Bio |
| H108-antisense  [PHOS]GTGTATAGCTTCTCTGATTTCATCAC | This paper | Elim Bio |
| S110A-antisense  [PHOS]AGCCTTGTGTATAGCTTCTCTGATTTCATCACC | This paper | Elim Bio |
| K109A-S110A-antisense  [PHOS]AGCCGCGTGTATAGCTTCTCTGATTTCATCACC | This paper | Elim Bio |
|  | This paper | Elim Bio |
|  |  |  |
| **Recombinant DNA** | | |
|  |  |  |
| pCAGGs-VZVgB | [8] |  |
| pME18s | [8] |  |
| pME18s-gH[TL] | [8] |  |
| pME18s-gH[V5] | [4] |  |
| pCDNA3.1(+) | Invitrogen |  |
| pCDNA3.1-gL | [9] |  |
| pBud-gE/gI | [10] |  |
|  |  |  |
| pCAGGs-gB[K109A] | This paper |  |
| pCAGGs-gB[K109R] | This paper |  |
| pCAGGs-gB[S110A] | This paper |  |
| pCAGGs-gB[Q111A] | This paper |  |
| pCAGGs-gB[D112A] | This paper |  |
|  |  |  |
| pPOKA-TK-GFP gB-STEVV5 | This paper |  |
| pPOKA-TK-GFP gB-TEVV5 | [6] |  |
| pPOKA-TK-GFP gB-TEVV5 gB[K109A] | This paper |  |
| pPOKA-TK-GFP gB-TEVV5 gB[K109R] | This paper |  |
| pPOKA-TK-GFP gB-TEVV5 gB[S110A] | This paper |  |
| pPOKA-TK-GFP gB-TEVV5 gB[Q111A] | This paper |  |
| pPOKA-TK-GFP gB-TEVV5 gB[D112A] | This paper |  |
|  |  |  |
| **Software and Algorithms** | | |
| SerialEM | [11] | http://bio3d.colorado.edu/SerialEM/ |
| Relion v3.0 | [12, 13] | https://bitbucket.org/scheres/relion-3.0_beta/src/master/ |
| ResMap v1.95 | [14] | https://sourceforge.net/projects/resmap-latest/ |
| UCSF Chimera v1.13.1 | [15] | http://www.cgl.ucsf.edu/chimera/ |
| ModelZ | [16] | https://cryoem.slac.stanford.edu/ncmi/resources/software/modelz |
| Phenix | [17] | https://www.phenix-online.org/ |
| WinCoot | [18] | http://bernhardcl.github.io/coot/ |
| RaptorX | [19] | http://raptorx.uchicago.edu/ |
| SWISS-MODEL | [20] | https://swissmodel.expasy.org/ |
| CHARMM-GUI | [21] | http://www.charmm-gui.org/ |
| Segger v1.9.5 | [22] | https://cryoem.slac.stanford.edu/ncmi/resources/software/segger |
| CellQuest Pro | BD |  |
| FlowJo | TreeStar |  |
| Prism 8 | GraphPad Software, Inc. |  |
| Staden | [23] | http://staden.sourceforge.net |
| GeneDoc v2.7 | Nicholas and Nicholas, 1997 | https://genedoc.software.informer.com/download/ |
| FiJi (ImageJ 1.52i) | NIH, USA | http://imagej.nih.gov/ij |
| Illustrator CS6 | Adobe |  |
| Photoshop CS6 | Adobe |  |
| AxioVision | Zeiss |  |
|  |  |  |
| **Other** | | |
| NativePAGE 2-12% Bis-Tris Gel | Invitrogen | BN2011BX10 |
| Mini-PROTEAN TGX Gels | Bio-Rad | 456-1094 |
| Superose-6 Increase 3.2x300mm | Sigma | GE29-0915-98 |
| Ultrathin carbon film on lacey carbon support 400M Cu | Ted Pella | 01824 |
| Quantifoil R 1.2/1.3 Au 300 mesh grids | Quantifoil |  |
| Tecnai F20 | FEI |  |
| Leica EM GP | Leica |  |
| Titan Krios | FEI |  |
| Pierce Fab Preparation Kit | Thermo Scientific | 44985 |
| Amicon Ultra-4 Centrifugal Filter Units 100kDa | Millipore | UFC810024 |
| Amicon Ultra-4 Centrifugal Filter Units 10kDa | Millipore | UFC801024 |
| QIAquick Gel Extraction Kit | Qiagen | 28706 |
| QIAquick Nucleotide Removal Kit | Qiagen | 28304 |
| QIAprep Spin Miniprep Kit | Qiagen | 27106 |
| QIAGEN Large-Construct Kit | Qiagen | 12462 |
| Immobilon-P | Merck Millipore Ltd. | IPVH00010 |
| Optical bottom 96-well black sided culture plates | Thermo Scientific | 165305 |
| FACSCalibur flow cytometer | Becton Dickenson |  |
| Synergy H1 Multi-mode Reader | Biotek |  |
| Nunclon Delta Surface 12-well plates | Thermo Scientific | 150628 |
| Cell Culture 6-well plates | Corning | 3506 |
| Microscope cover glass 18mm No. 1 | Fisher Scientific | 12-545-100 |
| Orbitrap Fusion mass Spectrometer | Thermo Scientific |  |
| Acquity M-Class liquid chromatograph | Waters Corporation |  |
| 1.8 micron C18 stationary phase beads | Dr. Maisch | http://www.dr-maisch.com |
|  |  |  |

**References.**

1. Oliver SL, Sommer M, Zerboni L, Rajamani J, Grose C, Arvin AM. Mutagenesis of varicella-zoster virus glycoprotein B: putative fusion loop residues are essential for viral replication, and the furin cleavage motif contributes to pathogenesis in skin tissue in vivo. J Virol. 2009;83(15):7495-506. doi: 10.1128/JVI.00400-09. PubMed PMID: 19474103; PubMed Central PMCID: PMCPMC2708640.

2. Tischer BK, Kaufer BB, Sommer M, Wussow F, Arvin AM, Osterrieder N. A self-excisable infectious bacterial artificial chromosome clone of varicella-zoster virus allows analysis of the essential tegument protein encoded by ORF9. J Virol. 2007;81(23):13200-8. doi: 10.1128/JVI.01148-07. PubMed PMID: 17913822; PubMed Central PMCID: PMCPMC2169085.

3. Tischer BK, Smith GA, Osterrieder N. En passant mutagenesis: a two step markerless red recombination system. Methods Mol Biol. 2010;634:421-30. doi: 10.1007/978-1-60761-652-8_30. PubMed PMID: 20677001.

4. Yang E, Arvin AM, Oliver SL. The cytoplasmic domain of varicella-zoster virus glycoprotein H regulates syncytia formation and skin pathogenesis. PLoS Pathog. 2014;10(5):e1004173. doi: 10.1371/journal.ppat.1004173. PubMed PMID: 24874654; PubMed Central PMCID: PMCPMC4038623.

5. Yang E, Arvin AM, Oliver SL. Role for the alphaV Integrin Subunit in Varicella-Zoster Virus-Mediated Fusion and Infection. J Virol. 2016;90(16):7567-78. doi: 10.1128/JVI.00792-16. PubMed PMID: 27279620; PubMed Central PMCID: PMCPMC4984616.

6. Oliver SL, Xing Y, Chen DH, Roh SH, Pintilie GD, Bushnell DA, et al. A glycoprotein B-neutralizing antibody structure at 2.8 A uncovers a critical domain for herpesvirus fusion initiation. Nat Commun. 2020;11(1):4141. doi: 10.1038/s41467-020-17911-0. PubMed PMID: 32811830; PubMed Central PMCID: PMCPMC7435202.

7. Oliver SL, Brady JJ, Sommer MH, Reichelt M, Sung P, Blau HM, et al. An immunoreceptor tyrosine-based inhibition motif in varicella-zoster virus glycoprotein B regulates cell fusion and skin pathogenesis. Proc Natl Acad Sci U S A. 2013;110(5):1911-6. doi: 10.1073/pnas.1216985110. PubMed PMID: 23322733; PubMed Central PMCID: PMCPMC3562845.

8. Suenaga T, Satoh T, Somboonthum P, Kawaguchi Y, Mori Y, Arase H. Myelin-associated glycoprotein mediates membrane fusion and entry of neurotropic herpesviruses. Proc Natl Acad Sci U S A. 2010;107(2):866-71. doi: 10.1073/pnas.0913351107. PubMed PMID: 20080767; PubMed Central PMCID: PMCPMC2818916.

9. Vleck SE, Oliver SL, Brady JJ, Blau HM, Rajamani J, Sommer MH, et al. Structure-function analysis of varicella-zoster virus glycoprotein H identifies domain-specific roles for fusion and skin tropism. Proc Natl Acad Sci U S A. 2011;108(45):18412-7. doi: 10.1073/pnas.1111333108. PubMed PMID: 22025718; PubMed Central PMCID: PMCPMC3215059.

10. Oliver SL, Sommer MH, Reichelt M, Rajamani J, Vlaycheva-Beisheim L, Stamatis S, et al. Mutagenesis of varicella-zoster virus glycoprotein I (gI) identifies a cysteine residue critical for gE/gI heterodimer formation, gI structure, and virulence in skin cells. J Virol. 2011;85(9):4095-110. doi: 10.1128/JVI.02596-10. PubMed PMID: 21345964; PubMed Central PMCID: PMCPMC3126246.

11. Mastronarde DN. Automated electron microscope tomography using robust prediction of specimen movements. J Struct Biol. 2005;152(1):36-51. doi: 10.1016/j.jsb.2005.07.007. PubMed PMID: 16182563.

12. Zivanov J, Nakane T, Forsberg BO, Kimanius D, Hagen WJ, Lindahl E, et al. New tools for automated high-resolution cryo-EM structure determination in RELION-3. Elife. 2018;7. doi: 10.7554/eLife.42166. PubMed PMID: 30412051; PubMed Central PMCID: PMCPMC6250425.

13. Scheres SH. RELION: implementation of a Bayesian approach to cryo-EM structure determination. J Struct Biol. 2012;180(3):519-30. doi: 10.1016/j.jsb.2012.09.006. PubMed PMID: 23000701; PubMed Central PMCID: PMCPMC3690530.

14. Kucukelbir A, Sigworth FJ, Tagare HD. Quantifying the local resolution of cryo-EM density maps. Nat Methods. 2014;11(1):63-5. doi: 10.1038/nmeth.2727. PubMed PMID: 24213166; PubMed Central PMCID: PMCPMC3903095.

15. Pettersen EF, Goddard TD, Huang CC, Couch GS, Greenblatt DM, Meng EC, et al. UCSF Chimera--a visualization system for exploratory research and analysis. J Comput Chem. 2004;25(13):1605-12. doi: 10.1002/jcc.20084. PubMed PMID: 15264254.

16. Pintilie G, Chiu W. Assessment of Structural Features in Cryo-EM Density Maps using SSE and Side Chain Z-Scores. J Struct Biol. 2018. doi: 10.1016/j.jsb.2018.08.015. PubMed PMID: 30144506.

17. Adams PD, Afonine PV, Bunkoczi G, Chen VB, Davis IW, Echols N, et al. PHENIX: a comprehensive Python-based system for macromolecular structure solution. Acta Crystallogr D Biol Crystallogr. 2010;66(Pt 2):213-21. doi: 10.1107/S0907444909052925. PubMed PMID: 20124702; PubMed Central PMCID: PMCPMC2815670.

18. Emsley P, Lohkamp B, Scott WG, Cowtan K. Features and development of Coot. Acta Crystallogr D Biol Crystallogr. 2010;66(Pt 4):486-501. doi: 10.1107/S0907444910007493. PubMed PMID: 20383002; PubMed Central PMCID: PMCPMC2852313.

19. Xu J. Distance-based protein folding powered by deep learning. Proc Natl Acad Sci U S A. 2019;116(34):16856-65. doi: 10.1073/pnas.1821309116. PubMed PMID: 31399549; PubMed Central PMCID: PMCPMC6708335.

20. Waterhouse A, Bertoni M, Bienert S, Studer G, Tauriello G, Gumienny R, et al. SWISS-MODEL: homology modelling of protein structures and complexes. Nucleic Acids Res. 2018;46(W1):W296-W303. doi: 10.1093/nar/gky427. PubMed PMID: 29788355; PubMed Central PMCID: PMCPMC6030848.

21. Lee J, Patel DS, Stahle J, Park SJ, Kern NR, Kim S, et al. CHARMM-GUI Membrane Builder for Complex Biological Membrane Simulations with Glycolipids and Lipoglycans. J Chem Theory Comput. 2019;15(1):775-86. doi: 10.1021/acs.jctc.8b01066. PubMed PMID: 30525595.

22. Pintilie G, Chen DH, Haase-Pettingell CA, King JA, Chiu W. Resolution and Probabilistic Models of Components in CryoEM Maps of Mature P22 Bacteriophage. Biophys J. 2016;110(4):827-39. doi: 10.1016/j.bpj.2015.11.3522. PubMed PMID: 26743049; PubMed Central PMCID: PMCPMC4775875.

23. Staden R. The Staden sequence analysis package. Mol Biotechnol. 1996;5(3):233-41. PubMed PMID: 8837029.
