# Supplementary material for: The N-terminus of varicella-zoster virus glycoprotein B has a functional role in fusion
Source: PLoS Pathog. 2021 Jan 7;17(1):e1008961. doi: 10.1371/journal.ppat.1008961 (PMC7817050; doi:10.1371/journal.ppat.1008961)
Supplement: S2 Validation Report — (PDF) [file ppat.1008961.s020.pdf]

Full wwPDB EM Map Validation Report (i)

Aug 31, 2020 – 11:57 AM EDT

EMDB ID	:	EMD-22520
Title	:	The N-terminus of varicella-zoster virus glycoprotein B has a functional role in fusion
Deposited on	:	2020-08-27
Resolution	:	9.00 Å(reported)

This is a Full wwPDB EM Map Validation Report.

This report is produced by the wwPDB biocuration pipeline after annotation of the structure.

We welcome your comments at *validation@mail.wwpdb.org*
A user guide is available at
https://www.wwpdb.org/validation/2017/EMMapValidationReportHelp
with specific help available everywhere you see the (i) symbol.

CONFIDENTIAL VALIDATION REPORT

The following versions of software and data (see references (i)) were used in the production of this report:

EMDB validation analysis	:	0.0.0.dev33
Validation Pipeline (wwPDB-VP)	:	2.13.1

1 Experimental information (i)

Property	Value	Source
EM reconstruction method	SINGLE PARTICLE	Depositor
Imposed symmetry	POINT, Not provided	Depositor
Number of particles used	59610	Depositor
Resolution determination method	FSC 0.143 CUT-OFF	Depositor
CTF correction method	NONE	Depositor
Microscope	FEI TITAN KRIOS	Depositor
Voltage (kV)	300	Depositor
Electron dose $(e^-/\text{Å}^2)$	7.8	Depositor
Minimum defocus (nm)	Not provided	Depositor
Maximum defocus (nm)	Not provided	Depositor
Magnification	Not provided	Depositor
Image detector	GATAN K2 SUMMIT (4k x 4k)	Depositor
Maximum map value	11.432	Depositor
Minimum map value	-7.053	Depositor
Average map value	0.000	Depositor
Map value standard deviation	0.273	Depositor
Recommended contour level	2.16	Depositor
Map size (Å)	513.20026, 513.20026, 513.20026	Depositor
Map dimensions	200, 200, 200	Depositor
Map angles (°)	90.0, 90.0, 90.0	Depositor
Pixel spacing (Å)	2.5660012, 2.5660012, 2.5660012	Depositor

CONFIDENTIAL VALIDATION REPORT

2 Map visualisation (i)

This section contains visualisations of the EMDB entry EMD-22520. These are intended to permit visual inspection of the internal detail of the map and identification of artifacts.

2.1 Orthogonal projections (i)

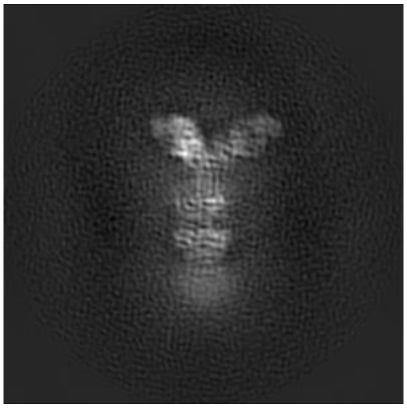

X

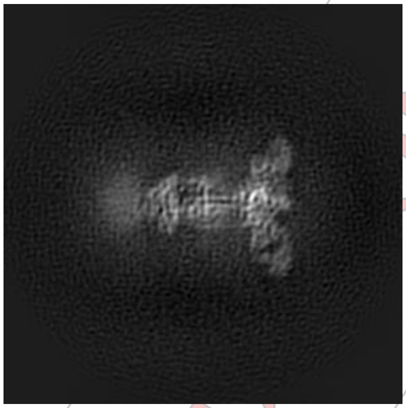

Y

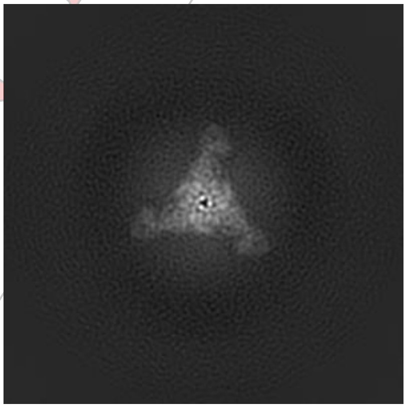

Z

The images above show the map projected in three orthogonal projections, in greyscale.

2.2 Central slices (i)

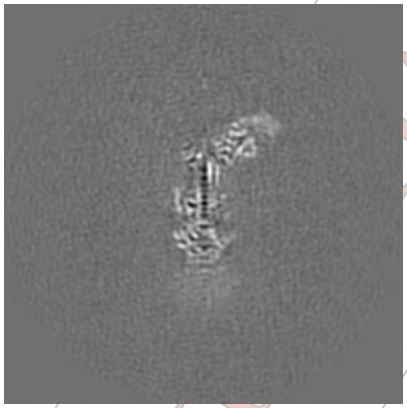

X

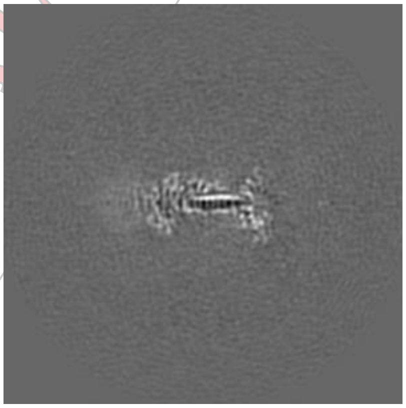

Y

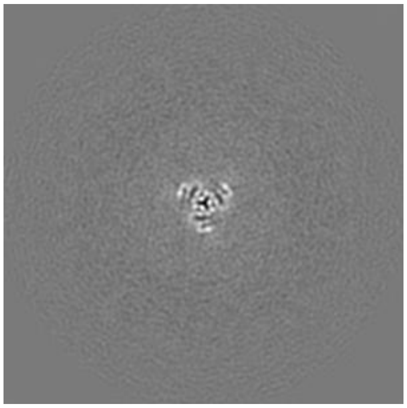

Z

The images above show central slices of the map in three orthogonal directions, in greyscale.

CONFIDENTIAL VALIDATION REPORT

2.3 Largest variance slices ⓘ

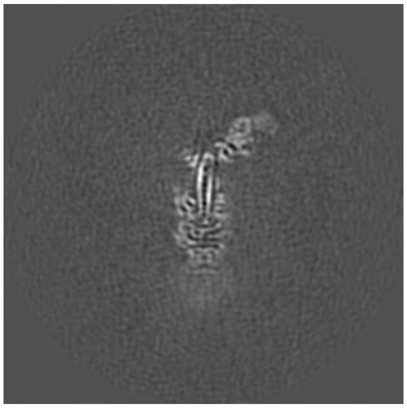
X Index: 98

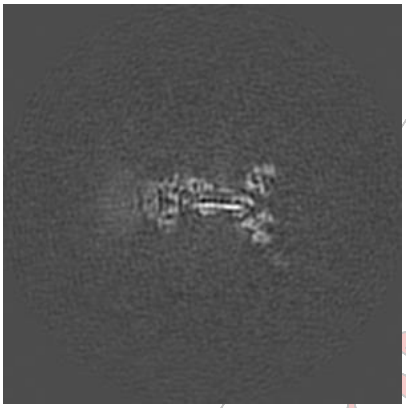
Y Index: 98

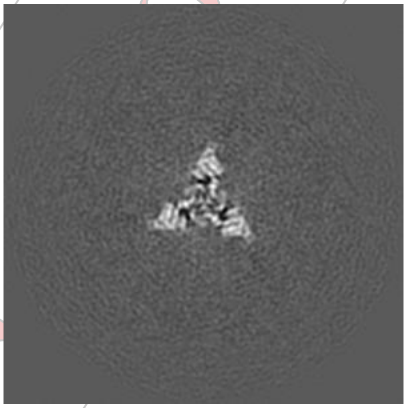
Z Index: 125

The images above show the highest variance slices of the map in three orthogonal directions, in greyscale.

2.4 Orthogonal surface views ⓘ

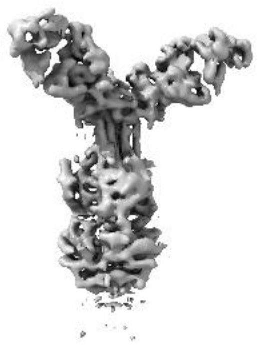
X

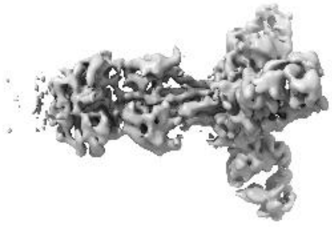
Y

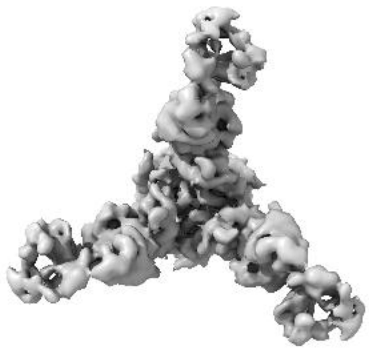
Z

The images above show the 3D surface view of the map at the recommended contour level 2.16. This in conjunction with the slice images can indicate whether an appropriate contour level has been selected.

2.5 Mask visualisation ⓘ

This section was not generated. No masks were provided.

CONFIDENTIAL - VALIDATION REPORT

3 Map analysis (i)

This section contains the results of statistical analysis of the map.

3.1 Map-value distribution (i)

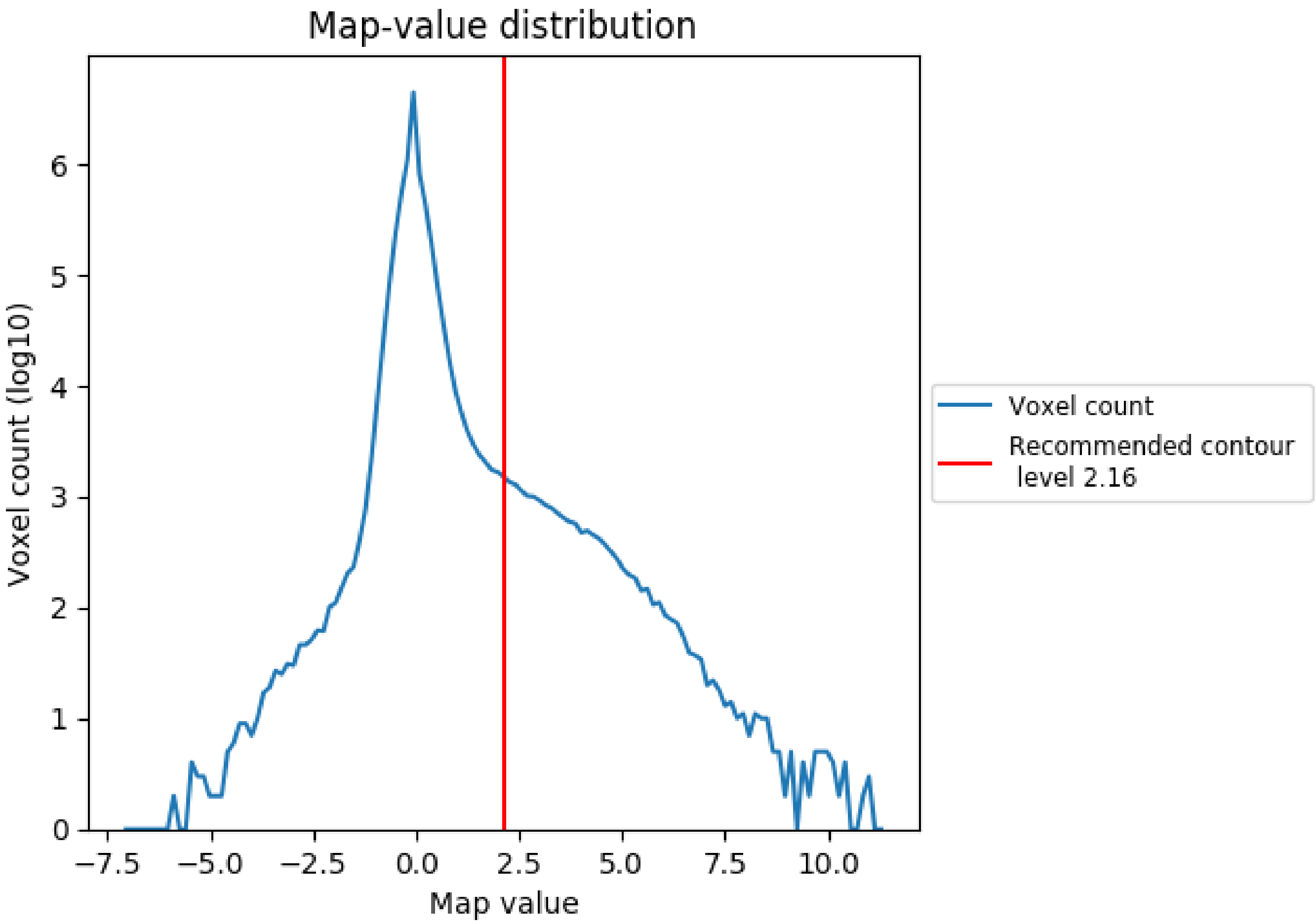

The map-value distribution is plotted in 128 intervals along the x-axis. The y-axis is logarithmic. A spike in this graph at zero usually indicates that the volume has been masked.

CONFIDENTIAL REPORT

3.2 Volume estimate (i)

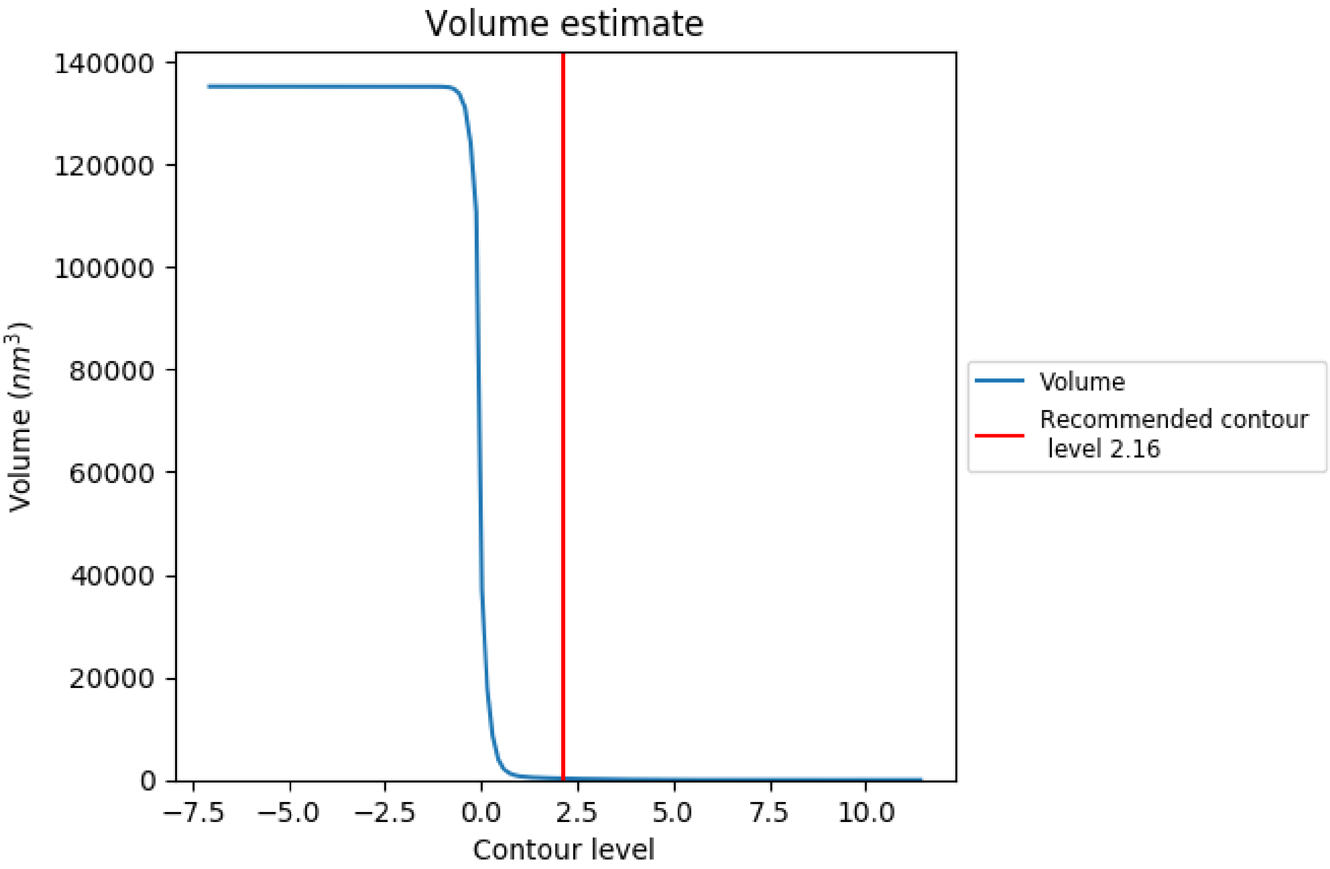

The volume at the recommended contour level is 277 nm^3; this corresponds to an approximate mass of 250 kDa.

The volume estimate graph shows how the enclosed volume varies with the contour level. The recommended contour level is shown as a vertical line and the intersection between the line and the curve gives the volume of the enclosed surface at the given level.

CONFIDENTIAL REPORT

3.3 Rotationally averaged power spectrum (i)

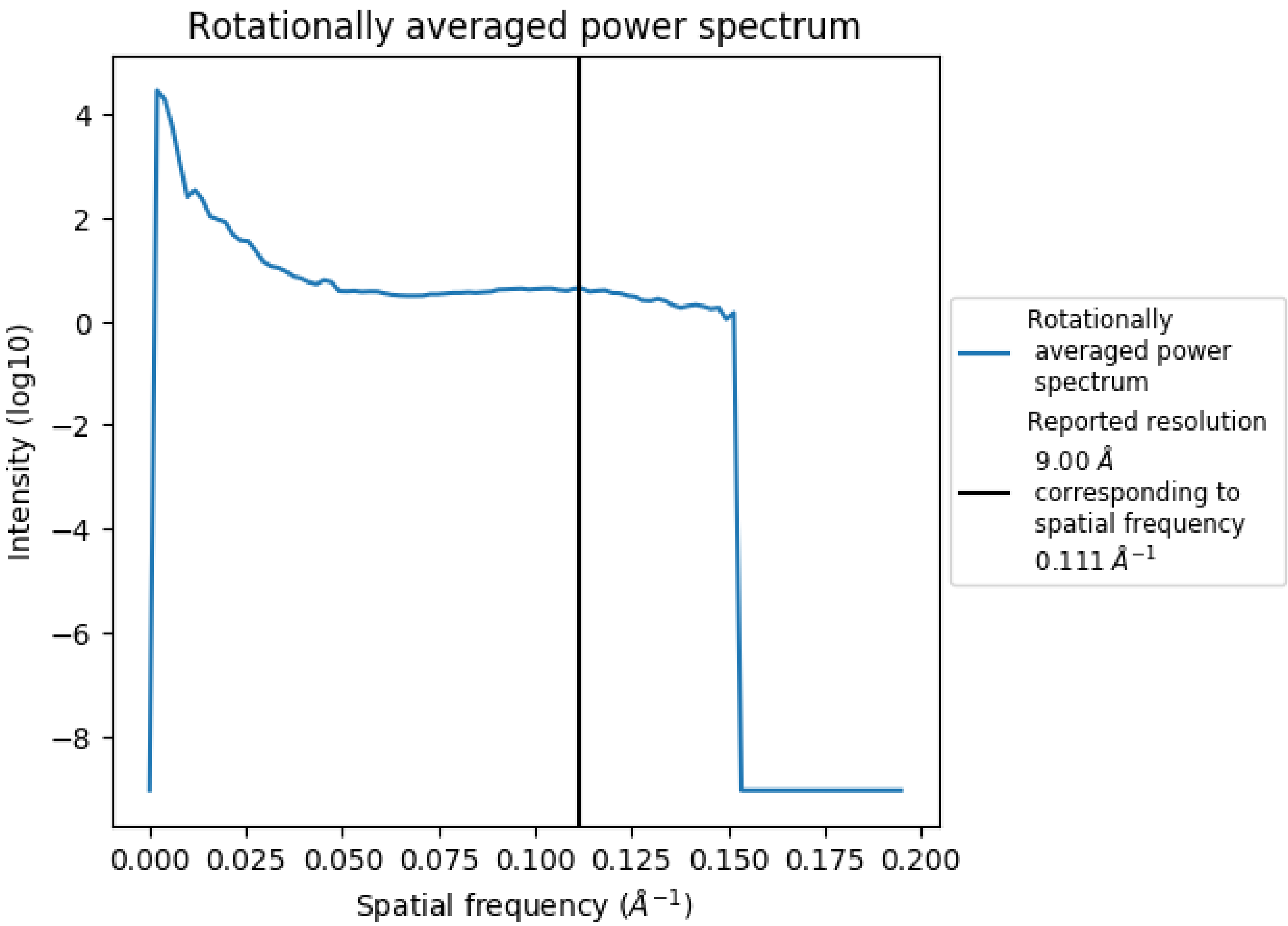

CONFIDENTIAL REPORT

4 Fourier-Shell correlation (i)

This section was not generated. No FSC curve or half maps provided.

CONFIDENTIAL VALIDATION REPORT
