# Supplementary material for: The N-terminus of varicella-zoster virus glycoprotein B has a functional role in fusion
Source: PLoS Pathog. 2021 Jan 7;17(1):e1008961. doi: 10.1371/journal.ppat.1008961 (PMC7817050; doi:10.1371/journal.ppat.1008961)
Supplement: S3 Validation Report — (PDF) [file ppat.1008961.s021.pdf]

Preliminary Full wwPDB EM Map/Model Validation Report (i)

Sep 8, 2020 – 11:02 AM EDT

Deposition ID : D_1000251554

This is a Preliminary Full wwPDB EM Map/Model Validation Report.

This report is produced by the wwPDB Deposition System during initial deposition but before annotation of the structure.

We welcome your comments at *validation@mail.wwpdb.org*
A user guide is available at
https://www.wwpdb.org/validation/2017/EMValidationReportHelp
with specific help available everywhere you see the (i) symbol.

PRELIMINARY VALIDATION REPORT

The following versions of software and data (see references (i)) were used in the production of this report:

EMDB validation analysis	:	0.0.0.dev33
Mogul	:	1.8.5 (274361), CSD as541be (2020)
MolProbity	:	4.02b-467
Percentile statistics	:	20191225.v01 (using entries in the PDB archive December 25th 2019)
Ideal geometry (proteins)	:	Engh & Huber (2001)
Ideal geometry (DNA, RNA)	:	Parkinson et al. (1996)
Validation Pipeline (wwPDB-VP)	:	2.14.2

1 Overall quality at a glance (i)

The following experimental techniques were used to determine the structure:
ELECTRON MICROSCOPY

The reported resolution of this entry is 3.90 Å.

Percentile scores (ranging between 0-100) for global validation metrics of the entry are shown in the following graphic. The table shows the number of entries on which the scores are based.

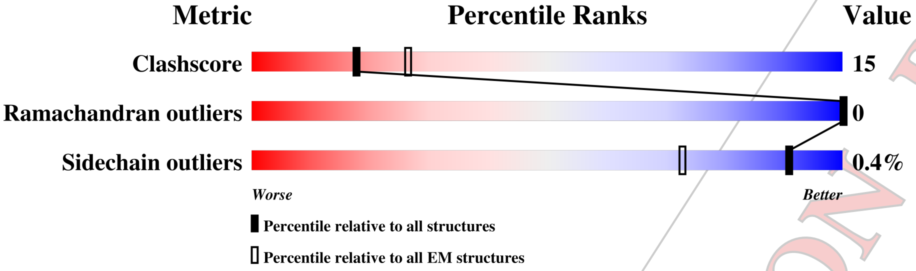

Metric	Whole archive (#Entries)	EM structures (#Entries)
Clashscore	158937	4297
Ramachandran outliers	154571	4023
Sidechain outliers	154315	3826

The table below summarises the geometric issues observed across the polymeric chains and their fit to the map. The red, orange, yellow and green segments on the bar indicate the fraction of residues that contain outliers for >=3, 2, 1 and 0 types of geometric quality criteria respectively. A grey segment represents the fraction of residues that are not modelled. The numeric value for each fraction is indicated below the corresponding segment, with a dot representing fractions <=5% The upper red bar (where present) indicates the fraction of residues that have poor fit to the EM map (all atom inclusion < 40%). The numeric value is given above the bar.

Mol	Chain	Length	Quality of chain
1	A	931	• / 45% / 17% / 37%
1	B	931	• / 45% / 17% / 37%
1	C	931	• / 45% / 17% / 37%
2	D	6	17% / 33% / 50% / 17%
2	E	6	17% / 33% / 33% / 33%
2	F	6	17% / 33% / 50% / 17%

PRELIMINARY VALIDATION REPORT

2 Entry composition (i)

There are 3 unique types of molecules in this entry. The entry contains 14409 atoms, of which 0 are hydrogens and 0 are deuteriums.

In the tables below, the AltConf column contains the number of residues with at least one atom in alternate conformation and the Trace column contains the number of residues modelled with at most 2 atoms.

- Molecule 1 is a protein called Varicella-zoster virus glycoprotein B.

Mol	Chain	Residues	Atoms					AltConf	Trace
			Total	C	N	O	S		
1	A	584	4717	2981	823	891	22	0	0
1	B	584	4717	2981	823	891	22	0	0
1	C	584	4717	2981	823	891	22	0	0

- Molecule 2 is an oligosaccharide called alpha-D-mannopyranose-(1-3)-alpha-D-mannopyranose-(1-6)-[alpha-D-mannopyranose-(1-3)]alpha-D-mannopyranose-(1-4)-2-acetamido-2-deoxy-beta-D-glucopyranose-(1-4)-2-acetamido-2-deoxy-beta-D-glucopyranose.

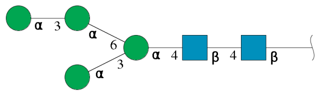

Mol	Chain	Residues	Atoms				AltConf	Trace
			Total	C	N	O		
2	D	6	72	40	2	30	0	0
2	E	6	72	40	2	30	0	0
2	F	6	72	40	2	30	0	0

- Molecule 3 is 2-acetamido-2-deoxy-beta-D-glucopyranose (three-letter code: NAG) (formula: $C_8H_{15}NO_6$).

PRELIMINARY VALIDATION REPORT

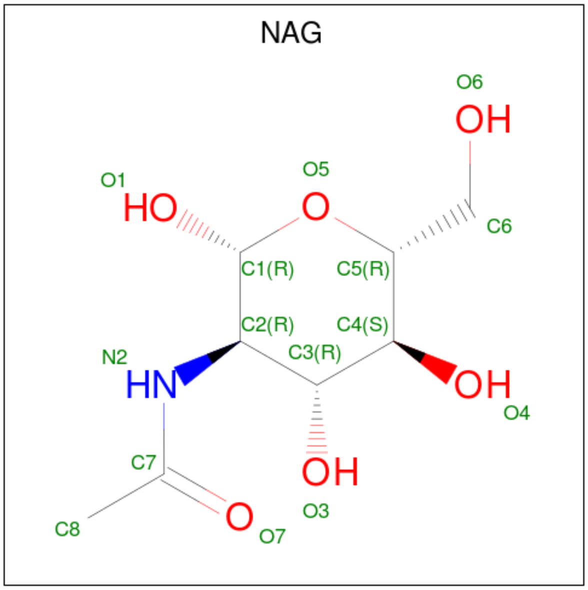

Mol	Chain	Residues	Atoms	AltConf
3	A	1	Total C N O<br>14 8 1 5	0
3	B	1	Total C N O<br>14 8 1 5	0
3	C	1	Total C N O<br>14 8 1 5	0

PRELIMINARY VALIDATION REPORT

3 Residue-property plots ⓘ

These plots are drawn for all protein, RNA, DNA and oligosaccharide chains in the entry. The first graphic for a chain summarises the proportions of the various outlier classes displayed in the second graphic. The second graphic shows the sequence view annotated by issues in geometry and atom inclusion in map density. Residues are color-coded according to the number of geometric quality criteria for which they contain at least one outlier: green = 0, yellow = 1, orange = 2 and red = 3 or more. A red diamond above a residue indicates a poor fit to the EM map for this residue (all atom inclusion < 40%). Stretches of 2 or more consecutive residues without any outlier are shown as a green connector. Residues present in the sample, but not in the model, are shown in grey.

- Molecule 1: Varicella-zoster virus glycoprotein B

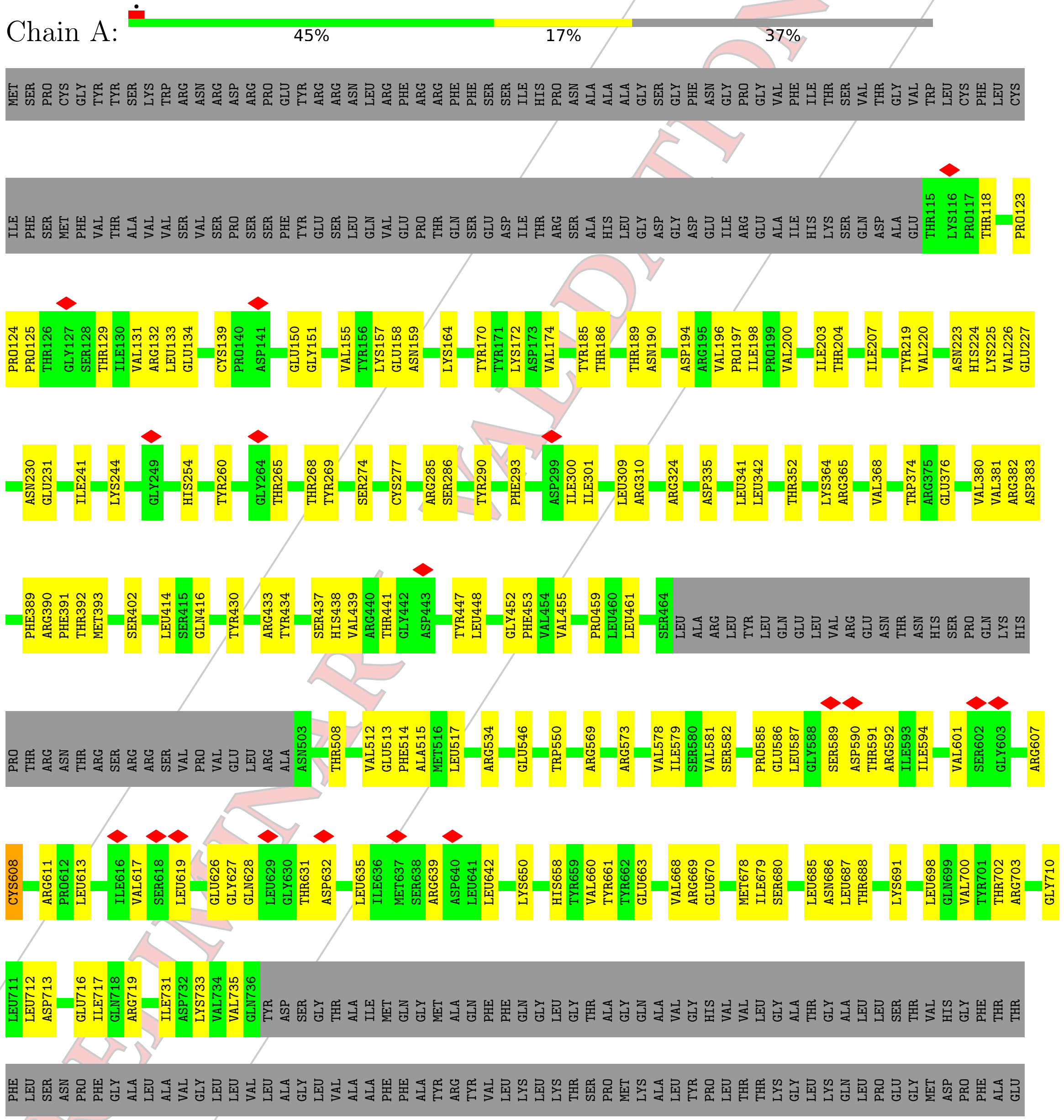

PRELIMINARY VALIDATION REPORT

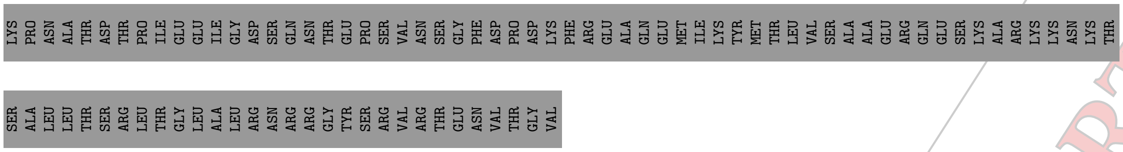

• Molecule 1: Varicella-zoster virus glycoprotein B

Chain B:

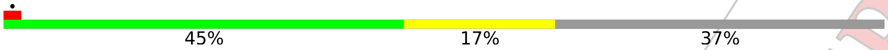

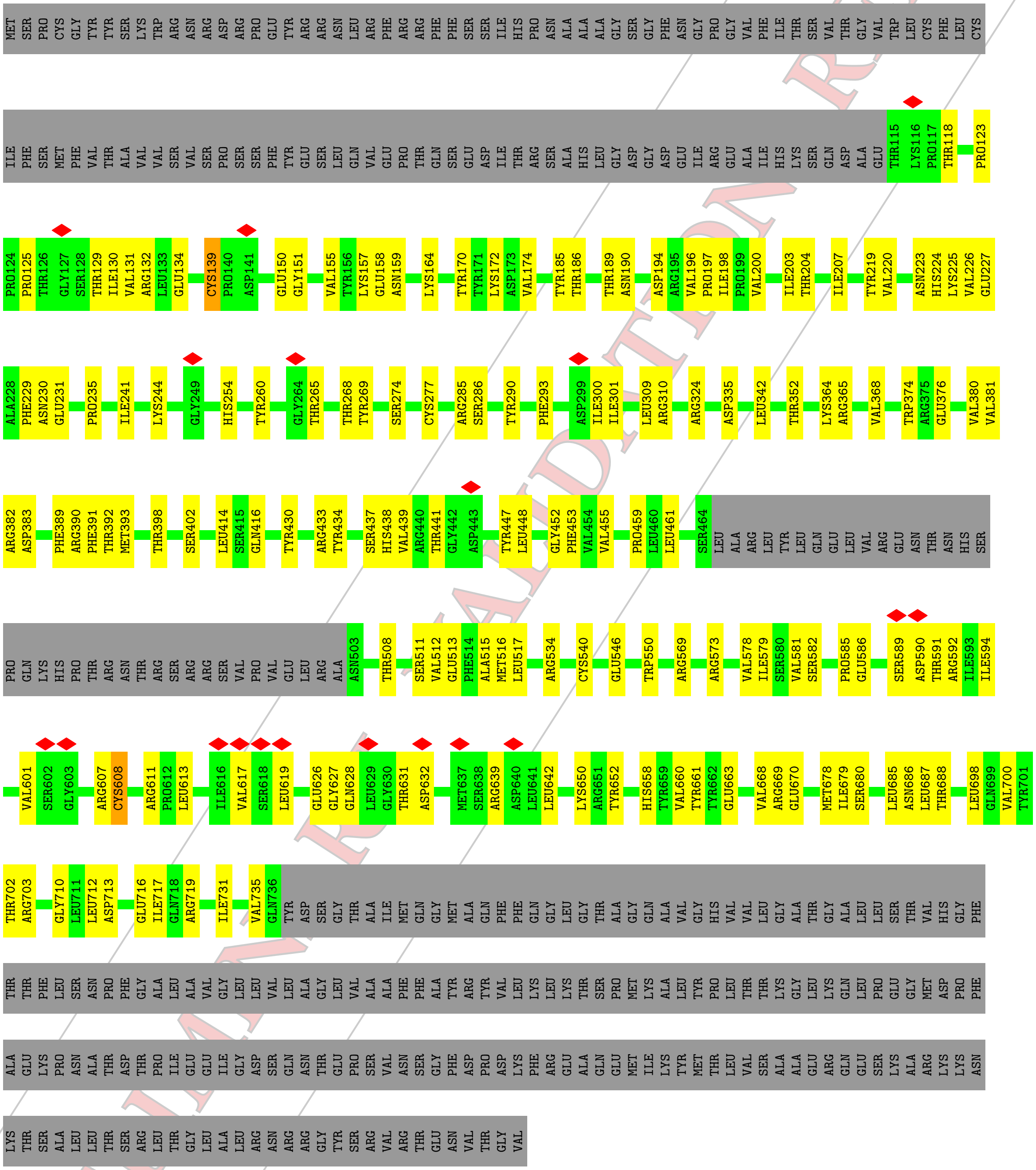

• Molecule 1: Varicella-zoster virus glycoprotein B

Chain C:

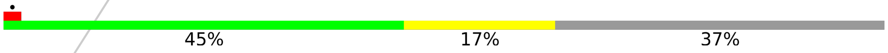

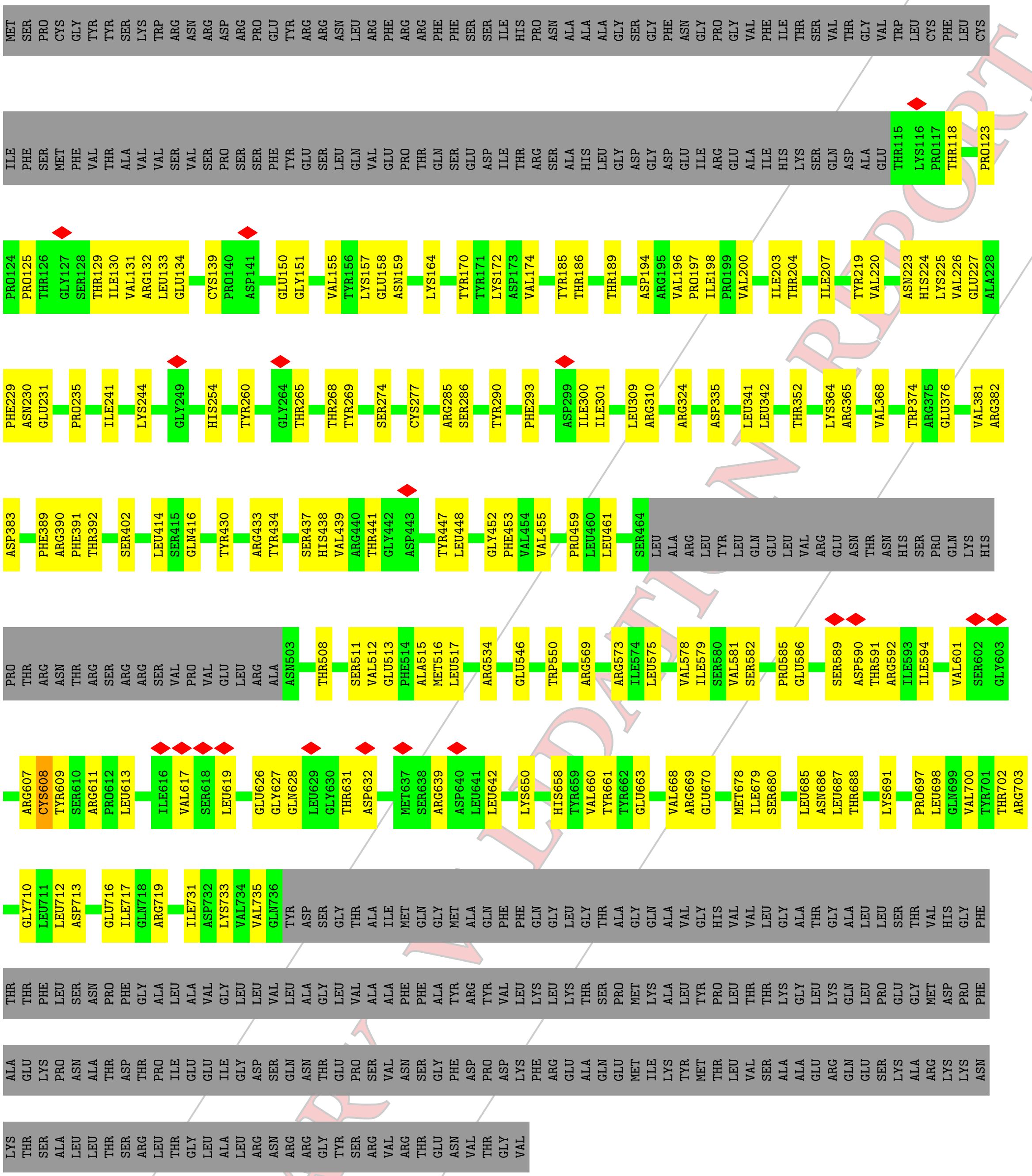

- Molecule 2: alpha-D-mannopyranose-(1-3)-alpha-D-mannopyranose-(1-6)-[alpha-D-mannopyranose-(1-3)]alpha-D-mannopyranose-(1-4)-2-acetamido-2-deoxy-beta-D-glucopyranose-(1-4)-2-acetamido-2-deoxy-beta-D-glucopyranose

Chain D:

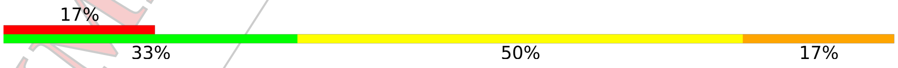

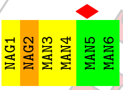

- Molecule 2: alpha-D-mannopyranose-(1-3)-alpha-D-mannopyranose-(1-6)-[alpha-D-mannopyranose-(1-3)]alpha-D-mannopyranose-(1-4)-2-acetamido-2-deoxy-beta-D-glucopyranose-(1-4)-2-aceta

PRELIMINARY VALIDATION REPORT

mido-2-deoxy-beta-D-glucopyranose

Chain E: 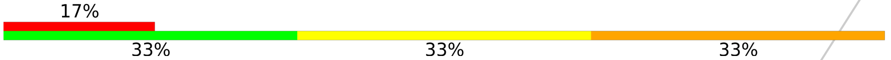

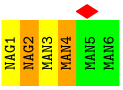

• Molecule 2: alpha-D-mannopyranose-(1-3)-alpha-D-mannopyranose-(1-6)-[alpha-D-mannopyranose-(1-3)]alpha-D-mannopyranose-(1-4)-2-acetamido-2-deoxy-beta-D-glucopyranose-(1-4)-2-acetamido-2-deoxy-beta-D-glucopyranose

Chain F: 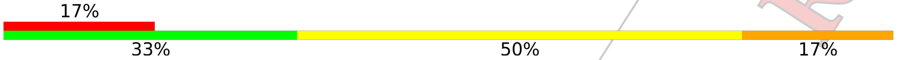

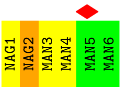

PRELIMINARY VALIDATION REPORT

4 Experimental information (i)

Property	Value	Source
EM reconstruction method	Not provided	Depositor
Imposed symmetry	POINT, Not provided	Depositor
Number of images used	Not provided	Depositor
Resolution determination method	Not provided	Depositor
CTF correction method	Not provided	Depositor
Microscope	Not provided	Depositor
Voltage (kV)	Not provided	Depositor
Electron dose ($e^-/\text{Å}^2$)	Not provided	Depositor
Minimum defocus (nm)	Not provided	Depositor
Maximum defocus (nm)	Not provided	Depositor
Magnification	Not provided	Depositor
Image detector	Not provided	Depositor
Maximum map value	0.148	Depositor
Minimum map value	-0.083	Depositor
Average map value	0.000	Depositor
Map value standard deviation	0.002	Depositor
Recommended contour level	0.02	Depositor
Map size (Å)	423.99997, 423.99997, 423.99997	Depositor
Map dimensions	400, 400, 400	Depositor
Map angles (°)	90.0, 90.0, 90.0	Depositor
Pixel spacing (Å)	1.06, 1.06, 1.06	Depositor

PRELIMINARY VALIDATION REPORT

5 Model quality (i)

5.1 Standard geometry (i)

Bond lengths and bond angles in the following residue types are not validated in this section: NAG, MAN

The Z score for a bond length (or angle) is the number of standard deviations the observed value is removed from the expected value. A bond length (or angle) with $|Z| > 5$ is considered an outlier worth inspection. RMSZ is the root-mean-square of all Z scores of the bond lengths (or angles).

Mol	Chain	Bond lengths		Bond angles	
		RMSZ	#$\|Z\|$ >5	RMSZ	#$\|Z\|$ >5
1	A	0.36	0/4827	0.54	0/6555
1	B	0.36	0/4827	0.54	0/6555
1	C	0.36	0/4827	0.54	0/6555
All	All	0.36	0/14481	0.54	0/19665

There are no bond length outliers.

There are no bond angle outliers.

There are no chirality outliers.

There are no planarity outliers.

5.2 Too-close contacts (i)

In the following table, the Non-H and H(model) columns list the number of non-hydrogen atoms and hydrogen atoms in the chain respectively. The H(added) column lists the number of hydrogen atoms added and optimized by MolProbity. The Clashes column lists the number of clashes within the asymmetric unit, whereas Symm-Clashes lists symmetry related clashes.

Mol	Chain	Non-H	H(model)	H(added)	Clashes	Symm-Clashes
1	A	4717	0	4597	177	0
1	B	4717	0	4597	188	0
1	C	4717	0	4597	177	0
2	D	72	0	61	8	0
2	E	72	0	61	7	0
2	F	72	0	61	8	0
3	A	14	0	13	0	0
3	B	14	0	13	0	0
3	C	14	0	13	0	0
All	All	14409	0	14013	425	0

PRELIMINARY VALIDATION REPORT

The all-atom clashscore is defined as the number of clashes found per 1000 atoms (including hydrogen atoms). The all-atom clashscore for this structure is 15.

All (425) close contacts within the same asymmetric unit are listed below, sorted by their clash magnitude.

Atom-1	Atom-2	Interatomic distance (Å)	Clash overlap (Å)
1:B:512:VAL:CG1	1:C:698:LEU:HD22	1.51	1.40
1:B:512:VAL:HG11	1:C:698:LEU:CD2	1.57	1.33
1:B:439:VAL:HG23	1:B:461:LEU:HD11	1.27	1.16
1:B:512:VAL:HG21	1:C:698:LEU:CD2	1.76	1.14
1:A:439:VAL:HG23	1:A:461:LEU:HD11	1.29	1.14
1:A:513:GLU:OE2	1:B:513:GLU:OE2	1.65	1.13
1:C:439:VAL:HG23	1:C:461:LEU:HD11	1.29	1.12
1:C:241:ILE:HB	2:F:1:NAG:H83	1.32	1.11
1:A:441:THR:HB	1:A:461:LEU:HD23	1.28	1.10
1:A:241:ILE:HB	2:D:1:NAG:H83	1.33	1.09
1:B:441:THR:HB	1:B:461:LEU:HD23	1.26	1.08
1:A:513:GLU:OE2	1:B:513:GLU:CD	1.90	1.08
1:B:241:ILE:HB	2:E:1:NAG:H83	1.31	1.07
1:C:441:THR:HB	1:C:461:LEU:HD23	1.28	1.05
1:A:513:GLU:OE1	1:B:513:GLU:OE1	1.77	1.01
1:A:700:VAL:CG2	1:C:512:VAL:HG23	1.90	1.00
1:C:512:VAL:O	1:C:516:MET:HG3	1.61	1.00
1:A:513:GLU:CD	1:B:513:GLU:CD	2.20	0.99
1:A:700:VAL:HG22	1:C:512:VAL:CG2	1.95	0.97
1:A:517:LEU:CD1	1:B:513:GLU:OE2	2.12	0.97
1:B:512:VAL:HG21	1:C:698:LEU:HD23	1.44	0.97
1:B:678:MET:HE1	1:C:134:GLU:HA	1.48	0.95
1:A:134:GLU:HA	1:C:678:MET:HE1	1.46	0.94
1:B:439:VAL:HG23	1:B:461:LEU:CD1	1.99	0.91
1:A:439:VAL:HG23	1:A:461:LEU:CD1	2.02	0.90
1:C:439:VAL:HG23	1:C:461:LEU:CD1	2.02	0.88
1:A:700:VAL:HG23	1:C:512:VAL:HG23	1.55	0.86
1:B:512:VAL:CG2	1:C:698:LEU:CD2	2.52	0.86
1:A:517:LEU:HD11	1:B:513:GLU:OE2	1.77	0.84
1:B:512:VAL:HG21	1:C:698:LEU:HD21	1.59	0.82
1:A:626:GLU:OE1	1:A:639:ARG:NH2	2.13	0.82
1:C:626:GLU:OE1	1:C:639:ARG:NH2	2.13	0.82
1:A:513:GLU:OE1	1:B:513:GLU:CD	2.18	0.82
1:B:512:VAL:HG23	1:C:700:VAL:CG2	2.09	0.81
1:B:626:GLU:OE1	1:B:639:ARG:NH2	2.13	0.80
1:C:241:ILE:HB	2:F:1:NAG:C8	2.10	0.80
1:B:607:ARG:HD2	1:B:642:LEU:HB3	1.65	0.79

Continued on next page...

PRELIMINARY VALIDATION REPORT

Continued from previous page...

Atom-1	Atom-2	Interatomic distance (Å)	Clash overlap (Å)
1:C:607:ARG:HD2	1:C:642:LEU:HB3	1.65	0.79
1:A:285:ARG:NH2	1:B:710:GLY:O	2.16	0.79
1:A:241:ILE:HB	2:D:1:NAG:C8	2.11	0.78
1:A:710:GLY:O	1:C:285:ARG:NH2	2.17	0.78
1:B:285:ARG:NH2	1:C:710:GLY:O	2.17	0.78
1:A:374:TRP:CH2	1:B:698:LEU:HD21	2.18	0.78
1:A:607:ARG:HD2	1:A:642:LEU:HB3	1.65	0.78
1:B:512:VAL:HG23	1:C:700:VAL:HG22	1.66	0.78
1:A:678:MET:HE1	1:B:134:GLU:HA	1.64	0.77
1:B:241:ILE:HB	2:E:1:NAG:C8	2.11	0.77
1:B:374:TRP:CH2	1:C:698:LEU:HD21	2.19	0.77
1:B:374:TRP:CZ2	1:C:698:LEU:HD21	2.20	0.77
1:A:374:TRP:CZ2	1:B:698:LEU:HD21	2.21	0.76
1:B:678:MET:HE3	1:C:132:ARG:HG3	1.69	0.74
1:A:132:ARG:HG3	1:C:678:MET:HE3	1.70	0.73
1:C:365:ARG:O	1:C:416:GLN:NE2	2.21	0.73
1:A:512:VAL:HG23	1:B:700:VAL:HG22	1.69	0.73
1:A:365:ARG:O	1:A:416:GLN:NE2	2.21	0.73
1:A:513:GLU:HG3	1:C:513:GLU:OE1	1.88	0.72
1:A:439:VAL:CG2	1:A:461:LEU:HD11	2.14	0.72
1:A:534:ARG:NH1	1:B:686:ASN:O	2.21	0.72
1:A:686:ASN:O	1:C:534:ARG:NH1	2.22	0.72
1:B:439:VAL:CG2	1:B:461:LEU:HD11	2.14	0.72
1:B:365:ARG:O	1:B:416:GLN:NE2	2.21	0.71
1:A:203:ILE:HD13	1:A:207:ILE:HD12	1.72	0.71
1:C:439:VAL:CG2	1:C:461:LEU:HD11	2.14	0.71
1:B:534:ARG:NH1	1:C:686:ASN:O	2.23	0.70
1:A:700:VAL:HG22	1:C:512:VAL:HG22	1.73	0.70
1:A:592:ARG:HE	1:A:594:ILE:HD11	1.57	0.69
1:B:592:ARG:HE	1:B:594:ILE:HD11	1.57	0.69
1:B:203:ILE:HD13	1:B:207:ILE:HD12	1.72	0.69
1:A:134:GLU:HA	1:C:678:MET:CE	2.21	0.69
1:C:203:ILE:HD13	1:C:207:ILE:HD12	1.72	0.69
1:A:678:MET:CE	1:B:134:GLU:HA	2.23	0.69
1:A:698:LEU:HD21	1:C:374:TRP:CH2	2.28	0.69
1:A:700:VAL:CG2	1:C:512:VAL:CG2	2.57	0.69
1:C:592:ARG:HE	1:C:594:ILE:HD11	1.57	0.68
1:B:441:THR:HB	1:B:461:LEU:CD2	2.15	0.67
1:A:441:THR:HB	1:A:461:LEU:CD2	2.18	0.67
1:A:679:ILE:HG21	1:B:131:VAL:HG21	1.76	0.66
1:B:679:ILE:HG21	1:C:131:VAL:HG21	1.77	0.66

Continued on next page...

PRELIMINARY VALIDATION REPORT

Continued from previous page...

Atom-1	Atom-2	Interatomic distance (Å)	Clash overlap (Å)
1:B:678:MET:CE	1:C:134:GLU:HA	2.25	0.66
1:B:439:VAL:O	1:B:461:LEU:HG	1.96	0.65
1:A:131:VAL:HG21	1:C:679:ILE:HG21	1.77	0.65
1:A:439:VAL:O	1:A:461:LEU:HG	1.97	0.65
1:C:439:VAL:O	1:C:461:LEU:HG	1.96	0.65
1:B:376:GLU:N	1:B:376:GLU:OE1	2.29	0.64
1:A:678:MET:HE3	1:B:132:ARG:HG3	1.79	0.64
1:A:376:GLU:N	1:A:376:GLU:OE1	2.29	0.64
1:A:698:LEU:HD21	1:C:374:TRP:CZ2	2.32	0.64
1:A:716:GLU:OE2	1:A:719:ARG:NH2	2.31	0.64
1:B:512:VAL:CG2	1:C:700:VAL:HG22	2.28	0.64
1:C:441:THR:HB	1:C:461:LEU:CD2	2.18	0.63
1:B:512:VAL:O	1:B:516:MET:HG3	1.99	0.63
2:E:2:NAG:H61	2:E:3:MAN:H2	1.79	0.63
2:F:2:NAG:H61	2:F:3:MAN:H2	1.80	0.63
1:C:660:VAL:HG12	1:C:670:GLU:HG3	1.81	0.63
1:A:517:LEU:HD12	1:B:513:GLU:OE2	1.96	0.63
1:C:376:GLU:N	1:C:376:GLU:OE1	2.29	0.63
1:B:716:GLU:OE2	1:B:719:ARG:NH2	2.31	0.63
1:B:660:VAL:HG12	1:B:670:GLU:HG3	1.81	0.62
1:C:716:GLU:OE2	1:C:719:ARG:NH2	2.31	0.62
1:A:680:SER:HA	1:B:134:GLU:OE1	1.99	0.62
1:B:512:VAL:CB	1:C:698:LEU:CD2	2.77	0.62
1:C:512:VAL:O	1:C:512:VAL:HG12	2.00	0.62
1:A:134:GLU:OE1	1:C:680:SER:HA	1.99	0.62
1:A:660:VAL:HG12	1:A:670:GLU:HG3	1.81	0.62
1:C:364:LYS:HE2	1:C:414:LEU:HD21	1.81	0.62
1:B:512:VAL:CG2	1:C:698:LEU:HD23	2.23	0.61
2:D:2:NAG:H61	2:D:3:MAN:H2	1.81	0.61
1:B:241:ILE:CB	2:E:1:NAG:H83	2.19	0.61
1:B:364:LYS:HE2	1:B:414:LEU:HD21	1.81	0.61
1:A:364:LYS:HE2	1:A:414:LEU:HD21	1.81	0.61
1:A:512:VAL:HG23	1:B:700:VAL:CG2	2.30	0.61
1:C:155:VAL:HG12	1:C:455:VAL:HG22	1.83	0.61
1:C:241:ILE:CB	2:F:1:NAG:H83	2.20	0.61
1:A:512:VAL:HG12	1:A:515:ALA:HB3	1.82	0.60
1:A:679:ILE:HG23	1:B:131:VAL:HB	1.85	0.59
1:B:512:VAL:CG1	1:C:698:LEU:CD2	2.39	0.59
1:A:155:VAL:HG12	1:A:455:VAL:HG22	1.83	0.59
1:A:534:ARG:HH12	1:B:688:THR:HG23	1.67	0.59
1:A:131:VAL:HB	1:C:679:ILE:HG23	1.85	0.59

Continued on next page...

PRELIMINARY VALIDATION REPORT

Continued from previous page...

Atom-1	Atom-2	Interatomic distance (Å)	Clash overlap (Å)
1:B:569:ARG:HH22	1:B:632:ASP:HB3	1.67	0.59
1:B:155:VAL:HG12	1:B:455:VAL:HG22	1.83	0.59
1:A:678:MET:HE2	1:B:134:GLU:N	2.18	0.58
1:A:513:GLU:CD	1:B:513:GLU:CG	2.72	0.58
1:A:569:ARG:HH22	1:A:632:ASP:HB3	1.67	0.58
1:C:569:ARG:HH22	1:C:632:ASP:HB3	1.67	0.58
1:C:430:TYR:OH	1:C:438:HIS:O	2.22	0.58
1:B:678:MET:HE2	1:C:134:GLU:N	2.19	0.57
1:A:241:ILE:CB	2:D:1:NAG:H83	2.21	0.57
1:A:189:THR:HG21	1:A:269:TYR:HE1	1.70	0.57
1:B:158:GLU:H	1:B:508:THR:HG21	1.70	0.57
1:A:224:HIS:CE1	1:C:735:VAL:HG11	2.39	0.57
1:A:430:TYR:OH	1:A:438:HIS:O	2.22	0.57
1:B:189:THR:HG21	1:B:269:TYR:HE1	1.70	0.57
1:A:134:GLU:N	1:C:678:MET:HE2	2.18	0.57
1:A:613:LEU:HD22	1:A:639:ARG:HD3	1.87	0.56
1:C:158:GLU:H	1:C:508:THR:HG21	1.70	0.56
1:B:511:SER:O	1:C:700:VAL:HG21	2.05	0.56
1:A:158:GLU:H	1:A:508:THR:HG21	1.70	0.56
1:B:680:SER:HA	1:C:134:GLU:OE1	2.04	0.56
1:C:189:THR:HG21	1:C:269:TYR:HE1	1.70	0.56
1:A:374:TRP:HH2	1:B:698:LEU:HD21	1.68	0.56
1:B:678:MET:CE	1:C:134:GLU:N	2.69	0.56
1:B:430:TYR:OH	1:B:438:HIS:O	2.22	0.56
1:C:613:LEU:HD22	1:C:639:ARG:HD3	1.87	0.56
1:B:613:LEU:HD22	1:B:639:ARG:HD3	1.87	0.56
1:A:134:GLU:CA	1:C:678:MET:CE	2.83	0.56
1:C:335:ASP:HB2	1:C:342:LEU:HD21	1.88	0.55
1:A:512:VAL:O	1:A:515:ALA:N	2.39	0.55
1:A:569:ARG:NH2	1:A:632:ASP:HB3	2.22	0.55
1:B:534:ARG:HH12	1:C:688:THR:HG23	1.71	0.55
1:B:374:TRP:HH2	1:C:698:LEU:HD21	1.70	0.55
1:C:569:ARG:NH2	1:C:632:ASP:HB3	2.22	0.55
1:A:679:ILE:CG1	1:B:132:ARG:O	2.55	0.55
1:B:679:ILE:HG23	1:C:131:VAL:HB	1.88	0.55
1:C:231:GLU:HG3	1:C:260:TYR:CG	2.42	0.55
1:A:134:GLU:N	1:C:678:MET:CE	2.69	0.55
1:A:231:GLU:HG3	1:A:260:TYR:CG	2.42	0.55
1:B:231:GLU:HG3	1:B:260:TYR:CG	2.42	0.55
1:C:589:SER:O	1:C:591:THR:HG23	2.07	0.55
2:D:2:NAG:H5	2:D:2:NAG:HN2	1.72	0.55

Continued on next page...

PRELIMINARY VALIDATION REPORT

Continued from previous page...

Atom-1	Atom-2	Interatomic distance (Å)	Clash overlap (Å)
1:A:118:THR:OG1	1:A:586:GLU:OE1	2.21	0.54
1:A:589:SER:O	1:A:591:THR:HG23	2.07	0.54
1:A:678:MET:CE	1:B:134:GLU:CA	2.85	0.54
1:B:452:GLY:O	1:B:508:THR:HG22	2.07	0.54
1:B:678:MET:CE	1:C:134:GLU:CA	2.85	0.54
1:A:335:ASP:HB2	1:A:342:LEU:HD21	1.88	0.54
1:A:517:LEU:HD12	1:B:513:GLU:OE1	2.07	0.54
1:B:569:ARG:NH2	1:B:632:ASP:HB3	2.22	0.54
1:A:452:GLY:O	1:A:508:THR:HG22	2.07	0.54
1:B:589:SER:O	1:B:591:THR:HG23	2.07	0.54
1:A:688:THR:HG23	1:C:534:ARG:HH12	1.72	0.54
1:A:132:ARG:O	1:C:679:ILE:CG1	2.55	0.54
1:C:512:VAL:O	1:C:516:MET:CG	2.46	0.54
1:A:150:GLU:HG3	1:A:151:GLY:N	2.23	0.54
1:B:735:VAL:HG11	1:C:224:HIS:CE1	2.43	0.54
1:B:335:ASP:HB2	1:B:342:LEU:HD21	1.88	0.54
1:A:517:LEU:HD12	1:B:513:GLU:CD	2.27	0.54
1:C:461:LEU:HD12	1:C:461:LEU:O	2.08	0.54
1:B:150:GLU:HG3	1:B:151:GLY:N	2.23	0.54
1:B:118:THR:OG1	1:B:586:GLU:OE1	2.21	0.54
1:C:150:GLU:HG3	1:C:151:GLY:N	2.23	0.54
1:C:452:GLY:O	1:C:508:THR:HG22	2.07	0.53
1:C:200:VAL:O	1:C:204:THR:HG22	2.09	0.53
1:B:381:VAL:HG23	1:B:392:THR:HB	1.91	0.53
1:A:381:VAL:HG23	1:A:392:THR:HB	1.91	0.53
1:B:461:LEU:HD12	1:B:461:LEU:O	2.08	0.53
1:A:512:VAL:O	1:A:515:ALA:HB3	2.08	0.53
1:B:220:VAL:HG22	1:B:225:LYS:HD2	1.90	0.53
1:A:678:MET:CE	1:B:134:GLU:N	2.72	0.53
1:C:170:TYR:HB3	1:C:198:ILE:HD12	1.91	0.53
1:C:286:SER:HB2	1:C:293:PHE:HB3	1.91	0.53
1:C:118:THR:OG1	1:C:586:GLU:OE1	2.21	0.53
1:A:461:LEU:HD12	1:A:461:LEU:O	2.08	0.53
1:A:220:VAL:HG22	1:A:225:LYS:HD2	1.91	0.52
1:A:679:ILE:HG12	1:B:132:ARG:O	2.09	0.52
1:B:286:SER:HB2	1:B:293:PHE:HB3	1.91	0.52
1:A:200:VAL:O	1:A:204:THR:HG22	2.09	0.52
1:B:170:TYR:HB3	1:B:198:ILE:HD12	1.91	0.52
1:A:286:SER:HB2	1:A:293:PHE:HB3	1.91	0.52
1:B:601:VAL:HG23	1:B:608:CYS:HA	1.92	0.52
1:A:700:VAL:HG21	1:C:511:SER:O	2.09	0.52

Continued on next page...

PRELIMINARY VALIDATION REPORT

Continued from previous page...

Atom-1	Atom-2	Interatomic distance (Å)	Clash overlap (Å)
1:A:735:VAL:HG11	1:B:224:HIS:CE1	2.45	0.52
1:C:220:VAL:HG22	1:C:225:LYS:HD2	1.91	0.52
1:A:132:ARG:O	1:C:679:ILE:HG12	2.09	0.52
1:A:170:TYR:HB3	1:A:198:ILE:HD12	1.91	0.52
1:B:590:ASP:OD2	1:B:619:LEU:HB2	2.10	0.52
1:C:381:VAL:HG23	1:C:392:THR:HB	1.91	0.52
1:C:590:ASP:OD2	1:C:619:LEU:HB2	2.10	0.52
1:B:300:ILE:HD11	1:C:712:LEU:HD11	1.92	0.51
1:B:200:VAL:O	1:B:204:THR:HG22	2.09	0.51
1:A:601:VAL:HG23	1:A:608:CYS:HA	1.91	0.51
1:A:590:ASP:OD2	1:A:619:LEU:HB2	2.10	0.51
1:C:601:VAL:HG23	1:C:608:CYS:HA	1.92	0.51
1:A:230:ASN:OD1	1:A:274:SER:HA	2.11	0.51
1:A:731:ILE:O	1:B:324:ARG:NH2	2.44	0.51
1:C:196:VAL:HG21	1:C:219:TYR:CD1	2.46	0.51
1:A:196:VAL:HG21	1:A:219:TYR:CD1	2.46	0.51
1:A:129:THR:OG1	1:A:581:VAL:O	2.29	0.50
1:A:658:HIS:HB3	1:A:670:GLU:HG2	1.93	0.50
1:A:300:ILE:HD11	1:B:712:LEU:HD11	1.93	0.50
1:B:398:THR:HG23	1:B:515:ALA:HB1	1.93	0.50
1:C:230:ASN:OD1	1:C:274:SER:HA	2.11	0.50
1:A:698:LEU:HD21	1:C:374:TRP:HH2	1.76	0.50
1:B:125:PRO:HD2	1:B:573:ARG:NH1	2.27	0.50
1:B:196:VAL:HG21	1:B:219:TYR:CD1	2.46	0.50
1:B:230:ASN:OD1	1:B:274:SER:HA	2.11	0.50
1:C:129:THR:OG1	1:C:581:VAL:O	2.29	0.50
1:B:157:LYS:HG2	1:B:374:TRP:HB2	1.93	0.50
1:B:702:THR:HG22	1:B:703:ARG:H	1.77	0.50
1:C:125:PRO:HD2	1:C:573:ARG:NH1	2.27	0.50
1:C:658:HIS:HB3	1:C:670:GLU:HG2	1.93	0.50
1:A:125:PRO:HD2	1:A:573:ARG:NH1	2.27	0.50
1:A:678:MET:HE2	1:B:134:GLU:CA	2.41	0.50
1:C:157:LYS:HG2	1:C:374:TRP:HB2	1.93	0.50
1:A:134:GLU:CA	1:C:678:MET:HE1	2.29	0.50
1:B:129:THR:OG1	1:B:581:VAL:O	2.29	0.50
1:C:383:ASP:OD1	1:C:390:ARG:HB3	2.12	0.50
1:A:383:ASP:OD1	1:A:390:ARG:HB3	2.12	0.50
1:A:702:THR:HG22	1:A:703:ARG:H	1.77	0.50
1:C:702:THR:HG22	1:C:703:ARG:H	1.76	0.50
1:B:650:LYS:HG2	1:B:663:GLU:OE2	2.12	0.50
1:B:658:HIS:HB3	1:B:670:GLU:HG2	1.93	0.50

Continued on next page...

PRELIMINARY VALIDATION REPORT

Continued from previous page...

Atom-1	Atom-2	Interatomic distance (Å)	Clash overlap (Å)
1:C:265:THR:O	1:C:268:THR:HG22	2.12	0.50
1:B:383:ASP:OD1	1:B:390:ARG:HB3	2.12	0.49
1:A:265:THR:O	1:A:268:THR:HG22	2.12	0.49
1:B:265:THR:O	1:B:268:THR:HG22	2.12	0.49
2:F:2:NAG:H5	2:F:2:NAG:HN2	1.76	0.49
1:A:157:LYS:HG2	1:A:374:TRP:HB2	1.93	0.49
1:A:650:LYS:HG2	1:A:663:GLU:OE2	2.12	0.49
1:A:712:LEU:HD11	1:C:300:ILE:HD11	1.93	0.49
1:A:244:LYS:HA	2:D:2:NAG:O3	2.13	0.49
1:C:650:LYS:HG2	1:C:663:GLU:OE2	2.12	0.49
1:B:125:PRO:HB3	1:B:582:SER:HB3	1.95	0.48
1:B:441:THR:CB	1:B:461:LEU:HD23	2.20	0.48
1:A:131:VAL:CB	1:C:679:ILE:HG23	2.44	0.48
1:B:679:ILE:CG1	1:C:132:ARG:O	2.61	0.48
1:C:125:PRO:HB3	1:C:582:SER:HB3	1.95	0.48
1:B:374:TRP:HZ2	1:C:698:LEU:HD21	1.76	0.48
1:A:391:PHE:HE1	1:A:402:SER:OG	1.97	0.48
1:C:733:LYS:HE3	1:C:733:LYS:HB2	1.66	0.48
1:A:324:ARG:NH2	1:C:731:ILE:O	2.47	0.47
1:B:685:LEU:HG	1:B:687:LEU:HG	1.96	0.47
1:C:391:PHE:HE1	1:C:402:SER:OG	1.97	0.47
1:C:578:VAL:HG12	1:C:579:ILE:N	2.29	0.47
1:C:513:GLU:C	1:C:515:ALA:N	2.67	0.47
1:A:164:LYS:NZ	1:A:285:ARG:HH11	2.12	0.47
1:A:125:PRO:HB3	1:A:582:SER:HB3	1.95	0.47
1:C:172:LYS:O	1:C:196:VAL:HG12	2.15	0.47
1:A:613:LEU:HD23	1:A:628:GLN:HB3	1.96	0.47
1:B:164:LYS:NZ	1:B:285:ARG:HH11	2.12	0.47
1:A:172:LYS:O	1:A:196:VAL:HG12	2.15	0.47
1:B:164:LYS:HG2	1:B:285:ARG:HG2	1.97	0.47
1:B:437:SER:OG	1:B:438:HIS:ND1	2.32	0.47
1:A:733:LYS:HE3	1:A:733:LYS:HB2	1.66	0.47
1:B:391:PHE:HE1	1:B:402:SER:OG	1.97	0.47
1:B:578:VAL:HG12	1:B:579:ILE:N	2.29	0.47
1:B:172:LYS:O	1:B:196:VAL:HG12	2.15	0.47
1:A:578:VAL:HG12	1:A:579:ILE:N	2.29	0.47
2:E:2:NAG:HN2	2:E:2:NAG:H5	1.79	0.47
1:A:290:TYR:CZ	1:A:368:VAL:HG21	2.51	0.46
1:B:512:VAL:HG23	1:C:700:VAL:HG23	1.94	0.46
1:A:685:LEU:HG	1:A:687:LEU:HG	1.96	0.46
1:C:613:LEU:HD23	1:C:628:GLN:HB3	1.96	0.46

Continued on next page...

PRELIMINARY VALIDATION REPORT

Continued from previous page...

Atom-1	Atom-2	Interatomic distance (Å)	Clash overlap (Å)
1:B:731:ILE:O	1:C:324:ARG:NH2	2.49	0.46
1:C:164:LYS:HG2	1:C:285:ARG:HG2	1.97	0.46
1:C:164:LYS:NZ	1:C:285:ARG:HH11	2.12	0.46
1:A:186:THR:O	1:C:226:VAL:HG13	2.15	0.46
1:A:679:ILE:HG23	1:B:131:VAL:CB	2.44	0.46
1:B:613:LEU:HD23	1:B:628:GLN:HB3	1.96	0.46
1:A:164:LYS:HG2	1:A:285:ARG:HG2	1.97	0.46
1:A:627:GLY:O	1:A:639:ARG:NE	2.48	0.46
1:A:517:LEU:CG	1:B:513:GLU:OE2	2.61	0.46
1:A:517:LEU:CD1	1:B:513:GLU:CD	2.79	0.46
1:B:290:TYR:CZ	1:B:368:VAL:HG21	2.51	0.46
1:B:679:ILE:HG12	1:C:132:ARG:O	2.15	0.46
1:B:512:VAL:HG11	1:C:698:LEU:HD22	0.62	0.46
1:A:226:VAL:HG13	1:B:186:THR:O	2.16	0.46
1:A:679:ILE:HG13	1:B:132:ARG:O	2.15	0.46
1:A:735:VAL:HG12	1:B:223:ASN:O	2.16	0.46
1:C:290:TYR:CZ	1:C:368:VAL:HG21	2.51	0.46
1:B:158:GLU:HG2	1:B:159:ASN:N	2.31	0.46
1:C:158:GLU:HG2	1:C:159:ASN:N	2.31	0.46
1:A:513:GLU:CD	1:B:513:GLU:HG2	2.36	0.45
1:B:679:ILE:HG23	1:C:131:VAL:CB	2.45	0.45
1:C:685:LEU:HG	1:C:687:LEU:HG	1.96	0.45
1:B:448:LEU:HD12	1:B:453:PHE:O	2.16	0.45
1:C:448:LEU:HD12	1:C:453:PHE:O	2.16	0.45
1:A:132:ARG:O	1:C:679:ILE:HG13	2.17	0.45
1:B:301:ILE:HD12	1:B:352:THR:HG21	1.98	0.45
1:B:627:GLY:O	1:B:639:ARG:NE	2.48	0.45
1:C:592:ARG:O	1:C:617:VAL:HG22	2.17	0.45
1:B:439:VAL:CG2	1:B:461:LEU:CD1	2.84	0.45
1:A:448:LEU:HD12	1:A:453:PHE:O	2.16	0.45
1:B:441:THR:HG22	1:B:459:PRO:O	2.17	0.45
1:A:374:TRP:HZ2	1:B:698:LEU:HD21	1.78	0.45
1:B:652:TYR:N	1:C:575:LEU:O	2.37	0.45
1:A:592:ARG:O	1:A:617:VAL:HG22	2.17	0.45
1:B:226:VAL:HG13	1:C:186:THR:O	2.17	0.45
1:B:678:MET:CE	1:C:133:LEU:C	2.85	0.45
1:C:513:GLU:C	1:C:515:ALA:H	2.21	0.45
1:A:341:LEU:HA	1:A:341:LEU:HD23	1.80	0.45
1:A:301:ILE:HD12	1:A:352:THR:HG21	1.98	0.45
1:A:513:GLU:OE2	1:B:513:GLU:CG	2.64	0.45
1:A:441:THR:HG22	1:A:459:PRO:O	2.17	0.44

Continued on next page...

PRELIMINARY VALIDATION REPORT

Continued from previous page...

Atom-1	Atom-2	Interatomic distance (Å)	Clash overlap (Å)
1:A:691:LYS:HA	1:A:691:LYS:HD3	1.75	0.44
1:A:174:VAL:HG13	1:A:194:ASP:HB3	1.99	0.44
1:B:592:ARG:O	1:B:617:VAL:HG22	2.17	0.44
1:B:382:ARG:HD2	1:B:389:PHE:CD1	2.53	0.44
1:C:301:ILE:HD12	1:C:352:THR:HG21	1.98	0.44
1:C:244:LYS:HA	2:F:2:NAG:O3	2.16	0.44
1:C:441:THR:HG22	1:C:459:PRO:O	2.17	0.44
1:A:382:ARG:HD2	1:A:389:PHE:CD1	2.53	0.44
1:C:174:VAL:HG13	1:C:194:ASP:HB3	1.99	0.44
1:C:691:LYS:HD3	1:C:691:LYS:HA	1.75	0.44
1:A:133:LEU:C	1:C:678:MET:CE	2.86	0.44
1:A:158:GLU:HG2	1:A:159:ASN:N	2.31	0.43
1:A:661:TYR:HB3	1:A:669:ARG:HG2	2.00	0.43
1:A:517:LEU:HG	1:B:513:GLU:OE2	2.18	0.43
1:A:227:GLU:HG2	1:B:185:TYR:HB2	2.00	0.43
1:B:512:VAL:HG12	1:B:512:VAL:O	2.18	0.43
1:C:383:ASP:N	1:C:383:ASP:OD1	2.44	0.43
1:C:627:GLY:O	1:C:639:ARG:NE	2.48	0.43
1:C:661:TYR:HB3	1:C:669:ARG:HG2	2.00	0.43
1:C:341:LEU:HD23	1:C:341:LEU:HA	1.80	0.43
2:D:2:NAG:H5	2:D:2:NAG:N2	2.34	0.43
1:A:124:PRO:HA	1:A:125:PRO:HD3	1.84	0.43
1:B:735:VAL:HG12	1:C:223:ASN:O	2.19	0.43
1:C:382:ARG:HD2	1:C:389:PHE:CD1	2.53	0.43
1:B:174:VAL:HG13	1:B:194:ASP:HB3	1.99	0.43
1:B:227:GLU:HG2	1:C:185:TYR:HB2	2.01	0.43
1:C:437:SER:HG	1:C:438:HIS:CE1	2.29	0.43
2:E:3:MAN:H62	2:E:4:MAN:H2	1.27	0.43
1:B:517:LEU:HD23	1:B:517:LEU:HA	1.88	0.42
1:B:661:TYR:HB3	1:B:669:ARG:HG2	2.00	0.42
1:C:663:GLU:CB	1:C:668:VAL:HG21	2.49	0.42
1:B:663:GLU:CB	1:B:668:VAL:HG21	2.49	0.42
1:A:437:SER:OG	1:A:438:HIS:ND1	2.32	0.42
1:B:611:ARG:HH12	1:B:631:THR:HG22	1.85	0.42
1:B:196:VAL:CG2	1:B:197:PRO:HD2	2.50	0.42
1:C:661:TYR:OH	1:C:663:GLU:OE2	2.26	0.42
1:A:223:ASN:O	1:C:735:VAL:HG12	2.18	0.42
1:A:309:LEU:HB2	1:A:310:ARG:HH11	1.84	0.42
1:A:439:VAL:CG2	1:A:461:LEU:CD1	2.85	0.42
1:A:512:VAL:CG2	1:B:700:VAL:HG22	2.44	0.42
1:A:196:VAL:CG2	1:A:197:PRO:HD2	2.50	0.42

Continued on next page...

PRELIMINARY VALIDATION REPORT

Continued from previous page...

Atom-1	Atom-2	Interatomic distance (Å)	Clash overlap (Å)
1:A:416:GLN:N	1:A:416:GLN:OE1	2.48	0.42
1:A:713:ASP:O	1:A:717:ILE:HG12	2.20	0.42
1:B:254:HIS:HA	1:B:277:CYS:O	2.20	0.42
2:F:3:MAN:H62	2:F:4:MAN:H2	1.38	0.42
1:A:663:GLU:CB	1:A:668:VAL:HG21	2.49	0.42
1:A:679:ILE:CG2	1:B:131:VAL:HG21	2.48	0.42
1:B:702:THR:HG22	1:B:703:ARG:N	2.35	0.42
1:C:254:HIS:HA	1:C:277:CYS:O	2.20	0.42
2:D:3:MAN:H62	2:D:4:MAN:H2	1.35	0.42
1:A:185:TYR:HB2	1:C:227:GLU:HG2	2.02	0.42
1:B:380:VAL:HG12	1:B:393:MET:HG2	2.02	0.42
1:B:679:ILE:CG2	1:C:131:VAL:HG21	2.46	0.42
1:C:196:VAL:CG2	1:C:197:PRO:HD2	2.50	0.42
1:C:702:THR:HG22	1:C:703:ARG:N	2.35	0.41
1:C:713:ASP:O	1:C:717:ILE:HG12	2.20	0.41
1:A:546:GLU:HG2	1:A:550:TRP:HD1	1.85	0.41
1:B:309:LEU:HB2	1:B:310:ARG:HH11	1.84	0.41
1:B:713:ASP:O	1:B:717:ILE:HG12	2.20	0.41
1:C:437:SER:OG	1:C:438:HIS:ND1	2.32	0.41
1:A:382:ARG:NH1	1:A:447:TYR:OH	2.54	0.41
1:B:578:VAL:CG1	1:B:579:ILE:N	2.84	0.41
1:C:382:ARG:NH1	1:C:447:TYR:OH	2.54	0.41
1:C:578:VAL:CG1	1:C:579:ILE:N	2.84	0.41
1:C:611:ARG:HH12	1:C:631:THR:HG22	1.85	0.41
1:A:196:VAL:HG22	1:A:197:PRO:HD2	2.03	0.41
1:A:702:THR:HG22	1:A:703:ARG:N	2.35	0.41
1:A:190:ASN:ND2	1:B:190:ASN:OD1	2.38	0.41
1:B:139:CYS:HB3	1:B:540:CYS:HB2	2.00	0.41
1:B:660:VAL:CG1	1:B:670:GLU:HG3	2.50	0.41
1:B:244:LYS:HA	2:E:2:NAG:O3	2.20	0.41
1:C:439:VAL:CG2	1:C:461:LEU:CD1	2.85	0.41
1:B:433:ARG:HB3	1:B:434:TYR:CD1	2.56	0.41
1:B:546:GLU:HG2	1:B:550:TRP:HD1	1.86	0.41
1:C:433:ARG:HB3	1:C:434:TYR:CD1	2.56	0.41
1:B:123:PRO:HD2	1:B:585:PRO:HD2	2.03	0.41
1:C:546:GLU:HG2	1:C:550:TRP:HD1	1.85	0.41
1:A:123:PRO:HD2	1:A:585:PRO:HD2	2.03	0.41
1:B:513:GLU:C	1:B:515:ALA:N	2.73	0.41
1:B:592:ARG:NE	1:B:594:ILE:HD11	2.31	0.41
1:B:513:GLU:HB3	1:C:513:GLU:OE2	2.21	0.41
1:B:382:ARG:NH1	1:B:447:TYR:OH	2.54	0.41

Continued on next page...

PRELIMINARY VALIDATION REPORT

Continued from previous page...

Atom-1	Atom-2	Interatomic distance (Å)	Clash overlap (Å)
1:A:254:HIS:HA	1:A:277:CYS:O	2.20	0.41
1:A:380:VAL:HG12	1:A:393:MET:HG2	2.02	0.41
1:C:196:VAL:HG22	1:C:197:PRO:HD2	2.03	0.41
1:C:309:LEU:HB2	1:C:310:ARG:HH11	1.84	0.41
1:A:514:PHE:CE1	1:C:697:PRO:HB3	2.56	0.40
1:A:587:LEU:HD13	1:A:635:LEU:HD12	2.04	0.40
1:A:678:MET:CE	1:B:132:ARG:HG3	2.50	0.40
1:B:196:VAL:HG22	1:B:197:PRO:HD2	2.03	0.40
1:A:433:ARG:HB3	1:A:434:TYR:CD1	2.56	0.40
1:C:229:PHE:HE2	1:C:235:PRO:HG3	1.87	0.40
1:C:123:PRO:HD2	1:C:585:PRO:HD2	2.03	0.40
2:F:2:NAG:N2	2:F:2:NAG:H5	2.36	0.40
1:A:611:ARG:HH12	1:A:631:THR:HG22	1.85	0.40
1:B:229:PHE:HE2	1:B:235:PRO:HG3	1.86	0.40
1:C:517:LEU:HD23	1:C:517:LEU:HA	1.88	0.40
1:C:609:TYR:HA	1:C:642:LEU:HD13	2.04	0.40
1:A:578:VAL:CG1	1:A:579:ILE:N	2.84	0.40
1:B:130:ILE:HD12	1:B:578:VAL:HG11	2.03	0.40
1:C:130:ILE:HD12	1:C:578:VAL:HG11	2.03	0.40

There are no symmetry-related clashes.

5.3 Torsion angles ⓘ

5.3.1 Protein backbone ⓘ

In the following table, the Percentiles column shows the percent Ramachandran outliers of the chain as a percentile score with respect to all PDB entries followed by that with respect to all EM entries.

The Analysed column shows the number of residues for which the backbone conformation was analysed, and the total number of residues.

Mol	Chain	Analysed	Favoured	Allowed	Outliers	Percentiles	
1	A	580/931 (62%)	569 (98%)	11 (2%)	0	100	100
1	B	580/931 (62%)	567 (98%)	13 (2%)	0	100	100
1	C	580/931 (62%)	567 (98%)	13 (2%)	0	100	100
All	All	1740/2793 (62%)	1703 (98%)	37 (2%)	0	100	100

There are no Ramachandran outliers to report.

PRELIMINARY VALIDATION REPORT

5.3.2 Protein sidechains (i)

In the following table, the Percentiles column shows the percent sidechain outliers of the chain as a percentile score with respect to all PDB entries followed by that with respect to all EM entries.

The Analysed column shows the number of residues for which the sidechain conformation was analysed, and the total number of residues.

Mol	Chain	Analysed	Rotameric	Outliers	Percentiles	
1	A	524/815 (64%)	522 (100%)	2 (0%)	91	94
1	B	524/815 (64%)	522 (100%)	2 (0%)	91	94
1	C	524/815 (64%)	522 (100%)	2 (0%)	91	94
All	All	1572/2445 (64%)	1566 (100%)	6 (0%)	91	94

All (6) residues with a non-rotameric sidechain are listed below:

Mol	Chain	Res	Type
1	A	139	CYS
1	A	608	CYS
1	B	139	CYS
1	B	608	CYS
1	C	139	CYS
1	C	608	CYS

Some sidechains can be flipped to improve hydrogen bonding and reduce clashes. All (16) such sidechains are listed below:

Mol	Chain	Res	Type
1	A	223	ASN
1	A	525	GLN
1	A	527	HIS
1	A	543	GLN
1	A	628	GLN
1	B	223	ASN
1	B	525	GLN
1	B	527	HIS
1	B	543	GLN
1	B	628	GLN
1	C	223	ASN
1	C	411	GLN
1	C	525	GLN
1	C	527	HIS
1	C	543	GLN

Continued on next page...

PRELIMINARY VALIDATION REPORT

Continued from previous page...

Mol	Chain	Res	Type
1	C	628	GLN

5.3.3 RNA (i)

There are no RNA molecules in this entry.

5.4 Non-standard residues in protein, DNA, RNA chains (i)

There are no non-standard protein/DNA/RNA residues in this entry.

5.5 Carbohydrates (i)

18 monosaccharides are modelled in this entry.

In the following table, the Counts columns list the number of bonds (or angles) for which Mogul statistics could be retrieved, the number of bonds (or angles) that are observed in the model and the number of bonds (or angles) that are defined in the Chemical Component Dictionary. The Link column lists molecule types, if any, to which the group is linked. The Z score for a bond length (or angle) is the number of standard deviations the observed value is removed from the expected value. A bond length (or angle) with $|Z| > 2$ is considered an outlier worth inspection. RMSZ is the root-mean-square of all Z scores of the bond lengths (or angles).

Mol	Type	Chain	Res	Link	Bond lengths			Bond angles		
					Counts	RMSZ	$\#\|Z\| > 2$	Counts	RMSZ	$\#\|Z\| > 2$
2	NAG	D	1	1,2	14,14,15	0.31	0	17,19,21	0.60	0
2	NAG	D	2	2	14,14,15	0.29	0	17,19,21	0.71	1 (5%)
2	MAN	D	3	2	11,11,12	0.23	0	15,15,17	0.70	0
2	MAN	D	4	2	11,11,12	0.27	0	15,15,17	0.69	0
2	MAN	D	5	2	11,11,12	0.26	0	15,15,17	0.62	0
2	MAN	D	6	2	11,11,12	0.26	0	15,15,17	0.63	0
2	NAG	E	1	1,2	14,14,15	0.31	0	17,19,21	0.62	0
2	NAG	E	2	2	14,14,15	0.29	0	17,19,21	0.73	1 (5%)
2	MAN	E	3	2	11,11,12	0.22	0	15,15,17	0.68	0
2	MAN	E	4	2	11,11,12	0.32	0	15,15,17	0.92	1 (6%)
2	MAN	E	5	2	11,11,12	0.26	0	15,15,17	0.63	0
2	MAN	E	6	2	11,11,12	0.26	0	15,15,17	0.63	0
2	NAG	F	1	1,2	14,14,15	0.31	0	17,19,21	0.62	0
2	NAG	F	2	2	14,14,15	0.30	0	17,19,21	0.71	1 (5%)
2	MAN	F	3	2	11,11,12	0.22	0	15,15,17	0.71	0
2	MAN	F	4	2	11,11,12	0.27	0	15,15,17	0.72	0
2	MAN	F	5	2	11,11,12	0.26	0	15,15,17	0.66	0

PRELIMINARY VALIDATION REPORT

Mol	Type	Chain	Res	Link	Bond lengths			Bond angles		
					Counts	RMSZ	#|Z| > 2	Counts	RMSZ	#|Z| > 2
2	MAN	F	6	2	11,11,12	0.26	0	15,15,17	0.63	0

In the following table, the Chirals column lists the number of chiral outliers, the number of chiral centers analysed, the number of these observed in the model and the number defined in the Chemical Component Dictionary. Similar counts are reported in the Torsion and Rings columns. '-' means no outliers of that kind were identified.

Mol	Type	Chain	Res	Link	Chirals	Torsions	Rings
2	NAG	D	1	1,2	-	0/6/23/26	0/1/1/1
2	NAG	D	2	2	-	2/6/23/26	0/1/1/1
2	MAN	D	3	2	-	1/2/19/22	0/1/1/1
2	MAN	D	4	2	-	2/2/19/22	0/1/1/1
2	MAN	D	5	2	-	1/2/19/22	0/1/1/1
2	MAN	D	6	2	-	2/2/19/22	0/1/1/1
2	NAG	E	1	1,2	-	0/6/23/26	0/1/1/1
2	NAG	E	2	2	-	2/6/23/26	0/1/1/1
2	MAN	E	3	2	-	0/2/19/22	0/1/1/1
2	MAN	E	4	2	-	1/2/19/22	0/1/1/1
2	MAN	E	5	2	-	2/2/19/22	0/1/1/1
2	MAN	E	6	2	-	2/2/19/22	0/1/1/1
2	NAG	F	1	1,2	-	0/6/23/26	0/1/1/1
2	NAG	F	2	2	-	2/6/23/26	0/1/1/1
2	MAN	F	3	2	-	1/2/19/22	0/1/1/1
2	MAN	F	4	2	-	2/2/19/22	0/1/1/1
2	MAN	F	5	2	-	1/2/19/22	0/1/1/1
2	MAN	F	6	2	-	2/2/19/22	0/1/1/1

There are no bond length outliers.

All (4) bond angle outliers are listed below:

Mol	Chain	Res	Type	Atoms	Z	Observed(^{o})	Ideal(^{o})
2	E	4	MAN	C1-C2-C3	2.84	113.16	109.67
2	E	2	NAG	C1-O5-C5	2.29	115.30	112.19
2	F	2	NAG	C1-O5-C5	2.26	115.25	112.19
2	D	2	NAG	C1-O5-C5	2.22	115.20	112.19

There are no chirality outliers.

All (23) torsion outliers are listed below:

PRELIMINARY VALIDATION REPORT

Mol	Chain	Res	Type	Atoms
2	E	2	NAG	C8-C7-N2-C2
2	E	2	NAG	O7-C7-N2-C2
2	F	2	NAG	C8-C7-N2-C2
2	F	2	NAG	O7-C7-N2-C2
2	E	6	MAN	O5-C5-C6-O6
2	D	4	MAN	O5-C5-C6-O6
2	D	6	MAN	O5-C5-C6-O6
2	F	4	MAN	O5-C5-C6-O6
2	F	6	MAN	O5-C5-C6-O6
2	E	5	MAN	O5-C5-C6-O6
2	D	6	MAN	C4-C5-C6-O6
2	E	6	MAN	C4-C5-C6-O6
2	D	4	MAN	C4-C5-C6-O6
2	F	4	MAN	C4-C5-C6-O6
2	F	6	MAN	C4-C5-C6-O6
2	D	2	NAG	C8-C7-N2-C2
2	F	5	MAN	O5-C5-C6-O6
2	D	2	NAG	O7-C7-N2-C2
2	E	5	MAN	C4-C5-C6-O6
2	D	5	MAN	O5-C5-C6-O6
2	E	4	MAN	C4-C5-C6-O6
2	F	3	MAN	C4-C5-C6-O6
2	D	3	MAN	C4-C5-C6-O6

There are no ring outliers.

12 monomers are involved in 23 short contacts:

Mol	Chain	Res	Type	Clashes	Symm-Clashes
2	E	2	NAG	3	0
2	D	3	MAN	2	0
2	F	4	MAN	1	0
2	F	3	MAN	2	0
2	F	2	NAG	4	0
2	E	3	MAN	2	0
2	D	2	NAG	4	0
2	E	4	MAN	1	0
2	E	1	NAG	3	0
2	F	1	NAG	3	0
2	D	1	NAG	3	0
2	D	4	MAN	1	0

The following is a two-dimensional graphical depiction of Mogul quality analysis of bond lengths, bond angles, torsion angles, and ring geometry for oligosaccharide.

PRELIMINARY VALIDATION REPORT

Oligosaccharide Chain D	
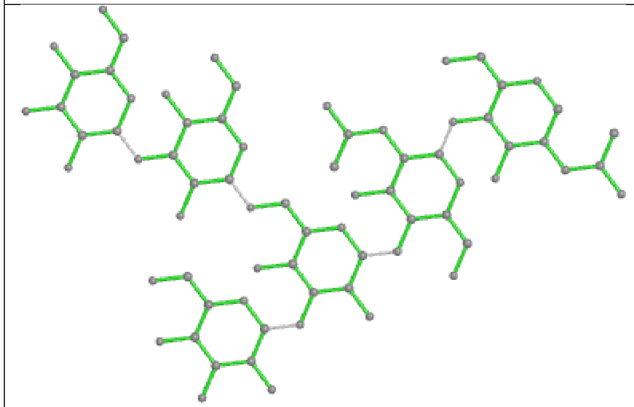 Bond lengths	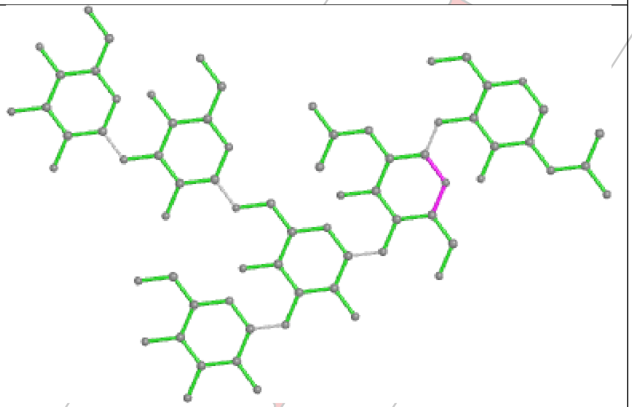 Bond angles
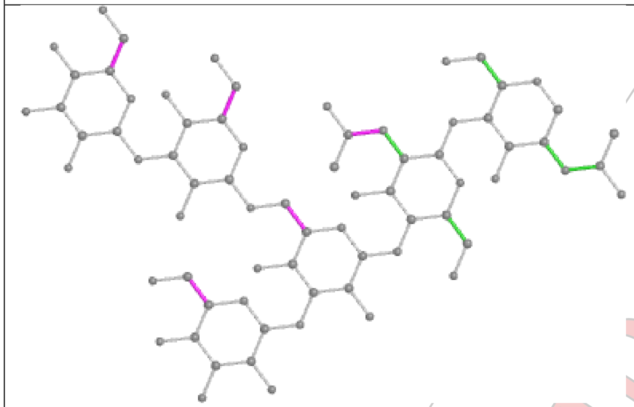 Torsions	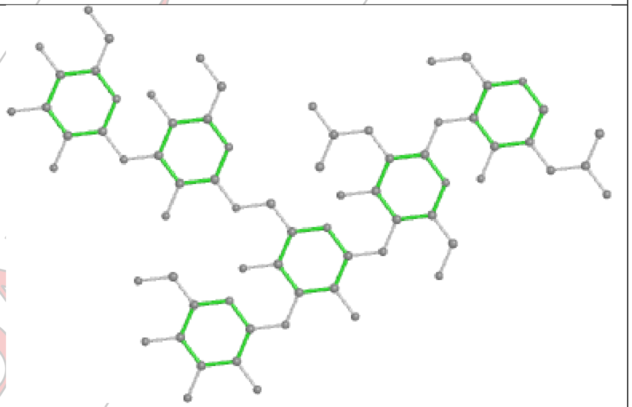 Rings

Oligosaccharide Chain E

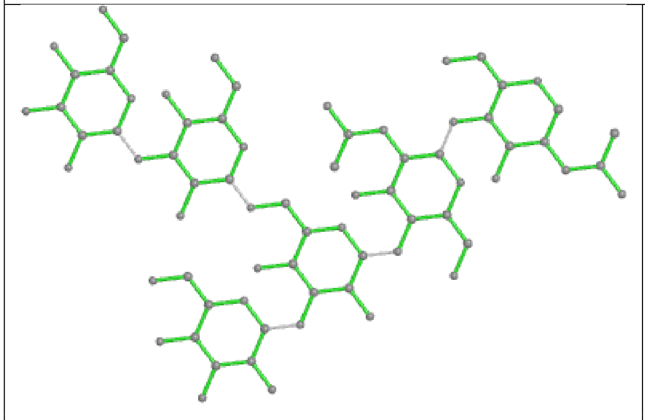

Bond lengths

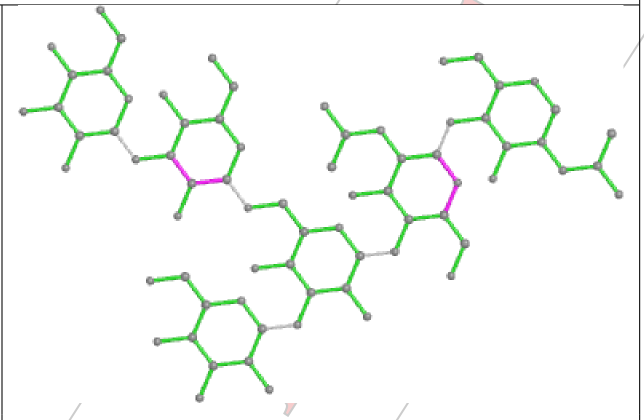

Bond angles

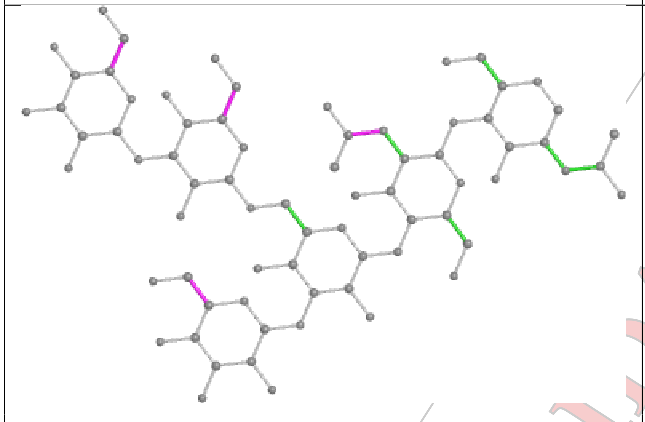

Torsions

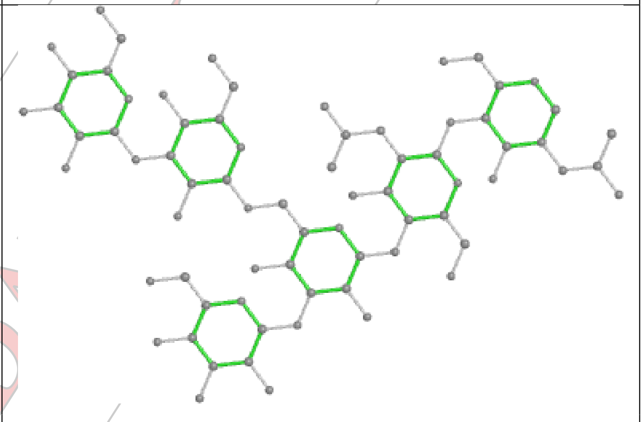

Rings

PRELIMINARY

Oligosaccharide Chain F

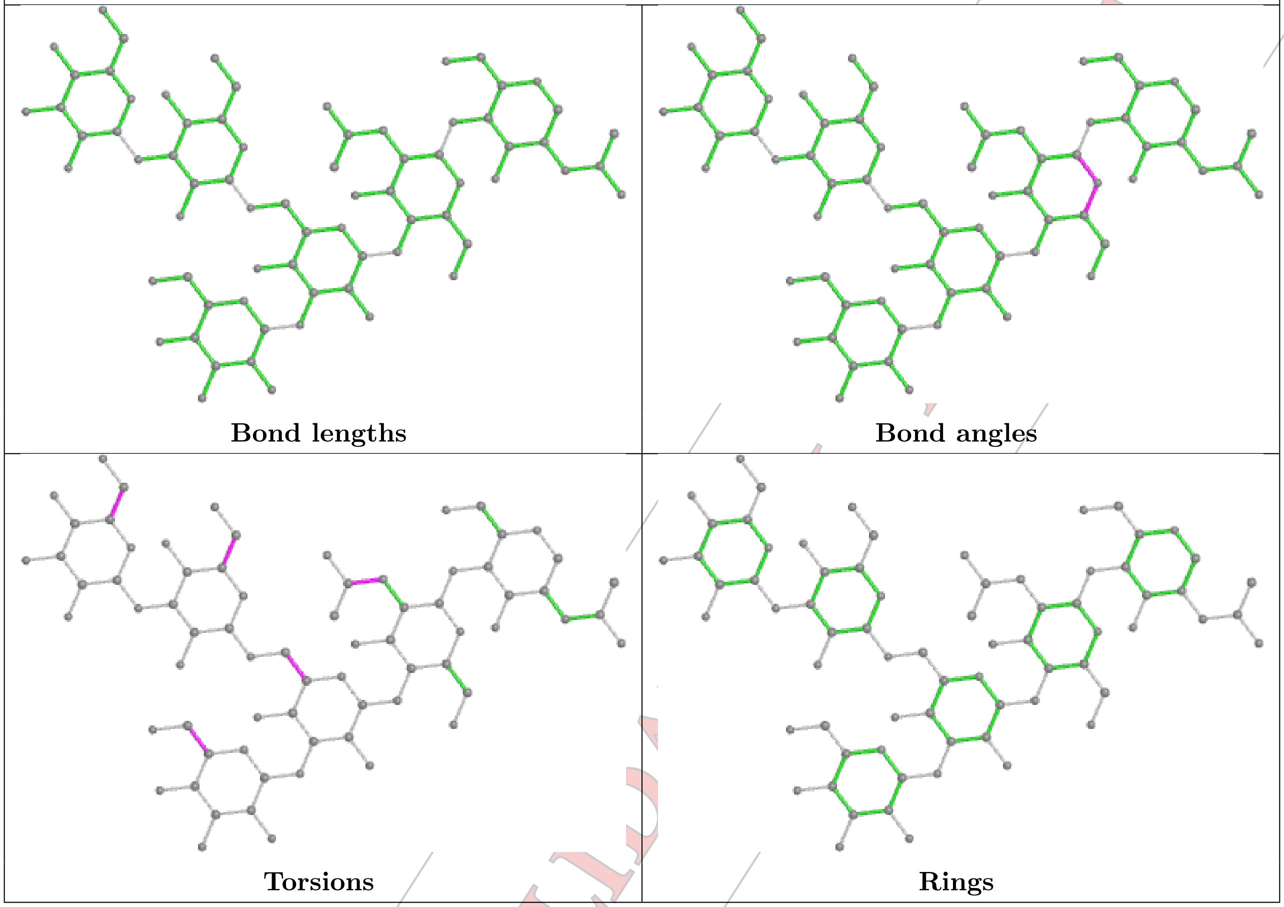

5.6 Ligand geometry (i)

3 ligands are modelled in this entry.

In the following table, the Counts columns list the number of bonds (or angles) for which Mogul statistics could be retrieved, the number of bonds (or angles) that are observed in the model and the number of bonds (or angles) that are defined in the Chemical Component Dictionary. The Link column lists molecule types, if any, to which the group is linked. The Z score for a bond length (or angle) is the number of standard deviations the observed value is removed from the expected value. A bond length (or angle) with $|Z| > 2$ is considered an outlier worth inspection. RMSZ is the root-mean-square of all Z scores of the bond lengths (or angles).

Mol	Type	Chain	Res	Link	Bond lengths			Bond angles		
					Counts	RMSZ	$\#\|Z\| > 2$	Counts	RMSZ	$\#\|Z\| > 2$
3	NAG	B	900	1	14,14,15	0.25	0	17,19,21	0.44	0
3	NAG	A	900	1	14,14,15	0.27	0	17,19,21	0.45	0
3	NAG	C	900	1	14,14,15	0.26	0	17,19,21	0.46	0

In the following table, the Chirals column lists the number of chiral outliers, the number of chiral centers analysed, the number of these observed in the model and the number defined in the Chemical Component Dictionary. Similar counts are reported in the Torsion and Rings columns.

'-' means no outliers of that kind were identified.

Mol	Type	Chain	Res	Link	Chirals	Torsions	Rings
3	NAG	B	900	1	-	2/6/23/26	0/1/1/1
3	NAG	A	900	1	-	2/6/23/26	0/1/1/1
3	NAG	C	900	1	-	2/6/23/26	0/1/1/1

There are no bond length outliers.

There are no bond angle outliers.

There are no chirality outliers.

All (6) torsion outliers are listed below:

Mol	Chain	Res	Type	Atoms
3	B	900	NAG	C4-C5-C6-O6
3	A	900	NAG	C4-C5-C6-O6
3	C	900	NAG	C4-C5-C6-O6
3	B	900	NAG	O5-C5-C6-O6
3	A	900	NAG	O5-C5-C6-O6
3	C	900	NAG	O5-C5-C6-O6

There are no ring outliers.

No monomer is involved in short contacts.

5.7 Other polymers (i)

There are no such residues in this entry.

5.8 Polymer linkage issues (i)

There are no chain breaks in this entry.

PRELIMINARY VALIDATION REPORT

6 Map visualisation (i)

This section contains visualisations of the EMDB entry D_1000251554. These are intended to permit visual inspection of the internal detail of the map and identification of artifacts.

6.1 Orthogonal projections (i)

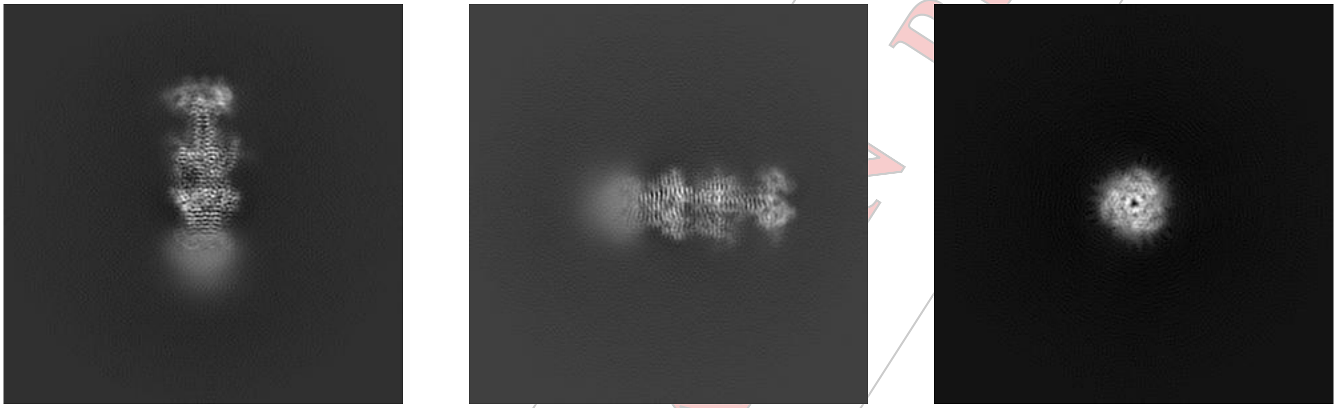

X Y Z

The images above show the map projected in three orthogonal projections, in greyscale.

6.2 Central slices (i)

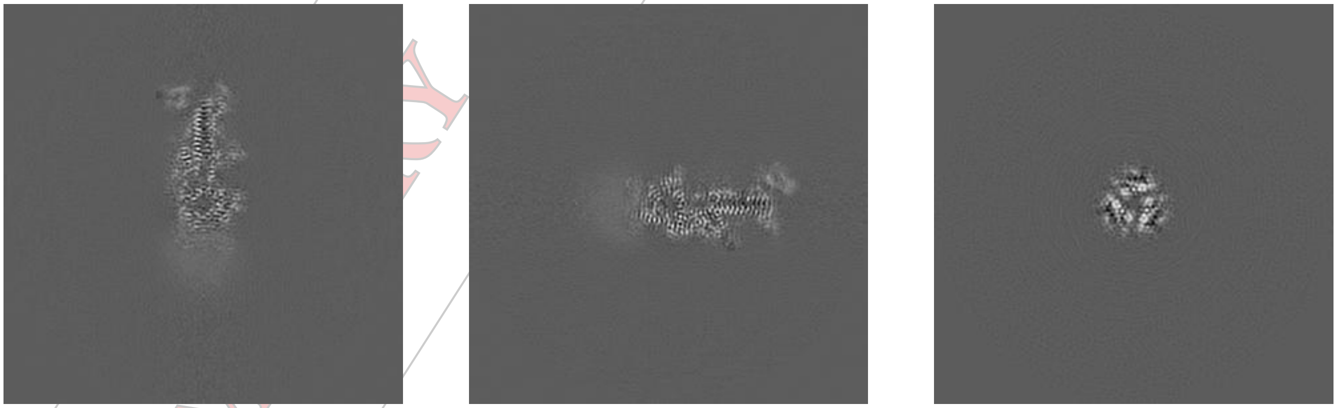

X Y Z

The images above show central slices of the map in three orthogonal directions, in greyscale.

PRELIMINARY VALIDATION REPORT

6.3 Largest variance slices (i)

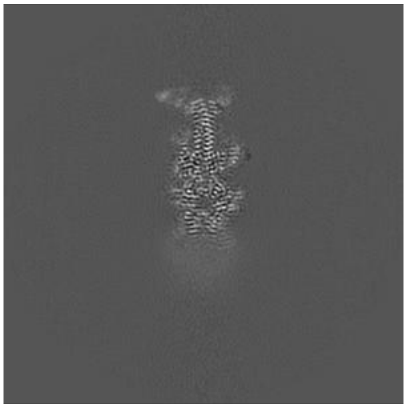

X Index: 205

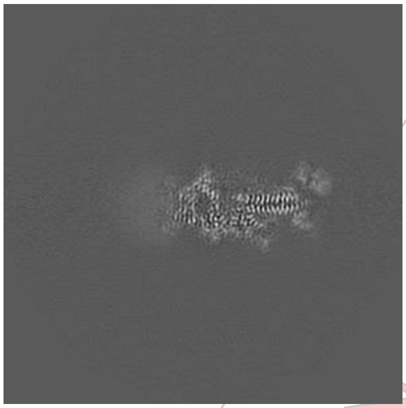

Y Index: 203

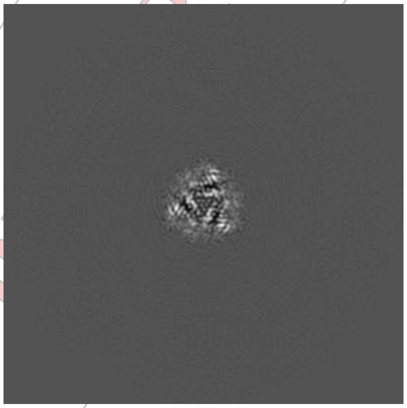

Z Index: 207

The images above show the highest variance slices of the map in three orthogonal directions, in greyscale.

6.4 Orthogonal surface views (i)

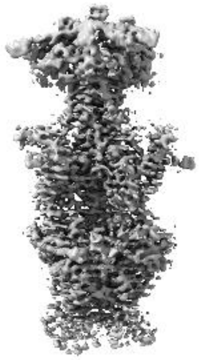

X

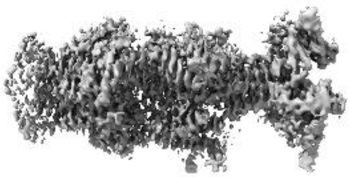

Y

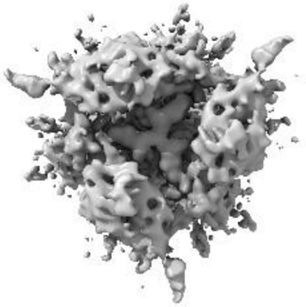

Z

The images above show the 3D surface view of the map at the recommended contour level 0.02. This in conjunction with the slice images can indicate whether an appropriate contour level has been selected.

6.5 Mask visualisation (i)

This section was not generated. No masks were provided.

7 Map analysis (i)

This section contains the results of statistical analysis of the map.

7.1 Map-value distribution (i)

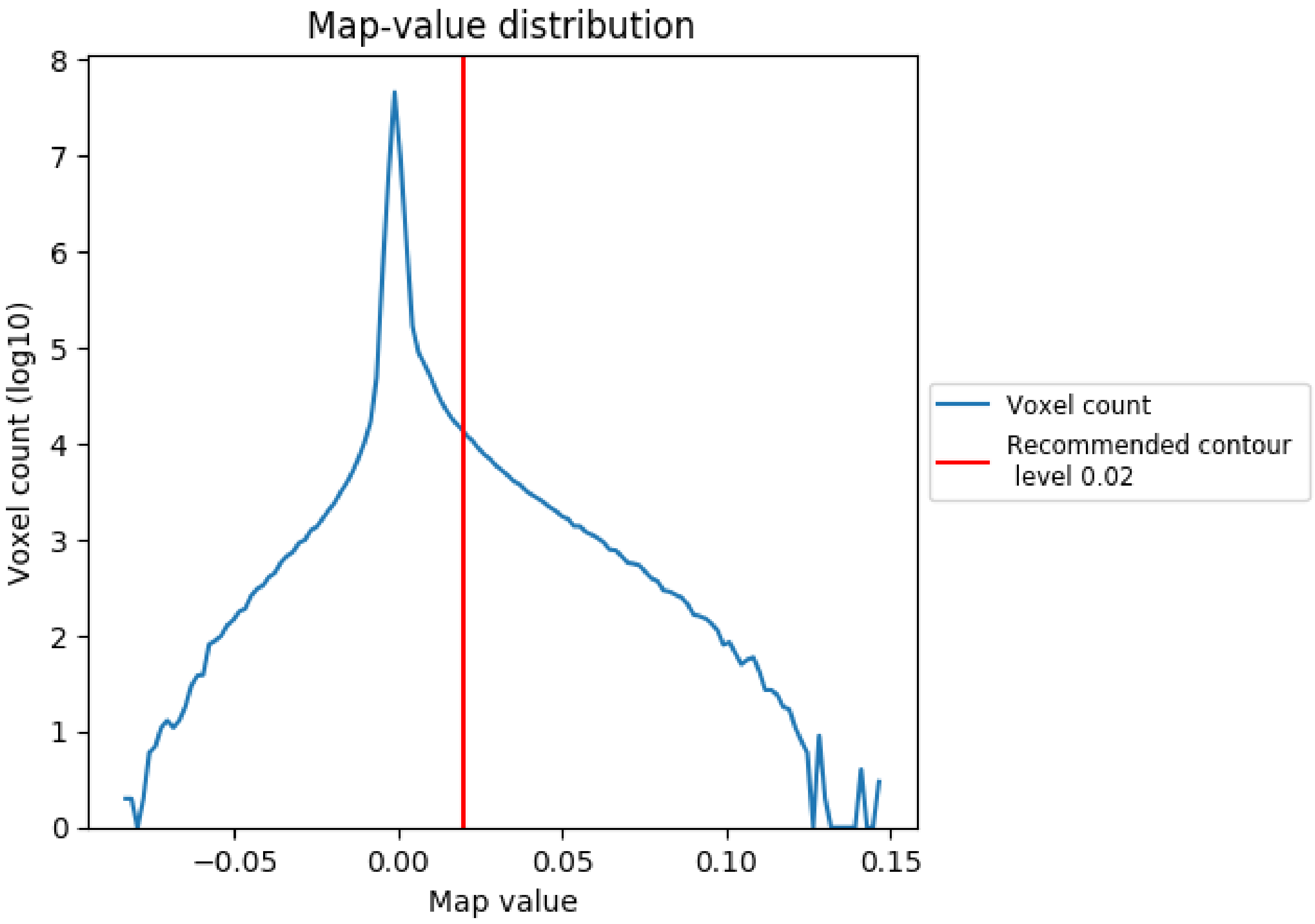

The map-value distribution is plotted in 128 intervals along the x-axis. The y-axis is logarithmic. A spike in this graph at zero usually indicates that the volume has been masked.

PRELIMINARY REPORT

7.2 Volume estimate (i)

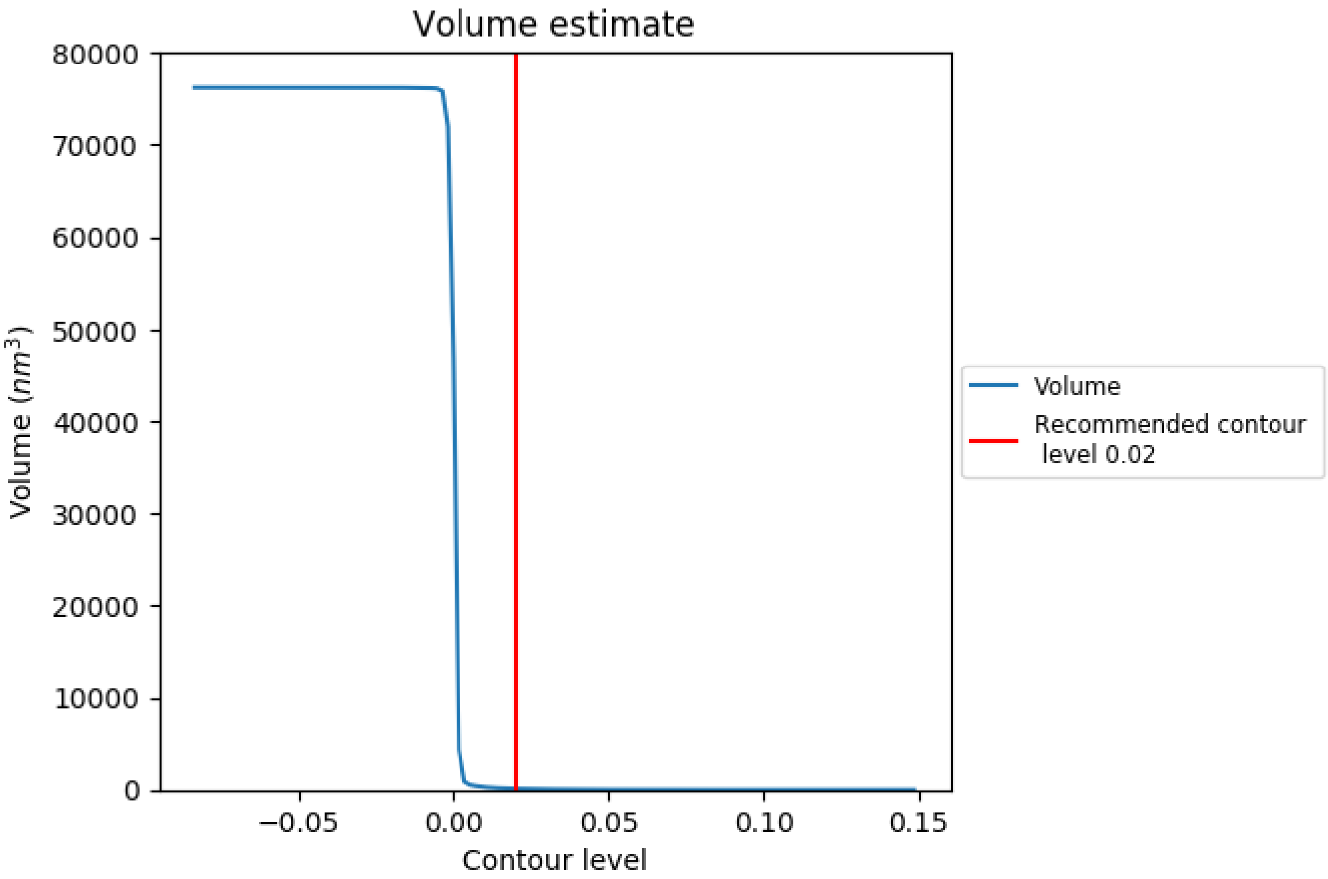

The volume at the recommended contour level is 129 nm^3; this corresponds to an approximate mass of 117 kDa.

The volume estimate graph shows how the enclosed volume varies with the contour level. The recommended contour level is shown as a vertical line and the intersection between the line and the curve gives the volume of the enclosed surface at the given level.

PRELIMINARY VA

7.3 Rotationally averaged power spectrum (i)

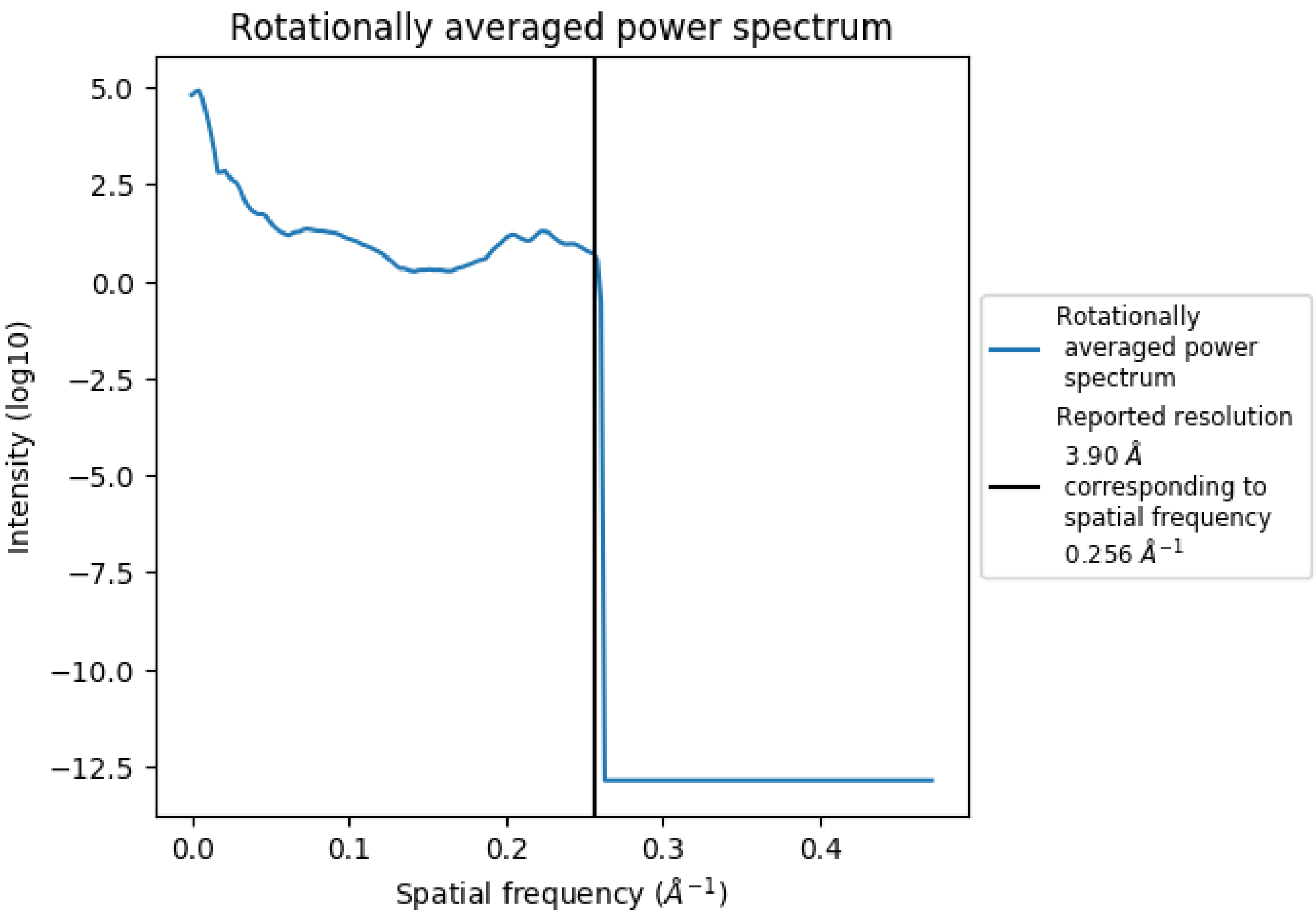

PRELIMINARY

8 Fourier-Shell correlation (i)

Fourier-Shell Correlation (FSC) is the most commonly used method to estimate the resolution for single-particle and subtomogram-averaging methods. The shape of the curve depends on the imposed symmetry, mask and whether or not the two 3D reconstructions used were processed from a common reference. The reported resolution is shown as a black line. Curves are displayed for 3σ, 1-bit and 1/2-bit in addition to lines showing the 0.143 gold standard cut-off, 0.333 cut-off and legacy 0.5 cut-off.

8.1 Resolution estimates (i)

These are global values for the map.

Source	Criterion	Resolution estimate (Å)
Reported value	Not provided	3.90
Author-provided FSC	FSC 0.5 CUT-OFF	4.11
Author-provided FSC	FSC 1 BIT CUT-OFF	3.96
Author-provided FSC	FSC 0.33 CUT-OFF	3.96
Author-provided FSC	FSC 1/2 BIT CUT-OFF	3.87
Author-provided FSC	FSC 0.143 CUT-OFF	3.86
Author-provided FSC	FSC 3 SIGMA CUT-OFF	3.79

8.2 Calculated FSC (i)

This section was not generated. Half-maps were not provided.

PRELIMINARY VALIDATION REPORT

8.3 Author-provided FSC ⓘ

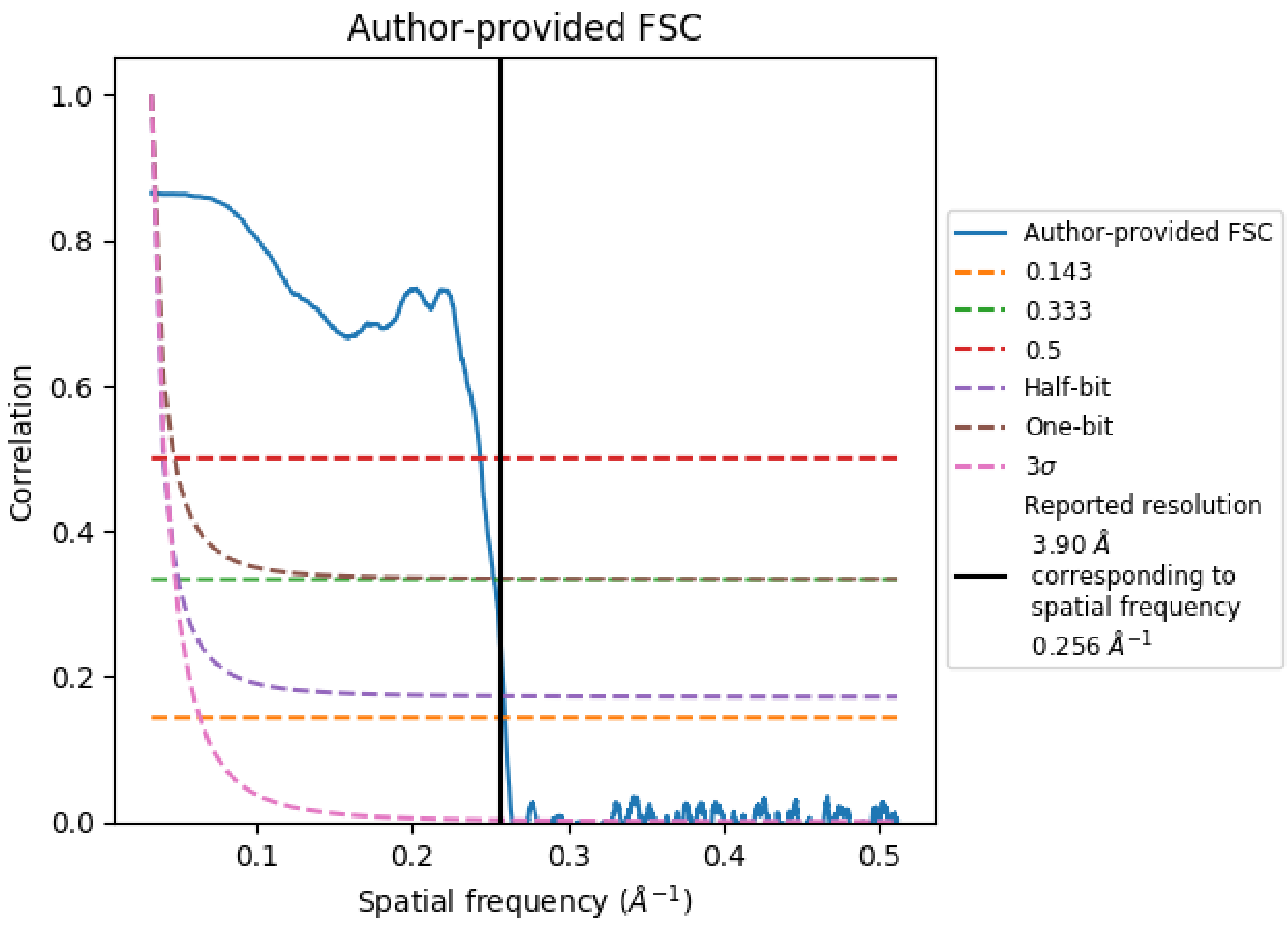

This FSC information was provided by the depositor.

PRELIMINARY

9 Map-model fit (i)

This section contains information regarding the fit between EMDB map D_1000251554 and PDB model D_1000251554. Per-residue inclusion information can be found in section 3 on page 5.

9.1 Map-model overlay (i)

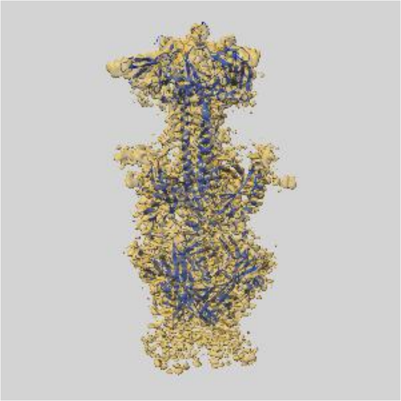

X

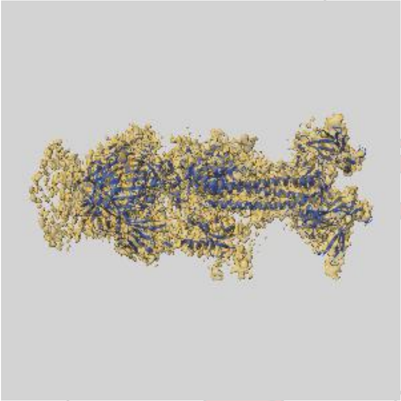

Y

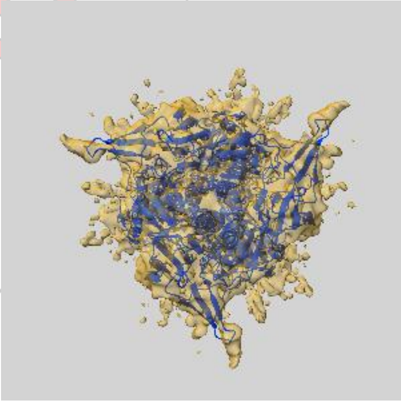

Z

The images above show the 3D surface view of the map at the recommended contour level 0.02 at 50% transparency in yellow overlaid with a ribbon representation of the model coloured in blue. These images allow for the visual assessment of the quality of fit between the atomic model and the map.

PRELIMINARY VALIDATION REPORT

9.2 Atom inclusion (i)

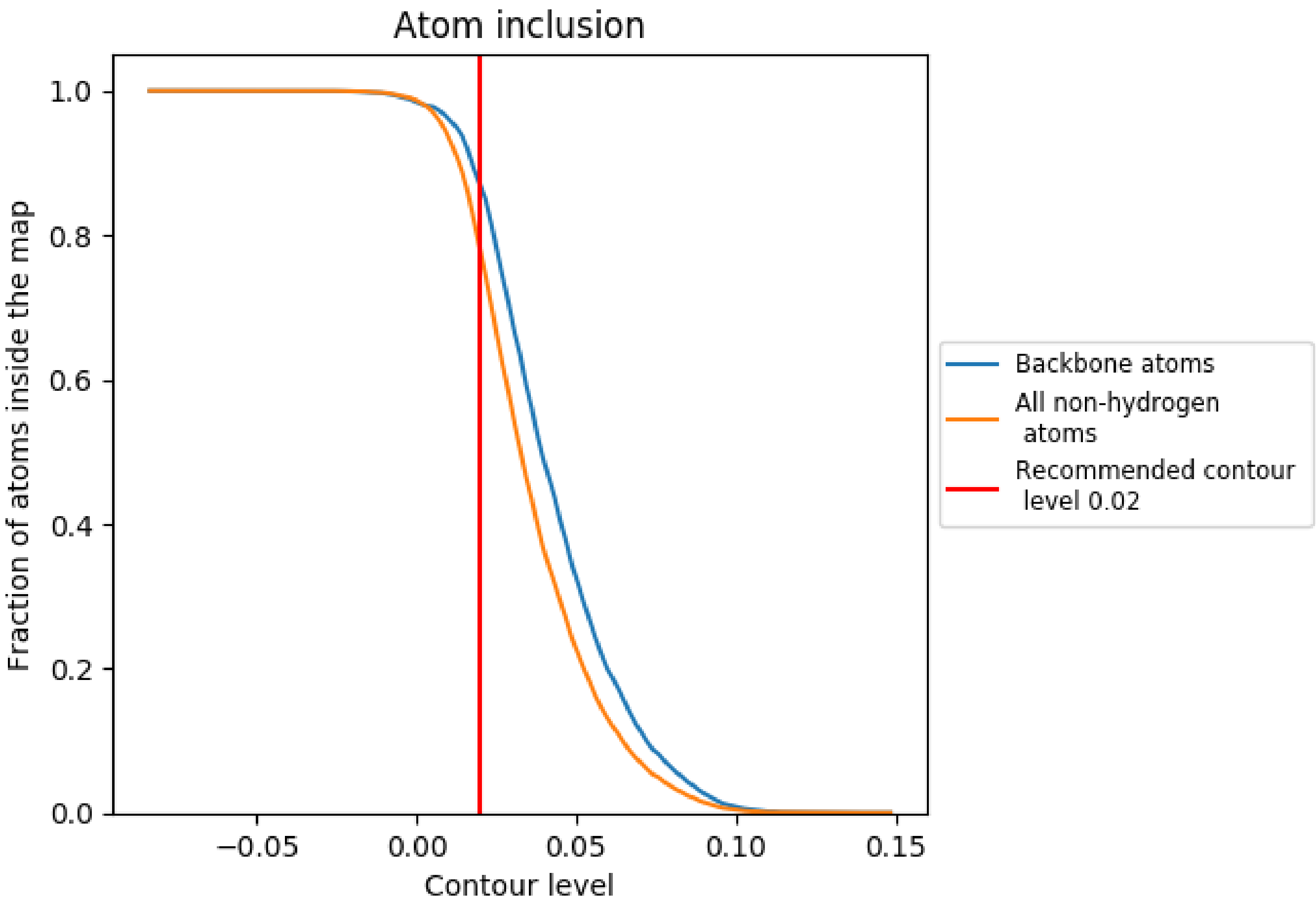

At the recommended contour level, 87% of all backbone atoms, 78% of all non-hydrogen atoms, are inside the map.

PRELIMINARY
