# Supplementary material for: The N-terminus of varicella-zoster virus glycoprotein B has a functional role in fusion
Source: PLoS Pathog. 2021 Jan 7;17(1):e1008961. doi: 10.1371/journal.ppat.1008961 (PMC7817050; doi:10.1371/journal.ppat.1008961)
Supplement: S4 Validation Report — (PDF) [file ppat.1008961.s022.pdf]

Full wwPDB X-ray Structure Validation Report (i)

Aug 26, 2020 – 01:42 PM EDT

PDB ID	:	6VLK
Title	:	A varicella-zoster virus glycoprotein
Authors	:	Xing, Y.
Deposited on	:	2020-01-24
Resolution	:	2.45 Å(reported)

This is a Full wwPDB X-ray Structure Validation Report for a publicly released PDB entry.

We welcome your comments at *validation@mail.wwpdb.org*
A user guide is available at
https://www.wwpdb.org/validation/2017/XrayValidationReportHelp
with specific help available everywhere you see the (i) symbol.

The following versions of software and data (see references (i)) were used in the production of this report:

MolProbity	:	4.02b-467
Mogul	:	1.8.5 (274361), CSD as541be (2020)
Xtriage (Phenix)	:	1.13
EDS	:	2.13.1
buster-report	:	1.1.7 (2018)
Percentile statistics	:	20191225.v01 (using entries in the PDB archive December 25th 2019)
Refmac	:	5.8.0158
CCP4	:	7.0.044 (Gargrove)
Ideal geometry (proteins)	:	Engh & Huber (2001)
Ideal geometry (DNA, RNA)	:	Parkinson et al. (1996)
Validation Pipeline (wwPDB-VP)	:	2.13.1

1 Overall quality at a glance (i)

The following experimental techniques were used to determine the structure:
X-RAY DIFFRACTION

The reported resolution of this entry is 2.45 Å.

Percentile scores (ranging between 0-100) for global validation metrics of the entry are shown in the following graphic. The table shows the number of entries on which the scores are based.

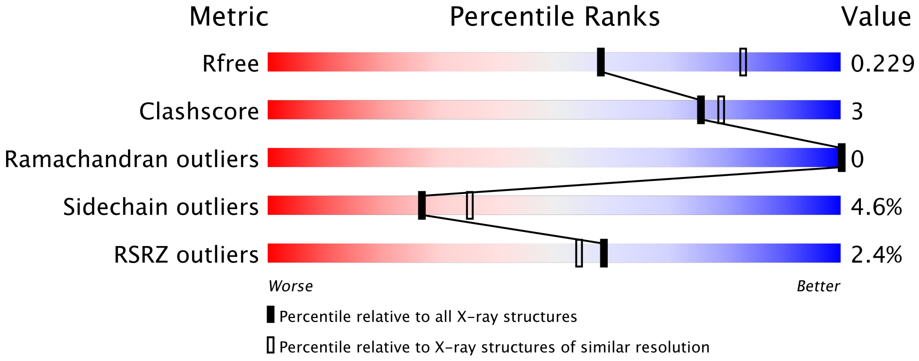

Metric	Whole archive (#Entries)	Similar resolution (#Entries, resolution range(Å))
R_{free}	130704	1544 (2.48-2.44)
Clashscore	141614	1613 (2.48-2.44)
Ramachandran outliers	138981	1598 (2.48-2.44)
Sidechain outliers	138945	1598 (2.48-2.44)
RSRZ outliers	127900	1523 (2.48-2.44)

The table below summarises the geometric issues observed across the polymeric chains and their fit to the electron density. The red, orange, yellow and green segments on the lower bar indicate the fraction of residues that contain outliers for >=3, 2, 1 and 0 types of geometric quality criteria respectively. A grey segment represents the fraction of residues that are not modelled. The numeric value for each fraction is indicated below the corresponding segment, with a dot representing fractions <=5% The upper red bar (where present) indicates the fraction of residues that have poor fit to the electron density. The numeric value is given above the bar.

Mol	Chain	Length	Quality of chain
1	A	744	% / 70% 7% • 22%
1	B	744	3% / 69% 9% • 22%
2	C	7	29% 71%
3	D	5	60% 40%
4	E	2	100%

The following table lists non-polymeric compounds, carbohydrate monomers and non-standard residues in protein, DNA, RNA chains that are outliers for geometric or electron-density-fit criteria:

Mol	Type	Chain	Res	Chirality	Geometry	Clashes	Electron density
3	NAG	D	2	-	-	-	X
3	BMA	D	3	-	-	-	X
3	MAN	D	4	-	-	-	X
3	MAN	D	5	-	-	-	X

2 Entry composition (i)

There are 6 unique types of molecules in this entry. The entry contains 9766 atoms, of which 0 are hydrogens and 0 are deuteriums.

In the tables below, the ZeroOcc column contains the number of atoms modelled with zero occupancy, the AltConf column contains the number of residues with at least one atom in alternate conformation and the Trace column contains the number of residues modelled with at most 2 atoms.

- Molecule 1 is a protein called Envelope glycoprotein B.

Mol	Chain	Residues	Atoms					ZeroOcc	AltConf	Trace
1	A	584	Total 4693	C 2961	N 821	O 889	S 22	0	0	0
1	B	583	Total 4688	C 2958	N 820	O 888	S 22	0	0	0

There are 26 discrepancies between the modelled and reference sequences:

Chain	Residue	Modelled	Actual	Comment	Reference
A	180	GLY	TRP	engineered mutation	UNP Q4JR05
A	185	GLY	TYR	engineered mutation	UNP Q4JR05
A	491	GLY	ARG	engineered mutation	UNP Q4JR05
A	493	GLY	ARG	engineered mutation	UNP Q4JR05
A	494	GLY	ARG	engineered mutation	UNP Q4JR05
A	737	GLY	-	expression tag	UNP Q4JR05
A	738	SER	-	expression tag	UNP Q4JR05
A	739	HIS	-	expression tag	UNP Q4JR05
A	740	HIS	-	expression tag	UNP Q4JR05
A	741	HIS	-	expression tag	UNP Q4JR05
A	742	HIS	-	expression tag	UNP Q4JR05
A	743	HIS	-	expression tag	UNP Q4JR05
A	744	HIS	-	expression tag	UNP Q4JR05
B	180	GLY	TRP	engineered mutation	UNP Q4JR05
B	185	GLY	TYR	engineered mutation	UNP Q4JR05
B	491	GLY	ARG	engineered mutation	UNP Q4JR05
B	493	GLY	ARG	engineered mutation	UNP Q4JR05
B	494	GLY	ARG	engineered mutation	UNP Q4JR05
B	737	GLY	-	expression tag	UNP Q4JR05
B	738	SER	-	expression tag	UNP Q4JR05
B	739	HIS	-	expression tag	UNP Q4JR05
B	740	HIS	-	expression tag	UNP Q4JR05
B	741	HIS	-	expression tag	UNP Q4JR05
B	742	HIS	-	expression tag	UNP Q4JR05
B	743	HIS	-	expression tag	UNP Q4JR05

Continued on next page...

Continued from previous page...

Chain	Residue	Modelled	Actual	Comment	Reference
B	744	HIS	-	expression tag	UNP Q4JR05

- Molecule 2 is an oligosaccharide called alpha-D-mannopyranose-(1-3)-[alpha-D-mannopyranose-(1-6)]alpha-D-mannopyranose-(1-6)-[alpha-D-mannopyranose-(1-3)]beta-D-mannopyranose-(1-4)-2-acetamido-2-deoxy-beta-D-glucopyranose-(1-4)-2-acetamido-2-deoxy-beta-D-glucopyranose.

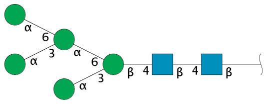

Mol	Chain	Residues	Atoms				ZeroOcc	AltConf	Trace
			Total	C	N	O			
2	C	7	83	46	2	35	0	0	0

- Molecule 3 is an oligosaccharide called alpha-D-mannopyranose-(1-3)-[alpha-D-mannopyranose-(1-6)]beta-D-mannopyranose-(1-4)-2-acetamido-2-deoxy-beta-D-glucopyranose-(1-4)-2-acetamido-2-deoxy-beta-D-glucopyranose.

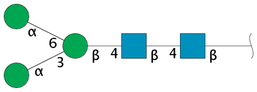

Mol	Chain	Residues	Atoms				ZeroOcc	AltConf	Trace
			Total	C	N	O			
3	D	5	61	34	2	25	0	0	0

- Molecule 4 is an oligosaccharide called 2-acetamido-2-deoxy-beta-D-glucopyranose-(1-4)-2-acetamido-2-deoxy-beta-D-glucopyranose.

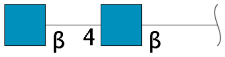

Mol	Chain	Residues	Atoms				ZeroOcc	AltConf	Trace
			Total	C	N	O			
4	E	2	28	16	2	10	0	0	0

- Molecule 5 is 2-acetamido-2-deoxy-beta-D-glucopyranose (three-letter code: NAG) (formula: $C_8H_{15}NO_6$) (labeled as "Ligand of Interest" by author).

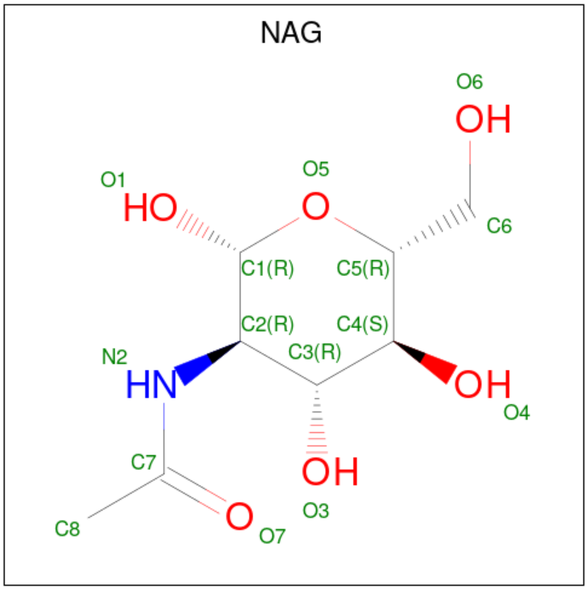

Mol	Chain	Residues	Atoms	ZeroOcc	AltConf
5	A	1	Total C N O<br>14 8 1 5	0	0
5	B	1	Total C N O<br>14 8 1 5	0	0
5	B	1	Total C N O<br>14 8 1 5	0	0

- Molecule 6 is water.

Mol	Chain	Residues	Atoms	ZeroOcc	AltConf
6	A	100	Total O<br>100 100	0	0
6	B	71	Total O<br>71 71	0	0

3 Residue-property plots (i)

These plots are drawn for all protein, RNA, DNA and oligosaccharide chains in the entry. The first graphic for a chain summarises the proportions of the various outlier classes displayed in the second graphic. The second graphic shows the sequence view annotated by issues in geometry and electron density. Residues are color-coded according to the number of geometric quality criteria for which they contain at least one outlier: green = 0, yellow = 1, orange = 2 and red = 3 or more. A red dot above a residue indicates a poor fit to the electron density (RSRZ > 2). Stretches of 2 or more consecutive residues without any outlier are shown as a green connector. Residues present in the sample, but not in the model, are shown in grey.

- Molecule 1: Envelope glycoprotein B

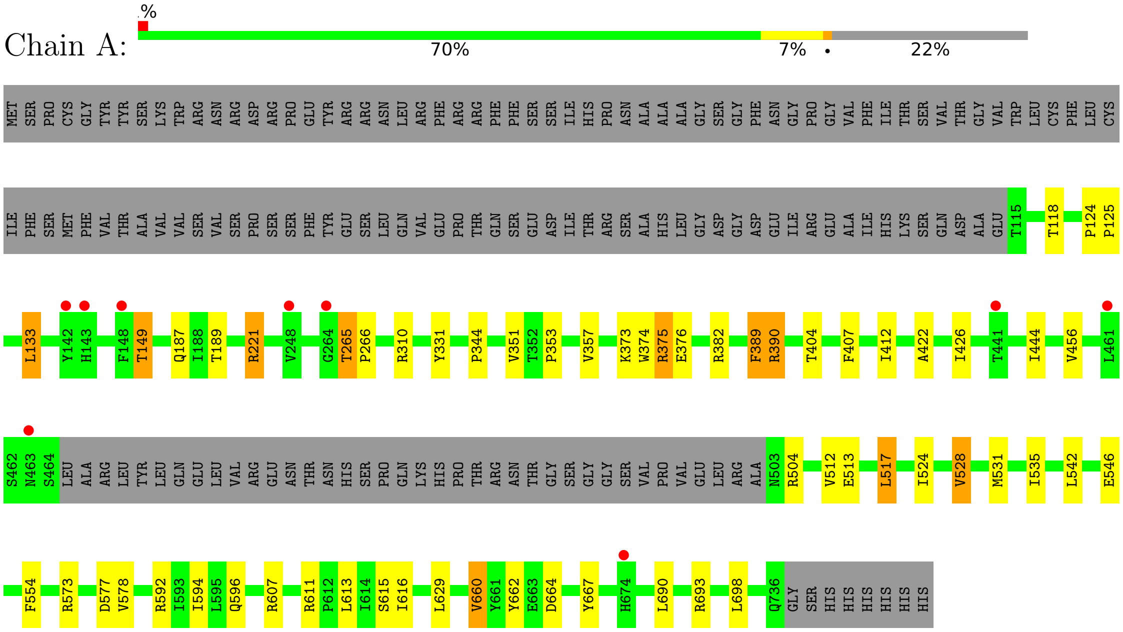

- Molecule 1: Envelope glycoprotein B

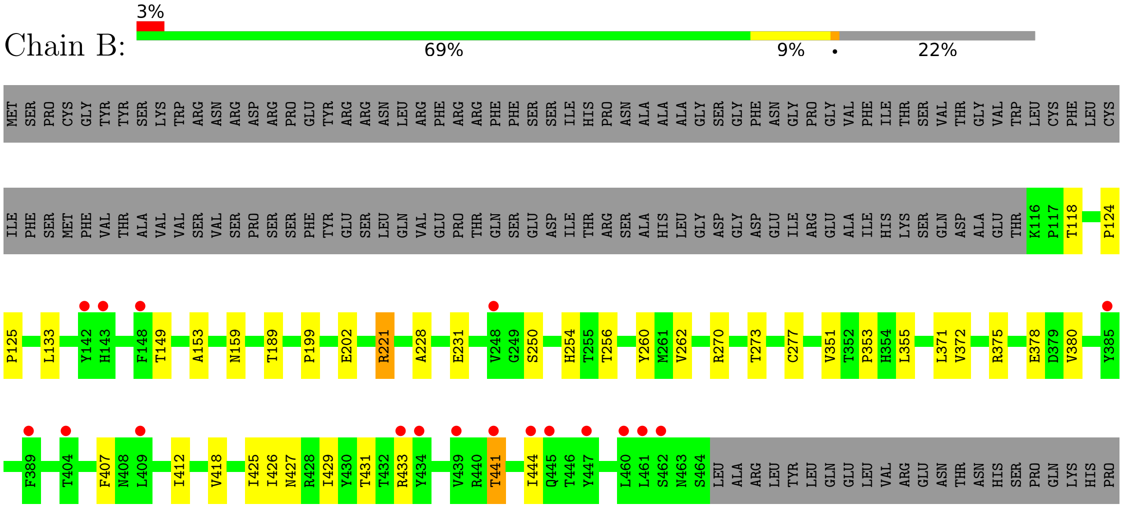

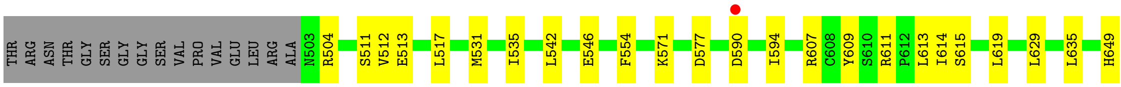

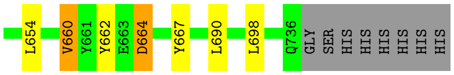

• Molecule 2: alpha-D-mannopyranose-(1-3)-[alpha-D-mannopyranose-(1-6)]alpha-D-mannopyranose-(1-6)-[alpha-D-mannopyranose-(1-3)]beta-D-mannopyranose-(1-4)-2-acetamido-2-deoxy-beta-D-glucopyranose-(1-4)-2-acetamido-2-deoxy-beta-D-glucopyranose

Chain C:

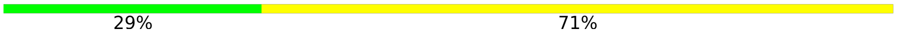

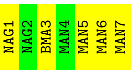

• Molecule 3: alpha-D-mannopyranose-(1-3)-[alpha-D-mannopyranose-(1-6)]beta-D-mannopyranose-(1-4)-2-acetamido-2-deoxy-beta-D-glucopyranose-(1-4)-2-acetamido-2-deoxy-beta-D-glucopyranose

Chain D:

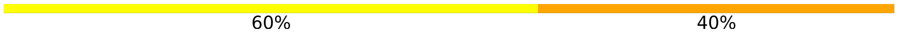

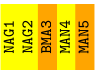

• Molecule 4: 2-acetamido-2-deoxy-beta-D-glucopyranose-(1-4)-2-acetamido-2-deoxy-beta-D-glucopyranose

Chain E:

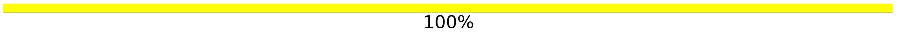

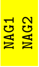

4 Data and refinement statistics (i)

Property	Value	Source
Space group	H 3 2	Depositor
Cell constants<br>a, b, c, α, β, γ	118.32Å 118.32Å 749.03Å<br>90.00° 90.00° 120.00°	Depositor
Resolution (Å)	50.22 – 2.45<br>101.52 – 2.45	Depositor<br>EDS
% Data completeness<br>(in resolution range)	100.0 (50.22-2.45)<br>100.0 (101.52-2.45)	Depositor<br>EDS
R_{merge}	0.07	Depositor
R_{sym}	(Not available)	Depositor
$< I/\sigma(I) >$ [1]	3.10 (at 2.45Å)	Xtriage
Refinement program	PHENIX 1.8.1_1168	Depositor
R, R_{free}	0.181 , 0.228<br>0.184 , 0.229	Depositor<br>DCC
R_{free} test set	3774 reflections (5.04%)	wwPDB-VP
Wilson B-factor (Å^2)	53.6	Xtriage
Anisotropy	0.341	Xtriage
Bulk solvent k_{sol}(e/Å^3), B_{sol}(Å^2)	0.35 , 48.8	EDS
L-test for twinning[2]	$< \|L\| >= 0.47$, $< L^2 >= 0.30$	Xtriage
Estimated twinning fraction	No twinning to report.	Xtriage
F_o,F_c correlation	0.95	EDS
Total number of atoms	9766	wwPDB-VP
Average B, all atoms (Å^2)	43.0	wwPDB-VP

Xtriage's analysis on translational NCS is as follows: *The largest off-origin peak in the Patterson function is 3.79% of the height of the origin peak. No significant pseudotranslation is detected.*

[1]Intensities estimated from amplitudes.

[2]Theoretical values of $< |L| >$, $< L^2 >$ for acentric reflections are 0.5, 0.333 respectively for untwinned datasets, and 0.375, 0.2 for perfectly twinned datasets.

5 Model quality (i)

5.1 Standard geometry (i)

Bond lengths and bond angles in the following residue types are not validated in this section: BMA, NAG, MAN

The Z score for a bond length (or angle) is the number of standard deviations the observed value is removed from the expected value. A bond length (or angle) with $|Z| > 5$ is considered an outlier worth inspection. RMSZ is the root-mean-square of all Z scores of the bond lengths (or angles).

Mol	Chain	Bond lengths		Bond angles	
		RMSZ	$\#\|Z\| >5$	RMSZ	$\#\|Z\| >5$
1	A	0.43	0/4800	0.58	0/6517
1	B	0.42	0/4795	0.55	0/6510
All	All	0.43	0/9595	0.56	0/13027

There are no bond length outliers.

There are no bond angle outliers.

There are no chirality outliers.

There are no planarity outliers.

5.2 Too-close contacts (i)

In the following table, the Non-H and H(model) columns list the number of non-hydrogen atoms and hydrogen atoms in the chain respectively. The H(added) column lists the number of hydrogen atoms added and optimized by MolProbity. The Clashes column lists the number of clashes within the asymmetric unit, whereas Symm-Clashes lists symmetry related clashes.

Mol	Chain	Non-H	H(model)	H(added)	Clashes	Symm-Clashes
1	A	4693	0	4564	25	1
1	B	4688	0	4562	32	1
2	C	83	0	70	0	0
3	D	61	0	52	1	0
4	E	28	0	25	0	0
5	A	14	0	13	0	0
5	B	28	0	26	0	0
6	A	100	0	0	0	0
6	B	71	0	0	0	0
All	All	9766	0	9312	58	2

The all-atom clashscore is defined as the number of clashes found per 1000 atoms (including

hydrogen atoms). The all-atom clashscore for this structure is 3.

All (58) close contacts within the same asymmetric unit are listed below, sorted by their clash magnitude.

Atom-1	Atom-2	Interatomic distance (Å)	Clash overlap (Å)
1:B:221:ARG:NH2	1:B:353:PRO:O	2.21	0.72
1:B:159:ASN:HB2	1:B:372:VAL:HG23	1.72	0.71
1:B:407:PHE:HE2	1:B:412:ILE:HD11	1.55	0.70
1:A:660:VAL:HG22	1:A:667:TYR:HE1	1.61	0.66
1:B:511:SER:OG	1:B:513:GLU:HG2	1.95	0.65
1:B:441:THR:OG1	1:B:441:THR:O	2.11	0.64
1:B:660:VAL:HG22	1:B:667:TYR:HE1	1.65	0.62
1:A:426:ILE:HD12	1:A:444:ILE:HD13	1.86	0.55
1:B:611:ARG:HD3	1:B:629:LEU:O	2.07	0.54
1:A:596:GLN:HB2	1:A:613:LEU:HB2	1.90	0.53
1:A:149:THR:HG21	1:A:382:ARG:HH21	1.75	0.52
1:A:265:THR:HG22	1:A:266:PRO:HD2	1.92	0.51
1:A:374:TRP:CE3	1:A:375:ARG:HB2	2.47	0.50
1:B:426:ILE:HD12	1:B:444:ILE:HD13	1.92	0.50
1:B:531:MET:O	1:B:535:ILE:HG12	2.12	0.49
1:B:199:PRO:HG2	1:B:202:GLU:HG3	1.94	0.49
1:A:373:LYS:NZ	1:A:376:GLU:OE2	2.35	0.49
1:B:254:HIS:HD2	1:B:256:THR:O	1.96	0.49
1:A:221:ARG:NH2	1:A:353:PRO:O	2.32	0.48
1:A:422:ALA:HB1	1:A:456:VAL:HG11	1.97	0.47
1:A:660:VAL:HG13	1:A:662:TYR:HE1	1.79	0.47
1:B:228:ALA:HB1	1:B:273:THR:HG21	1.97	0.47
1:A:407:PHE:HE2	1:A:412:ILE:HD11	1.80	0.46
1:B:649:HIS:HB3	1:B:664:ASP:HA	1.97	0.46
1:A:611:ARG:HD3	1:A:629:LEU:O	2.14	0.46
1:B:371:LEU:HD12	1:B:418:VAL:HG21	1.98	0.45
1:A:124:PRO:HA	1:A:125:PRO:HD3	1.84	0.45
1:B:609:TYR:CE2	1:B:613:LEU:HD11	2.52	0.45
1:B:594:ILE:CG1	1:B:615:SER:HB2	2.46	0.45
1:B:231:GLU:HG2	1:B:260:TYR:CD1	2.52	0.44
1:B:629:LEU:HD21	1:B:654:LEU:O	2.17	0.44
1:B:614:ILE:HD13	1:B:635:LEU:HD22	1.98	0.44
1:A:407:PHE:CE2	1:A:412:ILE:HD11	2.53	0.44
1:A:542:LEU:O	1:A:546:GLU:HG2	2.18	0.44
1:B:124:PRO:HA	1:B:125:PRO:HD3	1.89	0.43
1:B:542:LEU:O	1:B:546:GLU:HG2	2.19	0.43
1:A:389:PHE:CD2	1:A:404:THR:HA	2.53	0.43
1:A:133:LEU:HD12	1:A:133:LEU:HA	1.82	0.43

Continued on next page...

Continued from previous page...

Atom-1	Atom-2	Interatomic distance (Å)	Clash overlap (Å)
1:B:660:VAL:HG13	1:B:662:TYR:HE1	1.85	0.42
1:B:125:PRO:HD3	1:B:571:LYS:HD3	2.01	0.42
1:B:407:PHE:CE2	1:B:412:ILE:HD11	2.45	0.42
1:B:590:ASP:HB2	1:B:619:LEU:HB2	2.01	0.42
1:A:125:PRO:HD2	1:A:573:ARG:NH1	2.35	0.42
1:B:425:ILE:O	1:B:429:ILE:HG12	2.20	0.42
1:A:531:MET:O	1:A:535:ILE:HG12	2.19	0.41
1:A:331:TYR:O	1:A:344:PRO:HA	2.20	0.41
1:A:517:LEU:HD23	1:A:517:LEU:HA	1.80	0.41
3:D:3:BMA:H61	3:D:5:MAN:H2	1.64	0.41
1:B:378:GLU:HG2	1:B:429:ILE:HD12	2.01	0.41
1:B:427:ASN:O	1:B:431:THR:HG23	2.21	0.41
1:B:262:VAL:HG21	1:B:270:ARG:NH2	2.36	0.41
1:A:660:VAL:HG13	1:A:662:TYR:CE1	2.56	0.41
1:A:382:ARG:HA	1:A:390:ARG:O	2.21	0.41
1:A:594:ILE:HG13	1:A:615:SER:HB2	2.02	0.41
1:A:524:ILE:O	1:A:528:VAL:HG13	2.21	0.41
1:B:153:ALA:HB3	1:B:380:VAL:HG21	2.02	0.41
1:B:254:HIS:HA	1:B:277:CYS:O	2.20	0.40
1:B:660:VAL:CG1	1:B:662:TYR:HE1	2.33	0.40

All (2) symmetry-related close contacts are listed below. The label for Atom-2 includes the symmetry operator and encoded unit-cell translations to be applied.

Atom-1	Atom-2	Interatomic distance (Å)	Clash overlap (Å)
1:A:577:ASP:OD1	1:A:611:ARG:NH2[2_565]	2.15	0.05
1:B:577:ASP:OD1	1:B:611:ARG:NH2[3_555]	2.18	0.02

5.3 Torsion angles (i)

5.3.1 Protein backbone (i)

In the following table, the Percentiles column shows the percent Ramachandran outliers of the chain as a percentile score with respect to all X-ray entries followed by that with respect to entries of similar resolution.

The Analysed column shows the number of residues for which the backbone conformation was analysed, and the total number of residues.

Mol	Chain	Analysed	Favoured	Allowed	Outliers	Percentiles	
1	A	580/744 (78%)	568 (98%)	12 (2%)	0	100	100
1	B	579/744 (78%)	567 (98%)	12 (2%)	0	100	100
All	All	1159/1488 (78%)	1135 (98%)	24 (2%)	0	100	100

There are no Ramachandran outliers to report.

5.3.2 Protein sidechains (i)

In the following table, the Percentiles column shows the percent sidechain outliers of the chain as a percentile score with respect to all X-ray entries followed by that with respect to entries of similar resolution.

The Analysed column shows the number of residues for which the sidechain conformation was analysed, and the total number of residues.

Mol	Chain	Analysed	Rotameric	Outliers	Percentiles	
1	A	520/662 (78%)	492 (95%)	28 (5%)	22	28
1	B	520/662 (78%)	500 (96%)	20 (4%)	33	43
All	All	1040/1324 (78%)	992 (95%)	48 (5%)	27	35

All (48) residues with a non-rotameric sidechain are listed below:

Mol	Chain	Res	Type
1	A	118	THR
1	A	133	LEU
1	A	149	THR
1	A	187	GLN
1	A	189	THR
1	A	221	ARG
1	A	265	THR
1	A	310	ARG
1	A	351	VAL
1	A	357	VAL
1	A	375	ARG
1	A	389	PHE
1	A	390	ARG
1	A	504	ARG
1	A	512	VAL
1	A	513	GLU
1	A	517	LEU
1	A	528	VAL

Continued on next page...

Continued from previous page...

Mol	Chain	Res	Type
1	A	554	PHE
1	A	578	VAL
1	A	592	ARG
1	A	607	ARG
1	A	616	ILE
1	A	660	VAL
1	A	664	ASP
1	A	690	LEU
1	A	693	ARG
1	A	698	LEU
1	B	118	THR
1	B	133	LEU
1	B	149	THR
1	B	189	THR
1	B	221	ARG
1	B	250	SER
1	B	351	VAL
1	B	355	LEU
1	B	375	ARG
1	B	433	ARG
1	B	441	THR
1	B	504	ARG
1	B	512	VAL
1	B	517	LEU
1	B	554	PHE
1	B	607	ARG
1	B	660	VAL
1	B	664	ASP
1	B	690	LEU
1	B	698	LEU

Some sidechains can be flipped to improve hydrogen bonding and reduce clashes. There are no such sidechains identified.

5.3.3 RNA (i)

There are no RNA molecules in this entry.

5.4 Non-standard residues in protein, DNA, RNA chains (i)

There are no non-standard protein/DNA/RNA residues in this entry.

5.5 Carbohydrates (i)

14 monosaccharides are modelled in this entry.

In the following table, the Counts columns list the number of bonds (or angles) for which Mogul statistics could be retrieved, the number of bonds (or angles) that are observed in the model and the number of bonds (or angles) that are defined in the Chemical Component Dictionary. The Link column lists molecule types, if any, to which the group is linked. The Z score for a bond length (or angle) is the number of standard deviations the observed value is removed from the expected value. A bond length (or angle) with $|Z| > 2$ is considered an outlier worth inspection. RMSZ is the root-mean-square of all Z scores of the bond lengths (or angles).

Mol	Type	Chain	Res	Link	Bond lengths			Bond angles		
					Counts	RMSZ	$\#\|Z\| > 2$	Counts	RMSZ	$\#\|Z\| > 2$
2	NAG	C	1	1,2	14,14,15	0.62	0	17,19,21	1.13	3 (17%)
2	NAG	C	2	2	14,14,15	0.71	0	17,19,21	0.87	0
2	BMA	C	3	2	11,11,12	0.88	0	15,15,17	1.13	1 (6%)
2	MAN	C	4	2	11,11,12	0.58	0	15,15,17	0.79	0
2	MAN	C	5	2	11,11,12	0.62	0	15,15,17	1.10	1 (6%)
2	MAN	C	6	2	11,11,12	0.65	0	15,15,17	0.93	1 (6%)
2	MAN	C	7	2	11,11,12	0.68	0	15,15,17	0.92	1 (6%)
3	NAG	D	1	1,3	14,14,15	0.55	0	17,19,21	1.17	2 (11%)
3	NAG	D	2	3	14,14,15	0.57	0	17,19,21	1.24	3 (17%)
3	BMA	D	3	3	11,11,12	0.75	0	15,15,17	1.29	2 (13%)
3	MAN	D	4	3	11,11,12	0.51	0	14,15,17	1.16	1 (7%)
3	MAN	D	5	3	11,11,12	0.67	0	15,15,17	1.27	1 (6%)
4	NAG	E	1	1,4	14,14,15	0.47	0	17,19,21	1.71	1 (5%)
4	NAG	E	2	4	14,14,15	0.57	0	17,19,21	1.12	1 (5%)

In the following table, the Chirals column lists the number of chiral outliers, the number of chiral centers analysed, the number of these observed in the model and the number defined in the Chemical Component Dictionary. Similar counts are reported in the Torsion and Rings columns. '-' means no outliers of that kind were identified.

Mol	Type	Chain	Res	Link	Chirals	Torsions	Rings
2	NAG	C	1	1,2	-	0/6/23/26	0/1/1/1
2	NAG	C	2	2	-	0/6/23/26	0/1/1/1
2	BMA	C	3	2	-	0/2/19/22	0/1/1/1
2	MAN	C	4	2	-	2/2/19/22	0/1/1/1
2	MAN	C	5	2	-	1/2/19/22	0/1/1/1
2	MAN	C	6	2	-	0/2/19/22	0/1/1/1
2	MAN	C	7	2	-	2/2/19/22	0/1/1/1

Continued on next page...

Continued from previous page...

Mol	Type	Chain	Res	Link	Chirals	Torsions	Rings
3	NAG	D	1	1,3	-	0/6/23/26	0/1/1/1
3	NAG	D	2	3	-	2/6/23/26	0/1/1/1
3	BMA	D	3	3	-	2/2/19/22	0/1/1/1
3	MAN	D	5	3	-	2/2/19/22	0/1/1/1
4	NAG	E	1	1,4	-	0/6/23/26	0/1/1/1
4	NAG	E	2	4	-	0/6/23/26	0/1/1/1

There are no bond length outliers.

All (18) bond angle outliers are listed below:

Mol	Chain	Res	Type	Atoms	Z	Observed(°)	Ideal(°)
4	E	1	NAG	C1-O5-C5	6.36	120.81	112.19
3	D	5	MAN	C1-C2-C3	4.09	114.69	109.67
3	D	3	BMA	C1-O5-C5	3.46	116.88	112.19
3	D	4	MAN	C1-C2-C3	-3.27	105.64	109.67
4	E	2	NAG	O5-C5-C6	2.83	111.64	107.20
3	D	2	NAG	O4-C4-C3	-2.82	103.82	110.35
2	C	5	MAN	O5-C5-C6	2.81	111.62	107.20
2	C	7	MAN	O5-C5-C6	2.53	111.18	107.20
2	C	1	NAG	O5-C1-C2	-2.43	107.44	111.29
2	C	1	NAG	O4-C4-C5	-2.39	103.36	109.30
3	D	1	NAG	C3-C4-C5	2.24	114.23	110.24
3	D	2	NAG	C4-C3-C2	2.24	114.30	111.02
3	D	1	NAG	O5-C1-C2	-2.16	107.88	111.29
2	C	1	NAG	O5-C5-C6	2.15	110.57	107.20
2	C	3	BMA	C1-O5-C5	2.13	115.08	112.19
2	C	6	MAN	O5-C1-C2	-2.12	107.50	110.77
3	D	3	BMA	O2-C2-C3	-2.09	105.96	110.14
3	D	2	NAG	C2-N2-C7	-2.01	120.04	122.90

There are no chirality outliers.

All (11) torsion outliers are listed below:

Mol	Chain	Res	Type	Atoms
2	C	4	MAN	O5-C5-C6-O6
3	D	2	NAG	C8-C7-N2-C2
3	D	3	BMA	C4-C5-C6-O6
3	D	2	NAG	O7-C7-N2-C2
3	D	3	BMA	O5-C5-C6-O6
2	C	4	MAN	C4-C5-C6-O6

Continued on next page...

Continued from previous page...

Mol	Chain	Res	Type	Atoms
3	D	5	MAN	C4-C5-C6-O6
3	D	5	MAN	O5-C5-C6-O6
2	C	5	MAN	O5-C5-C6-O6
2	C	7	MAN	C4-C5-C6-O6
2	C	7	MAN	O5-C5-C6-O6

There are no ring outliers.

2 monomers are involved in 1 short contact:

Mol	Chain	Res	Type	Clashes	Symm-Clashes
3	D	3	BMA	1	0
3	D	5	MAN	1	0

The following is a two-dimensional graphical depiction of Mogul quality analysis of bond lengths, bond angles, torsion angles, and ring geometry for oligosaccharide.

Oligosaccharide Chain C

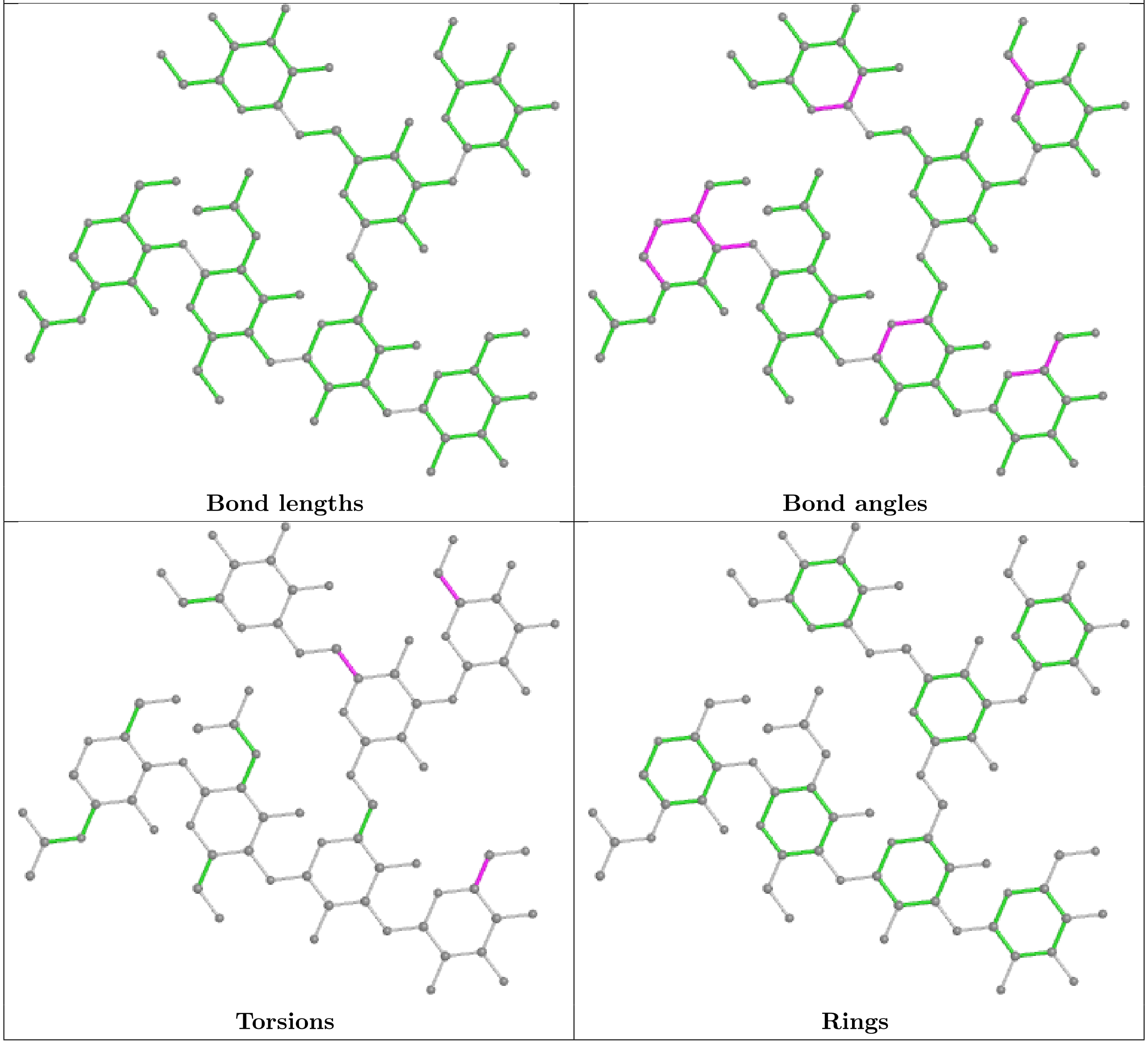

Oligosaccharide Chain D

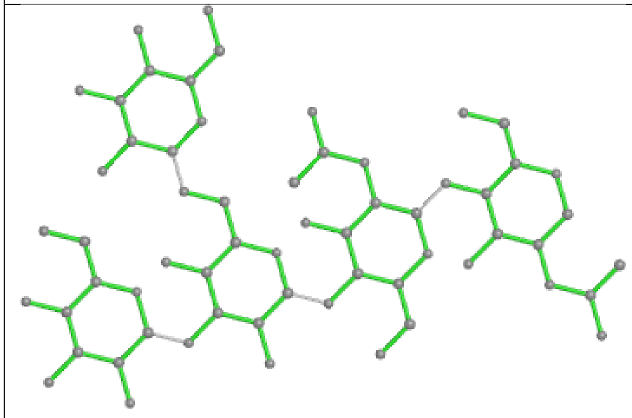

Bond lengths

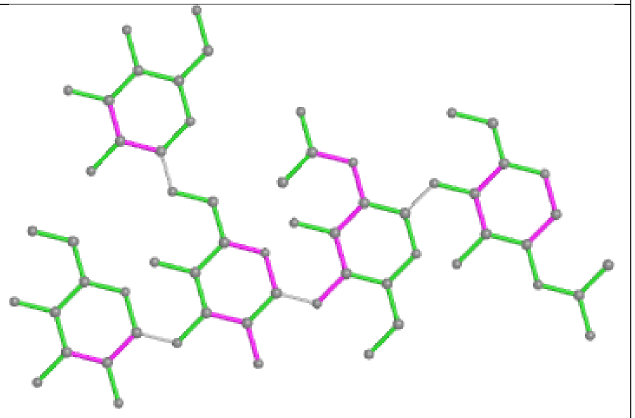

Bond angles

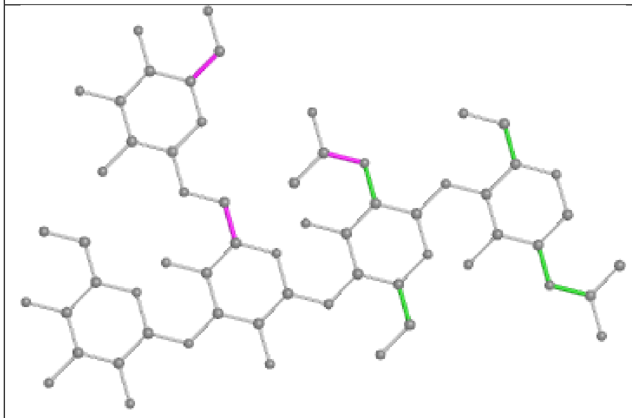

Torsions

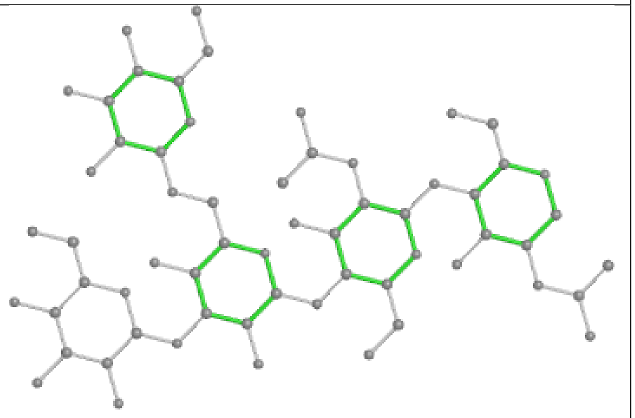

Rings

Oligosaccharide Chain E

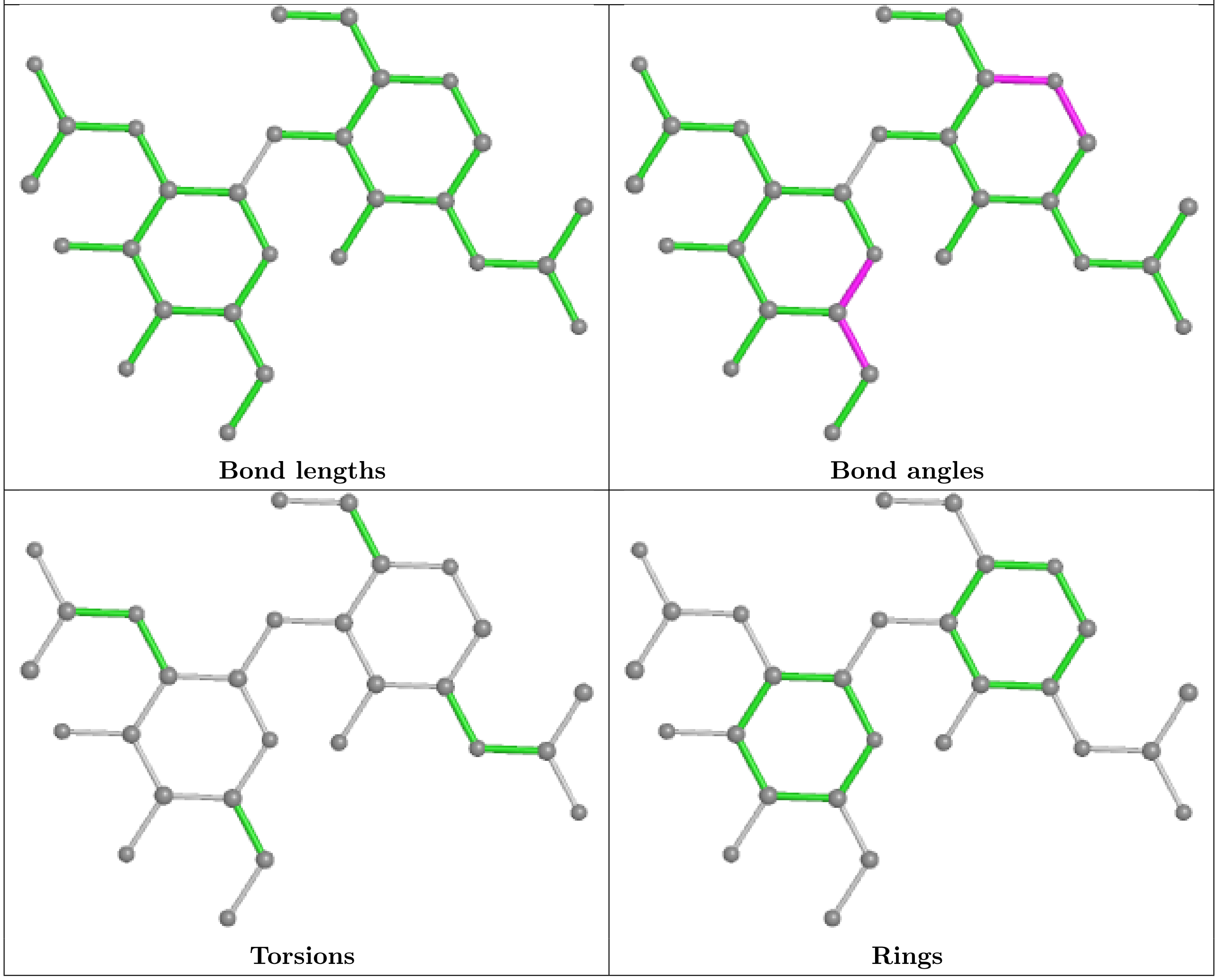

5.6 Ligand geometry (i)

3 ligands are modelled in this entry.

In the following table, the Counts columns list the number of bonds (or angles) for which Mogul statistics could be retrieved, the number of bonds (or angles) that are observed in the model and the number of bonds (or angles) that are defined in the Chemical Component Dictionary. The Link column lists molecule types, if any, to which the group is linked. The Z score for a bond length (or angle) is the number of standard deviations the observed value is removed from the expected value. A bond length (or angle) with $|Z| > 2$ is considered an outlier worth inspection. RMSZ is the root-mean-square of all Z scores of the bond lengths (or angles).

Mol	Type	Chain	Res	Link	Bond lengths			Bond angles		
					Counts	RMSZ	$\#\|Z\| > 2$	Counts	RMSZ	$\#\|Z\| > 2$
5	NAG	B	804	1	14,14,15	0.45	0	17,19,21	1.23	2 (11%)
5	NAG	A	813	1	14,14,15	0.50	0	17,19,21	1.02	2 (11%)
5	NAG	B	801	1	14,14,15	0.55	0	17,19,21	1.47	2 (11%)

In the following table, the Chirals column lists the number of chiral outliers, the number of chiral centers analysed, the number of these observed in the model and the number defined in the Chemical Component Dictionary. Similar counts are reported in the Torsion and Rings columns. '-' means no outliers of that kind were identified.

Mol	Type	Chain	Res	Link	Chirals	Torsions	Rings
5	NAG	B	804	1	-	4/6/23/26	0/1/1/1
5	NAG	A	813	1	-	2/6/23/26	0/1/1/1
5	NAG	B	801	1	-	4/6/23/26	0/1/1/1

There are no bond length outliers.

All (6) bond angle outliers are listed below:

Mol	Chain	Res	Type	Atoms	Z	Observed(^{o})	Ideal(^{o})
5	B	801	NAG	C4-C3-C2	3.79	116.57	111.02
5	B	804	NAG	C1-O5-C5	3.31	116.68	112.19
5	B	801	NAG	O5-C1-C2	2.88	115.83	111.29
5	A	813	NAG	O5-C5-C6	2.67	111.39	107.20
5	B	804	NAG	O5-C1-C2	2.08	114.58	111.29
5	A	813	NAG	C1-O5-C5	2.05	114.98	112.19

There are no chirality outliers.

All (10) torsion outliers are listed below:

Mol	Chain	Res	Type	Atoms
5	B	801	NAG	C8-C7-N2-C2
5	B	801	NAG	O7-C7-N2-C2
5	A	813	NAG	O5-C5-C6-O6
5	A	813	NAG	C4-C5-C6-O6
5	B	804	NAG	C8-C7-N2-C2
5	B	804	NAG	O7-C7-N2-C2
5	B	804	NAG	C1-C2-N2-C7
5	B	804	NAG	C3-C2-N2-C7
5	B	801	NAG	C4-C5-C6-O6
5	B	801	NAG	O5-C5-C6-O6

There are no ring outliers.

No monomer is involved in short contacts.

The following is a two-dimensional graphical depiction of Mogul quality analysis of bond lengths, bond angles, torsion angles, and ring geometry for all instances of the Ligand of Interest. In addition, ligands with molecular weight > 250 and outliers as shown on the validation Tables will also be included. For torsion angles, if less then 5% of the Mogul distribution of torsion angles is

within 10 degrees of the torsion angle in question, then that torsion angle is considered an outlier. Any bond that is central to one or more torsion angles identified as an outlier by Mogul will be highlighted in the graph. For rings, the root-mean-square deviation (RMSD) between the ring in question and similar rings identified by Mogul is calculated over all ring torsion angles. If the average RMSD is greater than 60 degrees and the minimal RMSD between the ring in question and any Mogul-identified rings is also greater than 60 degrees, then that ring is considered an outlier. The outliers are highlighted in purple. The color gray indicates Mogul did not find sufficient equivalents in the CSD to analyse the geometry.

Ligand NAG B 804

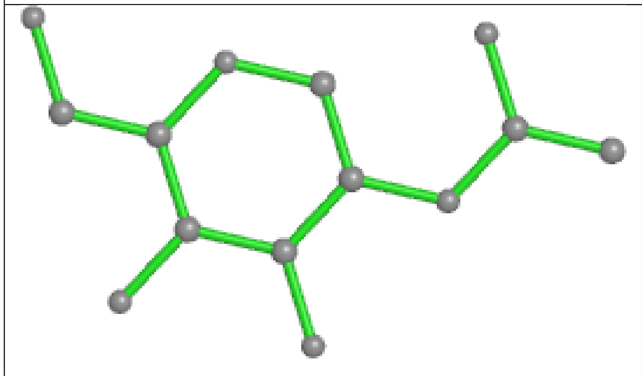

Bond lengths

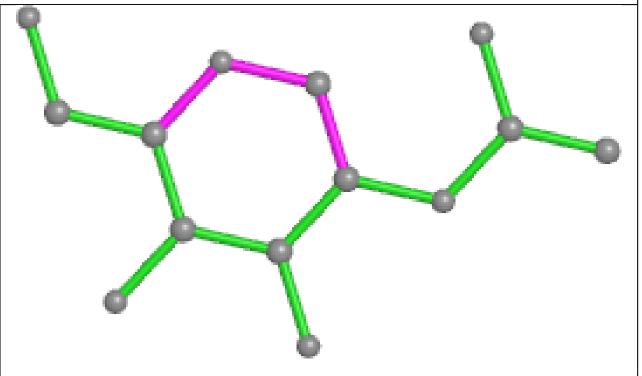

Bond angles

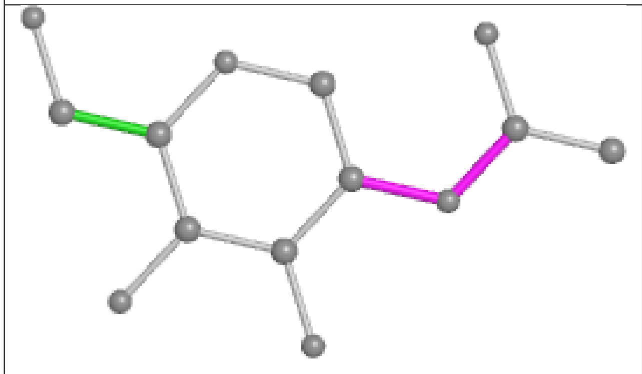

Torsions

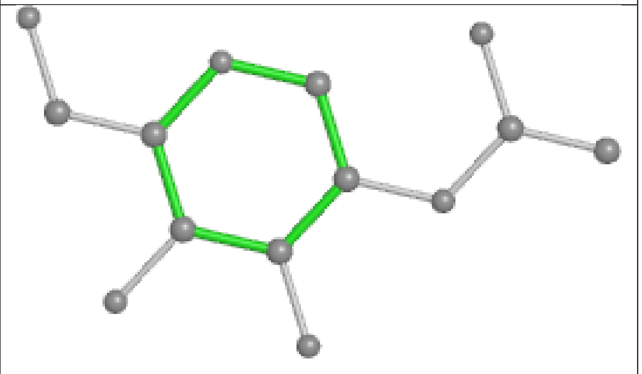

Rings

Ligand NAG A 813

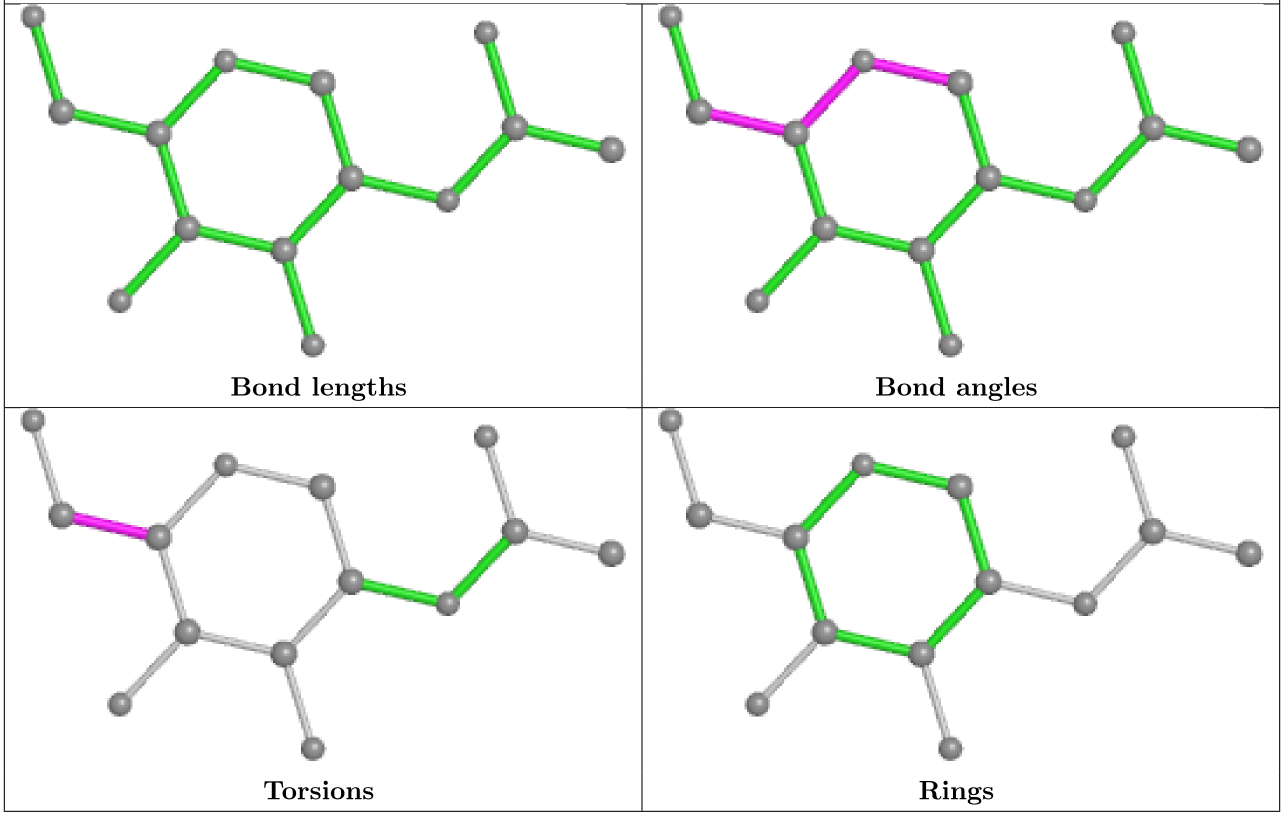

Ligand NAG B 801

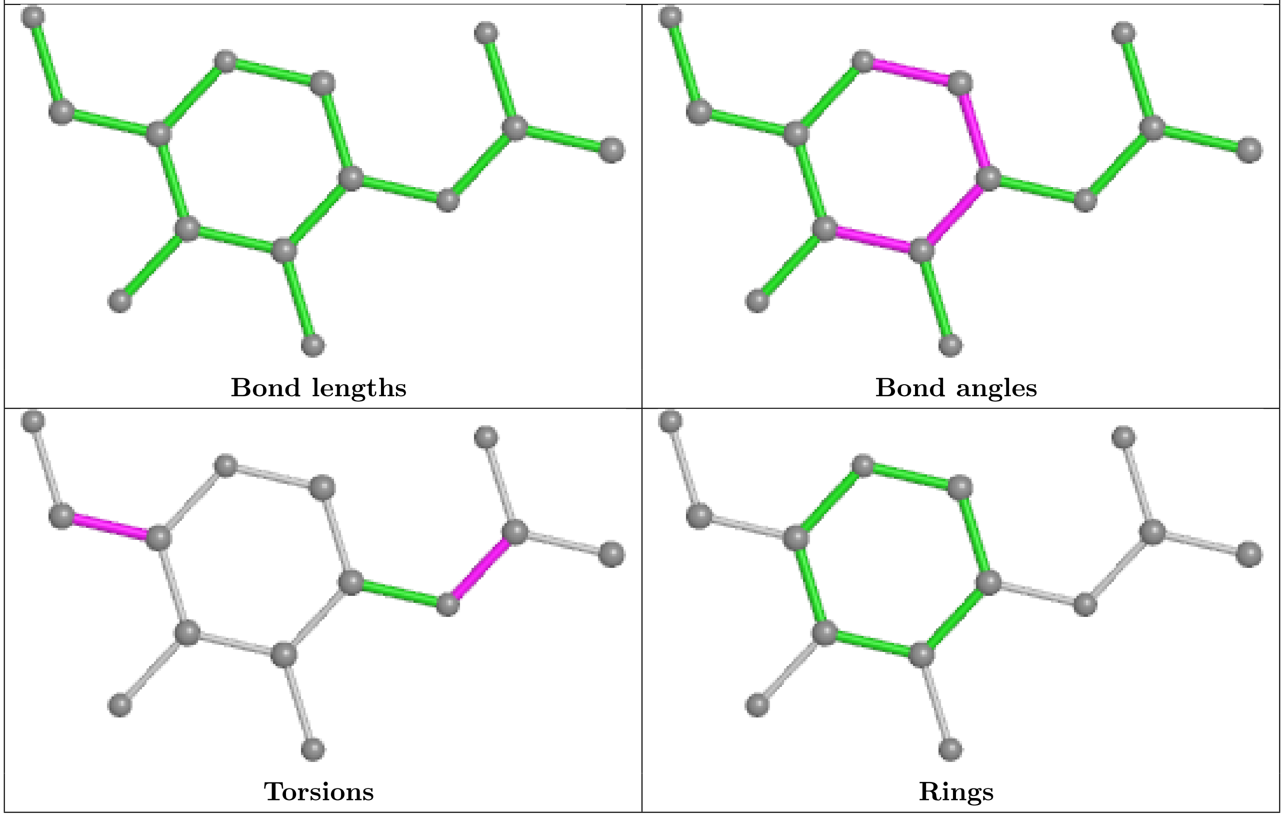

5.7 Other polymers (i)

There are no such residues in this entry.

5.8 Polymer linkage issues (i)

There are no chain breaks in this entry.

6 Fit of model and data (i)

6.1 Protein, DNA and RNA chains (i)

In the following table, the column labelled '#RSRZ> 2' contains the number (and percentage) of RSRZ outliers, followed by percent RSRZ outliers for the chain as percentile scores relative to all X-ray entries and entries of similar resolution. The OWAB column contains the minimum, median, 95th percentile and maximum values of the occupancy-weighted average B-factor per residue. The column labelled 'Q< 0.9' lists the number of (and percentage) of residues with an average occupancy less than 0.9.

Mol	Chain	Analysed	<RSRZ>	#RSRZ>2			OWAB(Å^2)	Q<0.9
1	A	584/744 (78%)	0.16	9 (1%)	73	71	16, 35, 75, 98	0
1	B	583/744 (78%)	0.20	19 (3%)	46	43	18, 41, 85, 130	0
All	All	1167/1488 (78%)	0.18	28 (2%)	59	54	16, 38, 81, 130	0

All (28) RSRZ outliers are listed below:

Mol	Chain	Res	Type	RSRZ
1	B	143	HIS	5.4
1	B	433	ARG	3.5
1	B	462	SER	3.3
1	B	439	VAL	3.3
1	B	248	VAL	3.2
1	B	142	TYR	3.2
1	A	142	TYR	3.1
1	B	590	ASP	3.1
1	B	461	LEU	3.1
1	B	444	ILE	3.0
1	B	385	TYR	3.0
1	A	148	PHE	2.8
1	A	143	HIS	2.8
1	A	264	GLY	2.7
1	B	404	THR	2.5
1	B	148	PHE	2.5
1	A	248	VAL	2.5
1	A	463	ASN	2.4
1	A	461	LEU	2.4
1	A	441	THR	2.3
1	B	389	PHE	2.3
1	B	460	LEU	2.2
1	B	447	TYR	2.2
1	A	674	HIS	2.1

Continued on next page...

Continued from previous page...

Mol	Chain	Res	Type	RSRZ
1	B	441	THR	2.1
1	B	445	GLN	2.1
1	B	434	TYR	2.1
1	B	409	LEU	2.0

6.2 Non-standard residues in protein, DNA, RNA chains (i)

There are no non-standard protein/DNA/RNA residues in this entry.

6.3 Carbohydrates (i)

In the following table, the Atoms column lists the number of modelled atoms in the group and the number defined in the chemical component dictionary. The B-factors column lists the minimum, median, 95th percentile and maximum values of B factors of atoms in the group. The column labelled 'Q< 0.9' lists the number of atoms with occupancy less than 0.9.

Mol	Type	Chain	Res	Atoms	RSCC	RSR	B-factors(Å^2)	Q<0.9
4	NAG	E	1	14/15	0.63	0.36	72,87,94,101	0
3	NAG	D	2	14/15	0.70	0.44	99,118,131,136	0
3	MAN	D	4	11/12	0.70	0.67	114,134,140,140	0
3	BMA	D	3	11/12	0.75	0.49	136,141,143,144	0
3	MAN	D	5	11/12	0.76	0.64	123,133,136,138	0
4	NAG	E	2	14/15	0.83	0.39	88,105,111,111	0
3	NAG	D	1	14/15	0.84	0.39	69,82,95,107	0
2	MAN	C	6	11/12	0.89	0.15	85,94,98,100	0
2	MAN	C	4	11/12	0.90	0.19	90,100,106,109	0
2	MAN	C	5	11/12	0.90	0.18	95,108,110,111	0
2	MAN	C	7	11/12	0.91	0.15	85,93,98,101	0
2	BMA	C	3	11/12	0.92	0.16	45,59,72,75	0
2	NAG	C	1	14/15	0.97	0.17	23,33,42,46	0
2	NAG	C	2	14/15	0.98	0.18	32,41,46,49	0

The following is a graphical depiction of the model fit to experimental electron density for oligosaccharide. Each fit is shown from different orientation to approximate a three-dimensional view.

Electron density around Chain C:

2mF$_o$-DF$_c$ (at 0.7 rmsd) in gray
mF$_o$-DF$_c$ (at 3 rmsd) in purple (negative)
and green (positive)

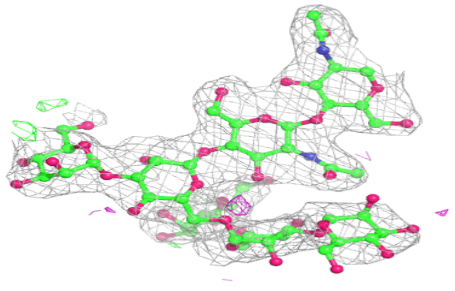

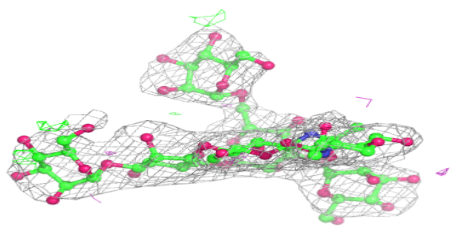

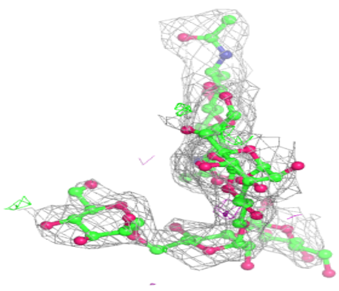

Electron density around Chain D:

2mF$_o$-DF$_c$ (at 0.7 rmsd) in gray
mF$_o$-DF$_c$ (at 3 rmsd) in purple (negative)
and green (positive)

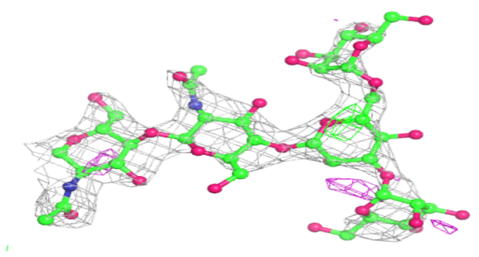

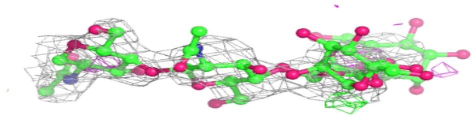

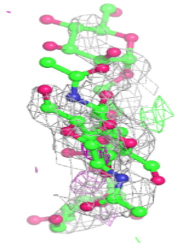

Electron density around Chain E:

2mF$_o$-DF$_c$ (at 0.7 rmsd) in gray
mF$_o$-DF$_c$ (at 3 rmsd) in purple (negative)
and green (positive)

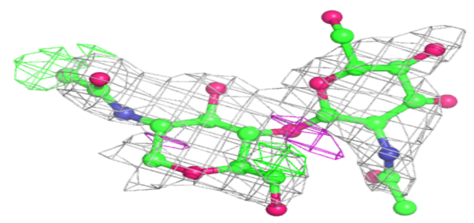

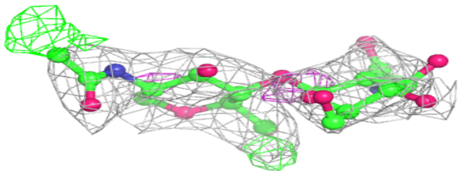

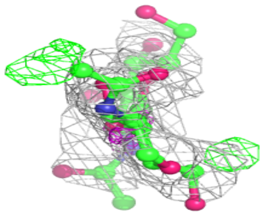

6.4 Ligands ⓘ

In the following table, the Atoms column lists the number of modelled atoms in the group and the number defined in the chemical component dictionary. The B-factors column lists the minimum, median, 95th percentile and maximum values of B factors of atoms in the group. The column labelled 'Q< 0.9' lists the number of atoms with occupancy less than 0.9.

Mol	Type	Chain	Res	Atoms	RSCC	RSR	B-factors(Å^2)	Q<0.9
5	NAG	B	801	14/15	0.81	0.18	67,79,83,86	0
5	NAG	A	813	14/15	0.83	0.19	100,104,107,108	0
5	NAG	B	804	14/15	0.85	0.26	93,107,117,117	0

The following is a graphical depiction of the model fit to experimental electron density of all instances of the Ligand of Interest. In addition, ligands with molecular weight > 250 and outliers as shown on the geometry validation Tables will also be included. Each fit is shown from different orientation to approximate a three-dimensional view.

Electron density around NAG B 801:

$2mF_o$-DF_c (at 0.7 rmsd) in gray
mF_o-DF_c (at 3 rmsd) in purple (negative)
and green (positive)

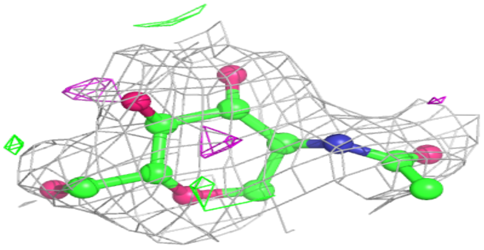

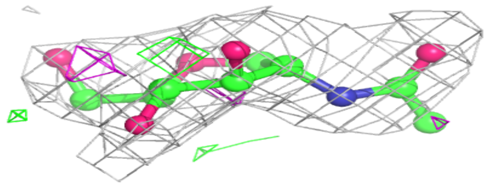

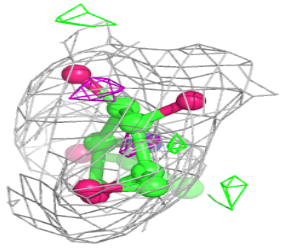

Electron density around NAG A 813:

$2mF_o$-DF_c (at 0.7 rmsd) in gray
mF_o-DF_c (at 3 rmsd) in purple (negative)
and green (positive)

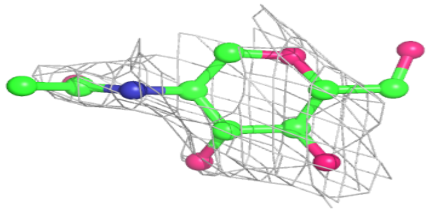

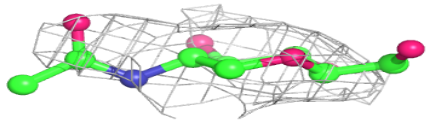

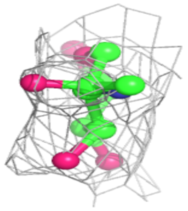

Electron density around NAG B 804:

2mF$_o$-DF$_c$ (at 0.7 rmsd) in gray
mF$_o$-DF$_c$ (at 3 rmsd) in purple (negative)
and green (positive)

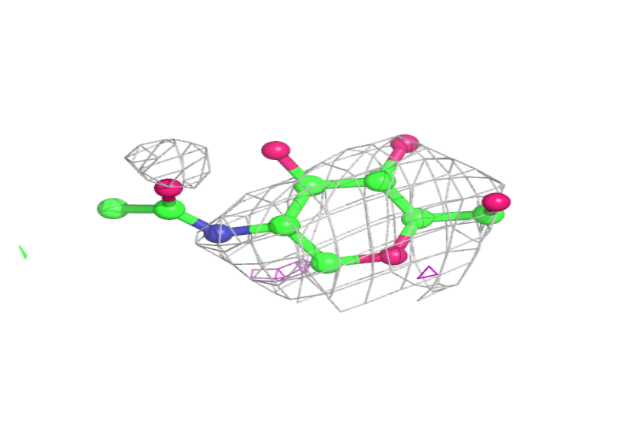

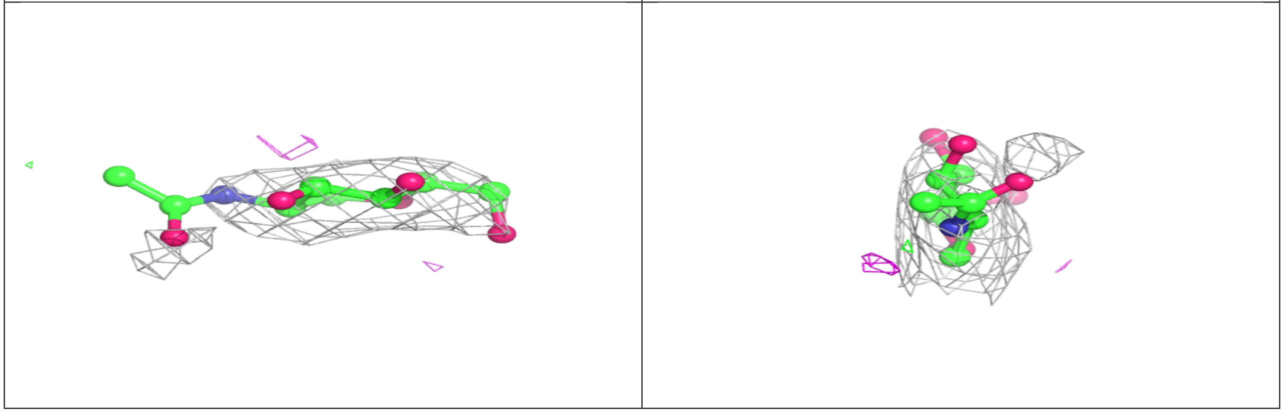

6.5 Other polymers (i)

There are no such residues in this entry.
