# Supplementary material for: The N-terminus of varicella-zoster virus glycoprotein B has a functional role in fusion
Source: PLoS Pathog. 2021 Jan 7;17(1):e1008961. doi: 10.1371/journal.ppat.1008961 (PMC7817050; doi:10.1371/journal.ppat.1008961)
Supplement: Attachment — Submitted filename: PPATHOGENS-D-20-02004_ReviewersComments_22nov20_01.docx [file ppat.1008961.s023.docx]

Reviewer's Responses to Questions

**Part I - Summary**
Please use this section to discuss strengths/weaknesses of study, novelty/significance, general execution and scholarship.

Reviewer #1: This work uses structural and functional evidence to establish the importance of the N-terminus of VZV gB for fusion. The paper reports three new structures: the crystal structure of the VZV gB ectodomain, a cryoEM structure of full-length VZV gB including the cytoplasmic domain, and a cryoEM structure of VZV gB bound to a non-neutralizing MAb SG2. Comparison of gB bound to nAb 93K (examined by cryoEM here and independently in a recent paper) and gB bound to non-nAb SG2 shows that the Ab binding sites are distinct but overlapping. The analysis suggests that the primary site of non-nAb SG2 binding (β25-26) is not critical for gB function, whereas a binding site exclusive to nAb 93K (at the N-terminus) is important for gB function. The structural data leads to a hypothesis that the unresolved flexible gB N-terminus is important for fusion. A predicted helix in the N-terminus (residues 109-112), just upstream from the 93k binding site, is demonstrated to be critical for cell-cell fusion function by site-specific mutagenesis. When mutations at this site are added to virus, virus growth is impaired. The work supports a model that the N-terminus of gB, a target of neutralizing antibodies, is required for the gB conformational change that mediates fusion during virus entry. The study is logically presented and well described. These new structures and the study of function derived from them contribute to the model of herpesvirus entry.

*Author response: We thank you for your clear synopsis and your conclusion that we have achieved our goal to contribute to the understanding herpesvirus entry.*

Reviewer #2: This manuscript describes a tour de force characterization of the ectodomain of VZV gB. The authors are commended on their achievement. Their interesting results have led to a few questions that could not have been asked earlier.

*Author response: We thank you for your exceptionally positive assessment of our work.*

1. Introduction, line 73. To avoid confusion by HSV scientists, mention that VZV gB is cleaved. Also tell us the MW of the gB product before cleavage and the MW of the two cleavage products.

*Author response: Thank you for this suggestion. We think the placement of the VZV gB cleavage did not fit well in the suggested section. However, we have amended the following paragraph to contain the information requested at Lines 85-87 “Unlike the closely related herpes simplex virus gB orthologues, the mature glycosylated form of VZV gB (~130kDa) is cleaved into two fragments (~69kDa and ~73kDa) at the ^491^RSRR^494^ furin recognition site.”*

2. Results, line 126. The different anti-gB antibody reagents are mentioned at the beginning of the Results. When the reader goes to the Method section, there is only a brief sentence to describe the antibodies. Because of their importance in this study recommend that the authors prepare a separate section in Methods for the 3 Antibodies. Describe the origin of each antibody, even if already described elsewhere. Give IgG subclass if known. Give 1 or 2 references to each antibody, if available.

*Author response: Thank you for this suggestion. We have provided a more detailed description of the antibodies in the Materials and Methods in a new section “Anti-VZV gB antibodies.” at Lines 450-457. We have also added a section describing the preparation of Fabs bound to gB as this section was omitted from the original manuscript; Lines 517-527.*

3. Figure 10C in Results. Almost all VZV strains are highly fusogenic when grown in human melanoma cells. In contrast, VZV induces only small polykaryons in infected fibroblast cells. Did the authors compare syncytial formation and plaque sizes of their mutant gB viruses between melanoma cells and fibroblast cells? They may also want to comment on this differential in the Discussion. Is there a missing cellular factor in some cells that enhances (or inhibits) VZV-induced fusion and that is unknown to us?

*Author response: Thank you for your insightful thoughts. We have not performed experiments comparing these new mutants in different cell lines. As you note, syncytia formation is more apparent in MeWo cells compared to fibroblasts. MeWo cells are used to model the polykaryocyte formation seen in infected skin tissue [1, 2]. Fibroblasts are very useful for studies of VZV replication in vitro but they are not a primary target cell in vivo. Interestingly, T cells, which become infected during the course of disease in people and transport VZV to the skin, do not undergo cell-cell fusion* [3-5]*. It is currently not clear why there are differences in polykaryocyte formation in different cell types. We suspect that the differences are attributed to cellular factors. In our recently published work, we have identified a cellular factor, calcineurin, that is important for regulating VZV induced cell-cell fusion [6]. It is plausible that other cellular factors influence the VZV induced syncytia phenotype in different cell lines. This is an active area of research for us.*

4. Figure 10F. This panel is extremely important. The authors state that all VZV gB mutants retained the capacity to form a complex with gH:gL. This finding deserves a few more sentences of description in the Results. This finding also deserves a few more sentences of comparison with HSV gB and gH:gL in the Discussion. In particular, haven’t the authors shown that VZV gB is the true fusogen, thereby confirming earlier results about HSV gB from Eisenberg/Cohen lab? Eisenburg/Cohen proposed that gD assisted gH:gL, and in turn gH:gL assisted gB to become the true fusogen. Is that the conclusion of these authors (Obviously VZV has no gD)? Or is the above statement a misinterpretation of their VZV data? Perhaps add a few comments in Discussion about the D. Atanasiu et al 2016 Journal of Virology article about the cascade of events of the HSV glycoproteins (PMID: 27630245). Also, please add small numbered arrows to each row of bands in each of the 3 smaller panels within panel 10F, to better identify the individual proteins; and expand the legend to panel 10F.

*Author response: We agree that the data presented in Fig. 10F are important and these data support the concept that has been asserted for the fusion complex of other herpesviruses. Although this confirmation of previous findings is important, it is not a central thesis to the current manuscript but rather supporting evidence that the mutagenesis of the VZV gB N-terminus does not affect the gB/gH-gL interaction. In addition, we agree that gB is considered to be the primary fusogen. However, it is largely non-functional in the absence of the gH-gL heterodimer, which is required for receptor engagement, tropism and gB activation for herpesviruses in general [7-22]. We have referenced our previous findings which suggest that the role of VZV gH-gL is highly likely to be similar [23-26]. Thus, although VZV gB is considered the primary fusogen, the interaction with and activation by gH-gL supports a more complex mechanism. As stated, we recognize that this concept is important be we do not think that additional discussion is justified in the current manuscript.*

*We have edited the panels in Fig. 10F to better identify the individual proteins in each panel and have updated the figure legend.*

Reviewer #3: This MS reports a cryo-EM structure of the postfusion conformation of VZV gB bound with a non-neutralizing mouse monoclonal Fab, SG2, and compares the result with a previously published structure of the same protein bound with Fab from a neutralizing human mAb, 93k. Both antibodies bind DIV and have partly overlapping epitopes, but the footprint of 93k is adjacent to a proximal segment of the N-terminal region, which is poorly ordered from the N-terminus to residue 115 (the first well-ordered residue in the structure). The proximity focused the authors on a short peptide, residues 109-112. Mutation of those residues to alanine greatly diminished spread in an assay that depends on gB-mediated cell-cell fusion. The authors conclude that the N-terminal segment contributes to the fusion mechanism.

*Author response: Thank you for this concise overview.*

The key problem, of course, is that this is the post-fusion structure, and neutralization by inhibiting fusion depends on binding the prefusion conformer. The prefusion structures of gB from HCMV and HSV have been determined by subtomogram averaging to quite reasonable resolutions, and in those structures DIV is very close to the fusion loops. So the arguments here are irrelevant. At least the authors should have take their structures of DIV bound with the Fabs and considered them in the context of a homology model for prefusion VZV gB. The meaning of the effect of mutation on residues 109-112 is likewise uninterpretable from just the postfusion structure.

*Author response: Thank you for raising this point. A critical concept that arose from the conclusion of our recently published near-atomic resolution structure (2.8Å) of the human neutralizing mAb 93k bound to VZV gB is that the epitope for 93k, although present on the postfusion conformation of gB, must also be accessible on the prefusion form of VZV gB for 93k to be neutralizing [27]. Our previous data also clearly establish that gB domain IV, the binding site of 93k, must retain its conformation in both the prefusion and postfusion forms of gB. At the time we submitted our manuscript there was not a clear understanding of the orientations and placement of the herpesvirus gB domains in the two highest resolution (21Å and 23Å) structures of the reported prefusion forms of gB [28, 29]. Zeev-Ben-Mordehai reported a ‘loops out’ model and Si reported a ‘loops in’ model. This did not provide us with the necessary data and confidence to present compelling homology models of VZV gB. Fortunately, a higher resolution structure of HSV gB has been recently reported based on cryo-ET at 9.0Å resolution [30]. These findings support the ‘loops in’ model of gB, which contrasts with the authors’ previous findings. This information has made it possible for us to add substantially to our structural analysis of VZV gB presented in this manuscript. Based on Vollmer’s 9.0Å cryo-EM map and model of HSV gB, we have now added a homology model of VZV gB that clearly demonstrates why mAb 93k is neutralizing and SG2 is not (Fig. 11; S4 Movie); owing to the angle of SG2 binding along with the inversion of gB domain IV and its proximity to the VZV envelope, SG2 is unlikely to bind to the prefusion form of VZV gB. Furthermore, our VZV gB prefusion homology model also provides strong evidence as to why the N-terminus of gB plays a critical role in membrane fusion, supporting the findings in our current manuscript. The N-terminus is exposed on the external side of the prefusion conformation in close proximity to the 93k binding site, as expected.*

**Part II – Major Issues: Key Experiments Required for Acceptance**
Please use this section to detail the key new experiments or modifications of existing experiments that should be absolutely required to validate study conclusions.

Generally, there should be no more than 3 such required experiments or major modifications for a "Major Revision" recommendation. If more than 3 experiments are necessary to validate the study conclusions, then you are encouraged to recommend "Reject".

Reviewer #1: The authors demonstrate that all of the gB mutants coIP with V5-tagged gH/gL (Fig. 10F). A negative control lacking gH/gL is included, but a specificity control including an unrelated V5-tagged protein (or a gB mutant that does not interact with gH/gL) is not. If the coIP procedure pulls down membrane fragments, gB could be detected in any sample that includes gH/gL, regardless of whether each gB mutant interacts with gHgL. Specificity is especially a concern when using over-expressed constructs of gB and gHgL. If this gB-gH/gL coIP is an established protocol, the authors can include a citation and explanation.

*Author response: Thank you for this insightful comment. We are in agreement and have performed additional experiments to demonstrate the specificity of the gB/gH-gL interaction. To demonstrate this specificity the VZV gE/gI complex was co-expressed with either gB/gH-WT/gL or gB/gH-V5/gL. Antibodies to gB (mAb 93k), V5 and gE, readily detected these proteins by western blot of lysates from transfected cells. However, upon using the V5 agarose to immuneprecipitate the gB/gH-gL complex, gE was not detected (S3 Fig.). This demonstrates that the gB/gH-gL interaction is specific and the over-expression system used does not result in membrane fragments pulled down with the V5 agarose that contain nonspecific glycoproteins such as gE and gI. We have added these experiments to the results section to support our findings at Lines 295-300 “The gH-gL heterodimer is critical for the fusion function of gB. The VZV gB/gH-gL fusion complex can be immunoprecipitated using mAb 93k or an anti-V5 mAb to bind gH-V5. To demonstrate the specificity of this interaction, CHO cells were co-transfecting with the gB/gH-gL vectors along with the pBud-gE/gI vector, which simultaneously expresses VZV gE and gI. Although gE was abundantly expressed upon transfection, it did not co-immunoprecipitate with the gB/gH-gL complex (S3 Fig.).”*

Reviewer #2: No new experiments are requested in my comments in Part I.

*Author response: Our responses have been provided in Part I.*

Reviewer #3: See summary. This reviewer does not believe that the MS contains enough new information to be worthy of publication.

*Author response: The authors respectfully disagree with this assessment. The relevance of our findings about VZV gB are strongly supported by the comments from the other reviewers and we have reinforced their evaluations with new data. In addition, because of the publication by Vollmer et al., we have been able to carry out new modeling not possible from the lower resolution herpesvirus gB structures that had been reported previously. This provides insights about VZV gB DIV as a prefusion structure based comparing models with our structures of gB with Fabs of the neutralizing and non-neutralizing antibodies. Further, it supports our prior experimental findings that the epitope targeted by the neutralizing 93k mAb is accessible both pre- and post-fusion [27].*

**Part III – Minor Issues: Editorial and Data Presentation Modifications**
Please use this section for editorial suggestions as well as relatively minor modifications of existing data that would enhance clarity.

Reviewer #1: The Q111A and D112A mutants were greatly impaired (<10%) in cell-cell fusion but not in virus growth. In contrast, the K109A mutant was modestly impaired (42%) in cell-cell fusion, but the mutation was not tolerated for virus growth. The authors suggest that the poor function of gB-Q111A and gB-D112A in cell-cell fusion when compared to virus growth may indicate that N-terminal gB mutations are tolerated better when other glycoproteins are present (line 276). This explanation does not explain why the K109A phenotype was reversed (i.e. K109A was better tolerated in the cell-cell fusion assay than the virus assay). Can the authors clarify? Even more interestingly, the K109R mutant functioned at near wild-type levels in cell-cell fusion but was not tolerated for virus growth. What other differences between cell-cell fusion and virus-cell fusion could account for that? Are the authors confident in their BAC sequences?

*Author response: Thank you for these questions. Firstly, we would like to address the comment about the BAC sequences. We are very confident that the BAC sequences are correct. Firstly, all of our BACs purified after the 2^nd^ Red recombination are evaluated by HindII digest. There are also three tiers of quality control in our mutagenesis strategy where sanger sequencing is performed to check that spurious mutations have not arisen in the mutated ORF, in this case ORF31[gB]; the mutant gB-Kan cassette (direct sequencing of the plasmid), the mutant BAC (direct sequencing of the BAC), the virus stocks (sequencing of PCR products amplified from ORF31 in DNA extracted from MeWo cells infected with the mutants and wild type VZV). We also used multiple BAC clones and performed multiple transfections to derive the mutant viruses to show reproducibility. Because K109A and K109R did not yield infectious virus these experiments were performed seven (K109A) and four (K109R) times. We also note that although infectious VZV was recovered for the ^109^AAAA^112^ mutant this was not a trivial process. In contrast to the typical 7-10 days to recover infectious VZV and produce stocks, it took several weeks before the ^109^AAAA^112^ could be propagated. The small plaque virus that was recovered did have a non-synonymous G to A transition leading to a glycine (G) to glutamic acid (E) substitution (S2 Fig. has been updated to make this clearer). We have speculated that this substitution led to a compensatory mutation offsetting the inactivating properties of the ^109^AAAA^112^ mutation. Although rare, we have observed this phenomenon previously in our mutagenesis studies of VZV gH [23].*

*Critically, our experiments demonstrate the importance of using different functional assays to determine the effects of mutagenesis; in this context, using both cell-cell fusion and VZV genome mutagenesis. The discrepancies between the cell-cell fusion assay and productive virus propagation in MeWo cells is not uncommon in our experience [2, 23, 27, 31]. As the cell-cell fusion assay is based on just the core glycoproteins required for cell-cell fusion, gB, gH and gL, it is highly likely that these glycoproteins will be influenced by the other VZV glycoproteins in the virion envelope; gC, gE, gK, gL, gN and gM. How these glycoproteins might affect the VZV gB/gH-gL fusion complex in the context of the virion is currently unclear. In addition, other non-membrane bound VZV proteins could also influence entry fusion. In light of the newly published cryo-ET structure of HSV gB in a prefusion form, the location of the N-terminus does appear to place the ^109^KSQD^112^ region in an exposed area where interactions with other viral or extracellular proteins could occur. Although we cannot provide a definitive hypothesis on how the mutants behave in different assays, perhaps one explanation might be that the N-terminus interacts with other VZV membrane proteins to either enable or limit membrane fusion. However, these questions are poised for future studies.*

The authors propose that 93k may lock gB in a prefusion state (line 346). 93K must bind to prefusion gB (or an intermediate form) since it is neutralizing, but the structure shows that 93K bound to a postfusion form of gB. Can the authors add to their discussion here, including the idea that 93K can bind to both forms but still neutralize?

*Author response: This is a very intriguing question and one we now think has been answered by the recent publication of the cryo-ET structure of HSV gB in a prefusion form. We have added a new figure and movie (Fig. 11; S4 Movie) that demonstrate the likely location of 93k binding to VZV gB in a prefusion form. This modelling supports our previous statements about 93k locking gB in a prefusion conformation and the role of binding to the VZV gB N-terminus in preventing the transition to the postfusion form of gB.*

93K is able to bind to the AAAA mutant (Fig. 10B). Do the authors think that the AAAA mutant could be trapped in prefusion? I realize no data are available to answer this question, but I am interested in the authors’ perspectives.

*Author response: Thanks for another intriguing question. Although we have not currently determined whether the ^109^AAAA^112^ mutant is locked in a prefusion form it is a possibility. However, SG2 does bind to the ^109^AAAA^112^ mutant in our cell surface expression assays. As we show in our newly revised manuscript, SG2 is unlikely to be able to bind effectively to the prefusion form of VZV gB. Thus, the ^109^AAAA^112^ mutant is not likely to be locked in a prefusion form, suggesting that the N-terminus has additional functions in its role in cell-cell fusion. In addition, the recovery of infectious virus from the ^109^AAAA^112^ mutant, albeit difficult to propagate, also supports the notion that gB is not locked in a prefusion conformation.*

Please makes sure that the cryoEM micrographs are at as high of resolution as possible. Seeing an identifiable morphology for the individual particles by eye may not be possible (for example in Fig. 1B), but it would be nice if the reader could.

*Author response: The cryo-EM micrograph in Fig. 1B has been updated so that particles are more easily visible.*

Figure 2 legend, line 946: The authors could clarify what is meant by the line "The model is not used for gB-93k."

*Author response: This statement was from a previous version of our manuscript. We have removed the text from the figure legend.*

Fig. 4B, line 946: The legend says that western blots of gB probed with 93k and SG2 are shown, but only the PAb western is shown. The legend can be updated.

*Author response: The figure legend has been updated.*

Fig 5 legend, line 964: The title of the figure refers only to the full-length gB, not the crystal structure in parts F-H.

*Author response: The title has been updated.*

Fig. 8: The N-terminal region that is more ordered in PRV and HSV Low pH would be clearer if it were colored.

*Author response: The figure has been updated to highlight the ordered domains of PRV and low pH HSV.*

Fig. 10A: In the strongest magnification (on the far right), showing only the sticks may make the figure clearer. Coloring/shading the N-terminus portion of DIV may also help show how 93k binds to the N-terminus.

*Author response: As the region of the N-terminus that was mutated was not resolved in our structure data it was not possible to show this region in the figure. However, we have now updated the figure to show where 93k interacts with the portion of the N-terminus that was resolved in our cryo-EM and X-ray crystallography data.*

Fig. 10 legend, liene 1036: The legend refers to + and - signs that are not in the figure.

*Author response: Thank you for spotting this oversight, Fig. 10C has been updated.*

Reviewer #2: The minor issues that need to be addressed are cited in my comments in Part I.

*Author response: Our responses have been addressed in Part I.*

Reviewer #3: (No Response)

**References**

1.	Oliver SL, Brady JJ, Sommer MH, Reichelt M, Sung P, Blau HM, et al. An immunoreceptor tyrosine-based inhibition motif in varicella-zoster virus glycoprotein B regulates cell fusion and skin pathogenesis. Proc Natl Acad Sci U S A. 2013;110(5):1911-6.

2.	Yang E, Arvin AM, Oliver SL. The cytoplasmic domain of varicella-zoster virus glycoprotein H regulates syncytia formation and skin pathogenesis. PLoS Pathog. 2014;10(5):e1004173.

3.	Moffat JF, Stein MD, Kaneshima H, Arvin AM. Tropism of varicella-zoster virus for human CD4+ and CD8+ T lymphocytes and epidermal cells in SCID-hu mice. J Virol. 1995;69(9):5236-42.

4.	Moffat JF, Zerboni L, Sommer MH, Heineman TC, Cohen JI, Kaneshima H, et al. The ORF47 and ORF66 putative protein kinases of varicella-zoster virus determine tropism for human T cells and skin in the SCID-hu mouse. Proc Natl Acad Sci U S A. 1998;95(20):11969-74.

5.	Ku CC, Zerboni L, Ito H, Graham BS, Wallace M, Arvin AM. Varicella-zoster virus transfer to skin by T Cells and modulation of viral replication by epidermal cell interferon-alpha. J Exp Med. 2004;200(7):917-25.

6.	Zhou M, Kamarshi V, Arvin AM, Oliver SL. Calcineurin phosphatase activity regulates Varicella-Zoster Virus induced cell-cell fusion. PLOS Pathogens. 2020;16(11):e1009022.

7.	Atanasiu D, Saw WT, Cohen GH, Eisenberg RJ. Cascade of Events Governing Cell-Cell Fusion Induced by Herpes Simplex Virus Glycoproteins gD, gH/gL, and gB. Journal of Virology. 2010;84(23):12292-9.

8.	Turner A, Bruun B, Minson T, Browne H. Glycoproteins gB, gD, and gHgL of herpes simplex virus type 1 are necessary and sufficient to mediate membrane fusion in a Cos cell transfection system. Journal of Virology. 1998;72(1):873-5.

9.	Akkapaiboon P, Mori Y, Sadaoka T, Yonemoto S, Yamanishi K. Intracellular processing of human herpesvirus 6 glycoproteins Q1 and Q2 into tetrameric complexes expressed on the viral envelope. J Virol. 2004;78(15):7969-83.

10.	Connolly SA, Whitbeck JJ, Rux AH, Krummenacher C, van Drunen Littel-van den Hurk S, Cohen GH, et al. Glycoprotein D homologs in herpes simplex virus type 1, pseudorabies virus, and bovine herpes virus type 1 bind directly to human HveC(nectin-1) with different affinities. Virology. 2001;280(1):7-18.

11.	Geraghty RJ, Krummenacher C, Cohen GH, Eisenberg RJ, Spear PG. Entry of alphaherpesviruses mediated by poliovirus receptor-related protein 1 and poliovirus receptor. Science. 1998;280(5369):1618-20.

12.	Hahn G, Revello MG, Patrone M, Percivalle E, Campanini G, Sarasini A, et al. Human cytomegalovirus UL131-128 genes are indispensable for virus growth in endothelial cells and virus transfer to leukocytes. J Virol. 2004;78(18):10023-33.

13.	Jiang XJ, Adler B, Sampaio KL, Digel M, Jahn G, Ettischer N, et al. UL74 of human cytomegalovirus contributes to virus release by promoting secondary envelopment of virions. J Virol. 2008;82(6):2802-12.

14.	Montgomery RI, Warner MS, Lum BJ, Spear PG. Herpes simplex virus-1 entry into cells mediated by a novel member of the TNF/NGF receptor family. Cell. 1996;87(3):427-36.

15.	Mori Y, Akkapaiboon P, Yang X, Yamanishi K. The human herpesvirus 6 U100 gene product is the third component of the gH-gL glycoprotein complex on the viral envelope. J Virol. 2003;77(4):2452-8.

16.	Ryckman BJ, Chase MC, Johnson DC. HCMV gH/gL/UL128-131 interferes with virus entry into epithelial cells: evidence for cell type-specific receptors. Proc Natl Acad Sci U S A. 2008;105(37):14118-23.

17.	Ryckman BJ, Jarvis MA, Drummond DD, Nelson JA, Johnson DC. Human cytomegalovirus entry into epithelial and endothelial cells depends on genes UL128 to UL150 and occurs by endocytosis and low-pH fusion. J Virol. 2006;80(2):710-22.

18.	Tanaka Y, Suenaga T, Matsumoto M, Seya T, Arase H. Herpesvirus 6 glycoproteins B (gB), gH, gL, and gQ are necessary and sufficient for cell-to-cell fusion. J Virol. 2013;87(19):10900-3.

19.	Wang D, Shenk T. Human cytomegalovirus UL131 open reading frame is required for epithelial cell tropism. J Virol. 2005;79(16):10330-8.

20.	Wang X, Kenyon WJ, Li Q, Mullberg J, Hutt-Fletcher LM. Epstein-Barr virus uses different complexes of glycoproteins gH and gL to infect B lymphocytes and epithelial cells. J Virol. 1998;72(7):5552-8.

21.	Wille PT, Knoche AJ, Nelson JA, Jarvis MA, Johnson DC. A human cytomegalovirus gO-null mutant fails to incorporate gH/gL into the virion envelope and is unable to enter fibroblasts and epithelial and endothelial cells. J Virol. 2010;84(5):2585-96.

22.	Zhou M, Lanchy JM, Ryckman BJ. Human Cytomegalovirus gH/gL/gO Promotes the Fusion Step of Entry into All Cell Types, whereas gH/gL/UL128-131 Broadens Virus Tropism through a Distinct Mechanism. J Virol. 2015;89(17):8999-9009.

23.	Vleck SE, Oliver SL, Brady JJ, Blau HM, Rajamani J, Sommer MH, et al. Structure-function analysis of varicella-zoster virus glycoprotein H identifies domain-specific roles for fusion and skin tropism. Proc Natl Acad Sci U S A. 2011;108(45):18412-7.

24.	Vleck SE, Oliver SL, Reichelt M, Rajamani J, Zerboni L, Jones C, et al. Anti-glycoprotein H antibody impairs the pathogenicity of varicella-zoster virus in skin xenografts in the SCID mouse model. J Virol. 2010;84(1):141-52.

25.	Xing Y, Oliver SL, Nguyen T, Ciferri C, Nandi A, Hickman J, et al. A site of varicella-zoster virus vulnerability identified by structural studies of neutralizing antibodies bound to the glycoprotein complex gHgL. Proc Natl Acad Sci U S A. 2015;112(19):6056-61.

26.	Yang E, Arvin AM, Oliver SL. Role for the alphaV Integrin Subunit in Varicella-Zoster Virus-Mediated Fusion and Infection. J Virol. 2016;90(16):7567-78.

27.	Oliver SL, Xing Y, Chen DH, Roh SH, Pintilie GD, Bushnell DA, et al. A glycoprotein B-neutralizing antibody structure at 2.8 A uncovers a critical domain for herpesvirus fusion initiation. Nat Commun. 2020;11(1):4141.

28.	Zeev-Ben-Mordehai T, Vasishtan D, Hernandez Duran A, Vollmer B, White P, Prasad Pandurangan A, et al. Two distinct trimeric conformations of natively membrane-anchored full-length herpes simplex virus 1 glycoprotein B. Proc Natl Acad Sci U S A. 2016;113(15):4176-81.

29.	Si Z, Zhang J, Shivakoti S, Atanasov I, Tao CL, Hui WH, et al. Different functional states of fusion protein gB revealed on human cytomegalovirus by cryo electron tomography with Volta phase plate. PLoS Pathog. 2018;14(12):e1007452.

30.	Vollmer B, Prazak V, Vasishtan D, Jefferys EE, Hernandez-Duran A, Vallbracht M, et al. The prefusion structure of herpes simplex virus glycoprotein B. Sci Adv. 2020;6(39).

31.	Yang E, Arvin AM, Oliver SL. The Glycoprotein B Cytoplasmic Domain Lysine Cluster Is Critical for Varicella-Zoster Virus Cell-Cell Fusion Regulation and Infection. J Virol. 2017;91(1).
